# Supplementary material for: The effect of intravenous hyoscine butylbromide on slow progress in labor (BUSCLAB): A double-blind randomized placebo-controlled trial
Source: PLoS Med. 2024 Mar 28;21(3):e1004352. doi: 10.1371/journal.pmed.1004352 (PMC11008832; doi:10.1371/journal.pmed.1004352)
Supplement: S1 CONSORT Checklist — (PDF) [file pmed.1004352.s001.pdf]

CONSORT 2010 checklist of information to include when reporting a randomised trial*

Section/Topic	Item No	Checklist item	Reported on page No
**Title and abstract**			
	1a	Identification as a randomised trial in the title	Present in title
	1b	Structured summary of trial design, methods, results, and conclusions (for specific guidance see CONSORT for abstracts)	Present in abstract
**Introduction**			
Background and objectives	2a	Scientific background and explanation of rationale	Section: Introduction, paragraph: 1-3
	2b	Specific objectives or hypotheses	Section: Introduction paragraph: 3-4
**Methods**			
Trial design	3a	Description of trial design (such as parallel, factorial) including allocation ratio	Section: Methods, subsection Study design Paragraph: 1-2 and in subsection Randomization and blinding in subparagraph 1
	3b	Important changes to methods after trial commencement (such as eligibility criteria), with reasons	No changes were made

			after start of trial
Participants	4a	Eligibility criteria for participants	Section: Methods Paragraph: 3, 4, 5 and 6
	4b	Settings and locations where the data were collected	Section: Methods, Participants Paragraph: 3
Interventions	5	The interventions for each group with sufficient details to allow replication, including how and when they were actually administered	Section: Methods, subsection Randomizatio n and blinding in paragraph 2-3
Outcomes	6a	Completely defined pre-specified primary and secondary outcome measures, including how and when they were assessed	Section: Methods, subsection Outcomes Paragraph in subsection: 1-4
	6b	Any changes to trial outcomes after the trial commenced, with reasons	Not applicable
Sample size	7a	How sample size was determined	Section: Methods, subsection Sample size calculations. Subsection paragraph: 1
	7b	When applicable, explanation of any interim analyses and stopping guidelines	Not applicable

Randomisation:			
Sequence generation	8a	Method used to generate the random allocation sequence	Section: Randomization and blinding Paragraph: 1
	8b	Type of randomisation; details of any restriction (such as blocking and block size)	Section: Randomization and blinding Paragraph: 1
Allocation concealment mechanism	9	Mechanism used to implement the random allocation sequence (such as sequentially numbered containers), describing any steps taken to conceal the sequence until interventions were assigned	Section: Randomization and blinding Paragraph: 1, 2 and 3
Implementation	10	Who generated the random allocation sequence, who enrolled participants, and who assigned participants to interventions	Section: Randomization and blinding Paragraph: 1 and 2
Blinding	11a	If done, who was blinded after assignment to interventions (for example, participants, care providers, those assessing outcomes) and how	Section: Randomization and blinding Paragraph: 3. In addition in Abstract in paragraph of methods and findings.
	11b	If relevant, description of the similarity of interventions	Not applicable
Statistical methods	12a	Statistical methods used to compare groups for primary and secondary outcomes	Section: Statistical analysis Paragraph: 1-

			9
	12b	Methods for additional analyses, such as subgroup analyses and adjusted analyses	Section: Methods, subsection analysis sets and statistical analysis.
**Results**			
Participant flow (a diagram is strongly recommended)	13a	For each group, the numbers of participants who were randomly assigned, received intended treatment, and were analysed for the primary outcome	Fig2 CONSORT Flow Diagram
	13b	For each group, losses and exclusions after randomisation, together with reasons	Fig2 CONSORT Flow Diagram
Recruitment	14a	Dates defining the periods of recruitment and follow-up	Section: Methods, subsection participants. subparagraph : 1. Additionally in S2 Trial Flow Diagram and in Methods, subsection Procedures following treatment administration , paragraph 1-3
	14b	Why the trial ended or was stopped	Not applicable
Baseline data	15	A table showing baseline demographic and clinical characteristics for each group	Table 1

Numbers analysed	16	For each group, number of participants (denominator) included in each analysis and whether the analysis was by original assigned groups	Section: Methods, subsection Analysis sets in paragraph: 1-3
Outcomes and estimation	17a	For each primary and secondary outcome, results for each group, and the estimated effect size and its precision (such as 95% confidence interval)	Table 2
	17b	For binary outcomes, presentation of both absolute and relative effect sizes is recommended	Table 2 and in S4 Results
Ancillary analyses	18	Results of any other analyses performed, including subgroup analyses and adjusted analyses, distinguishing pre-specified from exploratory	S4 Results S5 Results
Harms	19	All important harms or unintended effects in each group (for specific guidance see CONSORT for harms)	Section: Results Paragraph: 8 In addition to the Abstract
**Discussion**			
Limitations	20	Trial limitations, addressing sources of potential bias, imprecision, and, if relevant, multiplicity of analyses	Section: Discussion Paragraph: 13-14 Abstract
Generalisability	21	Generalisability (external validity, applicability) of the trial findings	Section: Discussion in the second to last paragraph. It is also described in the Abstract
Interpretation	22	Interpretation consistent with results, balancing benefits and harms, and considering other relevant evidence	Section: Discussion

			Paragraph: 1,2,3 in particular in addition to 6, 7, 10,11,13, 14-15.
**Other information**			
Registration	23	Registration number and name of trial registry	Section: Methods, subsection Study design Subsection paragraph: 2
Protocol	24	Where the full trial protocol can be accessed, if available	S1 Study Protocol and in published paper DOI: 10.1371/journal.pone.0276613
Funding	25	Sources of funding and other support (such as supply of drugs), role of funders	Role of the founding source is reported in separate document upon addition to PLOS MED

*We strongly recommend reading this statement in conjunction with the CONSORT 2010 Explanation and Elaboration for important clarifications on all the items. If relevant, we also recommend reading CONSORT extensions for cluster randomised trials, non-inferiority and equivalence trials, non-pharmacological treatments, herbal interventions, and pragmatic trials. Additional extensions are forthcoming: for those and for up to date references relevant to this checklist, see www.consort-statement.org.
