# Supplementary material for: The effect of intravenous hyoscine butylbromide on slow progress in labor (BUSCLAB): A double-blind randomized placebo-controlled trial
Source: PLoS Med. 2024 Mar 28;21(3):e1004352. doi: 10.1371/journal.pmed.1004352 (PMC11008832; doi:10.1371/journal.pmed.1004352)
Supplement: S1 Text — (PDF) [file pmed.1004352.s002.pdf]

BUSCLAB - A DOUBLE BLIND RANDOMIZED PLACEBO-CONTROLLED TRIAL INVESTIGATING THE EFFECT OF INTRAVENOUS BUTYLSCOPOLAMINE BROMIDE TO PREVENT SLOW PROGRESS IN LABOR

Protocol Identification Number: BUSCLAB001

EudraCT Number: 2018-002338-19

SPONSOR:

Oslo University Hospital

Head of Department

Aase Pay

Sognsvannsveien 20, 0424 Oslo, Norway

Tel : +47 23070000

E-mail: uxlely@ous-hf.no

**PROTOCOL VERSION NO. 14 - 26.05.21**

CONTACT DETAILS

**Sponsor:**	**Aase Pay, Head of Department**<br>Oslo University Hospital<br>0424 Oslo<br>Norway<br>Tel : +47 23070000<br>E-mail: uxlely@ous-hf.no
**Principal investigator:**	**Trond Melbye Michelsen, MD PhD**<br>Oslo University Hospital Rikshospitalet<br>Tel : +47 23070000<br>E-mail: trmi1@ous-hf.no
**Participating Department:**	**Section of Obstetrics Rikshospitalet, Department of Obstetrics, Division of Obstetrics and Gynecology, Oslo University Hospital**<br>Sognsvannsveien 20, 0424 Oslo, Norway<br>Tel : +47 23070000<br>E-mail: uxlely@ous-hf.no
**Monitor:**	**Clinical Trial Unit**<br>Oslo University Hospital<br>0424 Oslo<br>Norway<br>Tel : +47 23070000<br>Email: oushfpbkkf@ous-hf.no

SIGNATURE PAGE

Title	BUSCLAB - a double blind randomized PLACEBO-controlled trial investigating the effect of intravenous Butylscopolamine bromide to prevent slow progress in labor
Protocol ID no:	BUSCLAB.
EudraCT no:	2018-002338-19

I hereby declare that I will conduct the study in compliance with the Protocol, ICH GCP and the applicable regulatory requirements:

Name	Title	Role	Signature	Date
Trond M. Michelsen	Professor/Attending Physician	Principal Investigator	Trond M. Michelsen	26/5 2021
Lise Gaudernack	Midwife	PhD-student	Lise Gaudernack	26/5-21
Mirjam Lukasse	Professor	Main supervisor	Mirjam Lukasse	27.5.21
Nina Gunnes	Researcher	Biostatistician	Nina Gunnes	07.06.2021
Leiv Arne Rosseland	Professor	Project Coworker	[illegible]	14/6-2021
Aase Pay	Head of Department	Sponsor's representative	Aase Pay	14/6-21
Angeline Einarsen	Attending Physician	PhD-student	Angeline S. Einarsen	26.05.21

PROTOCOL SYNOPSIS

BUSCLAB - A double blind randomized placebo-controlled trial investigating the effect of intravenous Butylscopolamine bromide to prevent slow progress in labor

Sponsor	Oslo University Hospital
Phase and study type	Interventional phase 3 study
Investigational Medical Product (IMP):	Butylscopolamine Bromide vs Placebo
Centers:	Oslo University Hospital Rikshopsitalet
Study Period:	Estimated date of first patient enrolled: June 1 2019<br>Anticipated recruitment period: 24 months<br>Estimated date of last patient completed: May 31 2021
Treatment Duration:	A single dose of Investigational Medical Product (IMP)
Follow-up:	1 month
Objectives	To study the effect of Butylscopolamine Bromide on duration of the active phase of first stage of labor in first time mothers who cross the alert-line for labor dystocia, according to the WHO partograph.
Endpoints:	**Primary endpoint:**<br>Duration of labor from the time when the participant was given IMP to delivery<br>**Secondary Endpoints:**

Endpoints regarding labor:

Duration from when the participant was given IMP, to 10 cm dilatation (time to event variable).

Mean cervical dilatation rate, calculated as mean cervical dilatation from IMP is given to 10 cm (continuous variable)

Duration of labor from the onset of active labor (at least 3 cm dilatation) to delivery (time to event variable)

Mode of delivery:

1. Spontaneous vaginal delivery vs operative delivery (vacuum, forceps or cesarean delivery) (dichotomous variable)
2. Vaginal delivery vs cesarean delivery (dichotomous variable)
3. Spontaneous vaginal delivery, vacuum delivery, forceps delivery, or emergency cesarean delivery (nominal variables)

Amount of oxytocin given, measured

1. As total time with treatment (continuous variable)
2. As International Units (IU). The midwife measures mL of oxytocin solution given during labor. IU are calculated based on standardized concentration of the infusion solution [10 IU oxytocin in 1000 mL NaCl solution] (continuous variable).

Pain scores using a Visual Analogue Scale at baseline and 30 minutes after administration of IMP (continuous variable)

Postpartum hemorrhage (continuous variable)

Urinary retention, defined as need for urinary catheter before the participants leave the delivery ward (categorical variable)

Anal sphincter injury (categorical variable)

Endpoints regarding the infant:

Apgar score at 5 minutes and 10 minutes after delivery (ordinal variables)

pH levels in umbilical vein and artery after delivery (continuous variables)

Admission to the Neonatal Intensive Care Unit (categorical variable)

Postpartum endpoints:

Birth experience measured by the validated questionnaire Child Birth

	Experience Questionnaire (continuous variables for each sub category)
Study Design:	Double-blind placebo-controlled randomized controlled trial
Main Inclusion Criteria:	– ≥18 years<br>– Primiparous women<br>– Spontaneous onset of labor<br>– Active phase of labor<br>– ≥37 weeks of gestation<br>– Vertex position<br>– Crossing the alert line, i.e.: cervical dilatation of less than one cm per hour in the active phase of first stage of labour (cervix dilation ≥3 - <10 cm)<br>– Signed informed consent and expected cooperation of the patients for the treatment and follow up must be obtained and documented according to ICH GCP, and national/local regulations.
Main Exclusion Criteria	– Multiple gestation<br>– Elective cesarean section<br>– Women in labor already receiving oxytocin when crossing the alert line<br>– Fully dilated cervix when crossing the alert line<br>– Preeclampsia defined as blood pressure ≥140/90 and proteinuria (+1 or more on a urine dipstick on more than one occasion) with debut after 20 weeks of pregnancy<br>– Known intestinal stenosis, ileus or megacolon<br>– Persisting maternal tachycardia (heart rate >130 beats per minute)<br>– Known maternal myasthenia gravis<br>– Persisting fetal tachycardia (fetal heart rate baseline >170 beats per minute)<br>– Hypersensitivity to any of the ingredients in 'IMP or placebo (butylscopolamine bromide or sodium chloride).<br>– Women with heart disease who are under surveillance with heart rate monitoring during labor<br>– Known fetal heart disease<br>– Untreated glaucoma
Sample Size:	250 patients

Efficacy Assessments:	Time from start of intervention to delivery. Mode of delivery. Dose and time of oxytocin treatment needed.
Safety Assessments:	Fetal heart rate. Maternal heart rate.
Other Assessments:	Pain during labor. Experience of labor and birth.

TABLE OF CONTENTS

CONTACT DETAILS 2

SIGNATURE PAGE 3

PROTOCOL SYNOPSIS 4

TABLE OF CONTENTS 8

LIST OF ABBREVIATIONS AND DEFINITIONS OF TERMS 11

1 INTRODUCTION 12

1.1 Background – Labor arrest 12

1.2 Background - Therapeutic Information 12

1.3 Pre-Clinical & Clinical Experience with Investigational Medicinal Product (IMP) 13

1.4 Rationale for the Study and Purpose 13

1.5 Primary Endpoint 15

1.6 Exploratory endpoints 16

2 OVERALL STUDY DESIGN 17

3 STUDY POPULATION 18

3.1 Selection of Study Population 18

3.2 Number of Patients 18

3.3 Inclusion Criteria 18

3.4 Exclusion Criteria 18

4 TREATMENT 20

4.1 Drug Identity, Supply and Storage 20

4.2 Dosage and Drug Administration 20

4.3 Duration of Therapy 21

4.4 Schedule Modifications 21

4.5 Concomitant Medication 21

4.6 Participant Compliance 22

4.7 Drug Accountability 22

4.8 Drug Labeling 22

4.9 Participant Numbering 22

5 STUDY PROCEDURES 23

5.1 Flow Chart 23

5.2 By Visit 24

5.2.1 Before Treatment Starts 25

5.2.2 During Treatment 25

5.2.3 End of Treatment and/or End of Study Visit (as applicable) 25

5.2.4 Withdrawal Visit 26
5.2.5 After End of Treatment (Follow-up) 26
5.3 Criteria for Participant or Treatment Discontinuation 26
5.4 Procedures for Discontinuation 26
5.4.1 Patient Discontinuation 26
5.4.2 Treatment Discontinuation 26
5.4.3 Trial Discontinuation 26
6 ASSESSMENTS 28
6.1 Assessment of Efficacy Response 28
6.2 Safety and Tolerability Assessments 28
6.3 Other Assessments 29
7 SAFETY MONITORING AND REPORTING 30
7.1 Definitions 30
7.1.1 Adverse Event (AE) 30
7.1.2 Serious Adverse Event (SAE) 30
7.1.3 Suspected Unexpected Serious Adverse Reaction (SUSAR) 31
7.2 Expected Adverse Events 31
7.3 Time Period for Reporting AE and SAE 31
7.4 Recording of Adverse Events 31
7.5 Reporting Procedure 32
7.5.1 AEs and SAEs 32
7.5.2 SUSARs 32
7.5.3 Annual Safety Report 33
7.5.4 Clinical Study Report 33
7.6 Procedures in Case of Emergency 33
7.7 Data Monitoring Committee (DMC) 33
8 DATA MANAGEMENT AND MONITORING 34
8.1 Case Report Forms 34
For Electronic Case Report Forms (eCRFs) 34
8.2 Source Data 34
8.3 Study Monitoring 35
8.4 Confidentiality 35
8.5 Database management 35
9 STATISTICAL METHODS AND DATA ANALYSIS 37
9.1 Sample Size calculation 37

9.2 Randomization 38
9.2.1 Allocation- sequence generation 38
9.2.2 Allocation- procedure to randomize a patient 38
9.2.3 Blinding and emergency unblinding 39
9.3 Population for Analysis 39
9.4 Planned analyses 39
9.5 Statistical Analysis 40
9.5.1 Primary analysis 40
9.5.2 Secondary analyses 41
9.5.3 Background and Demographic Characteristics 41
9.5.4 Exploratory Analysis 41
9.5.5 Safety analyses 41
9.5.6 Other analyses (e.g. health economics, patient reported outcomes etc.) 41
9.5.7 Descriptive statistics 41
10 STUDY MANAGEMENT 42
10.1 Investigator Delegation Procedure 42
10.2 Protocol Adherence 42
10.3 Study Amendments 42
10.4 Audit and Inspections 42
11 ETHICAL AND REGULATORY REQUIREMENTS 43
11.1 Ethics Committee Approval 43
11.2 Other Regulatory Approvals 43
11.3 Informed Consent Procedure 43
11.4 Participant Identification 43
12 TRIAL SPONSORSHIP AND FINANCING 43
13 TRIAL INSURANCE 44
14 PUBLICATION POLICY 44
15 REFERENCES 45

LIST OF ABBREVIATIONS AND DEFINITIONS OF TERMS

Abbreviation or special term	Explanation
AE	Adverse Event
CRF	Case Report Form (electronic/paper)
CSA	Clinical Study Agreement
DAE	Discontinuation due to Adverse Event
EC	Ethics Committee, synonymous to Institutional Review Board (IRB) and Independent Ethics Committee (IEC)
GCP	Good Clinical Practice
IB	Investigator's Brochure
SmPC	Summary of Product Characteristics
ICF	Informed Consent Form
ICH	International Conference on Harmonization
IMP	Investigational Medicinal Product (Butylscopolamine Bromide or Placebo)
SAE	Serious Adverse Event
SOP	Standard Operating Procedure

1 INTRODUCTION

1.1 Background – Labor arrest

Prolonged labor is common, especially among first time mothers, and it is associated with adverse outcomes such as a negative birth experience, operative delivery, chorioamnionitis and admission to NICU (1-4).

1.2 Background - Therapeutic Information

When progress in labor is too slow, augmentation with oxytocin is commonly used to increase contractions (5). However, oxytocin has side effects. Administration of the drug, commonly by continuous intravenously infusion, is usually accompanied by continuous fetal heart monitoring and frequently by epidural analgesia, all decreasing a woman's ability to move. Augmentation with oxytocin is associated with an increased risk of fetal asphyxia (6-8), operative delivery and a negative birth experience (8-10). Oxytocin has been described as the drug most commonly related to preventable adverse events during labor and has a very unpredictable therapeutic index (11). A significant association has been shown between augmentation with oxytocin in first labor and anal sphincter injuries (12), post-partum urinary retention (13), postpartum hemorrhage (14) and delayed initiation of breastfeeding (15). Given that there is uncertainty regarding the efficacy of oxytocin, and that the use of oxytocin has considerable side effects, there is a need to evaluate alternative or adjuvant treatments for slow progress in the first stage of spontaneous labor.

Studies of the prevalence of augmentation with oxytocin report a high and increasing use of this drug, especially among first time mothers (9, 16, 17). A study of all Norwegian delivery units found a prevalence of oxytocin augmentation in primiparous women in spontaneous labor at term, with a single fetus in cephalic presentation (Robson group 1) from 44 to 48% in the years 2000-2011 (18). Data from Oslo University Hospital, Rikshospitalet, 2014-2015, show that 54% of women in Robson group 1 received oxytocin for augmentation of labor ((19)).

Antispasmodics can also be used in order to shorten labor, and thus contribute to the use of oxytocin being reduced. Antispasmodics are drugs that relieve spasms of smooth muscle tissue and have either musculotropic or neurotropic effects. The uterine cervix is composed of connective tissue and smooth muscle, and innervated by parasympathetic nerve fibers. Smooth muscle constitutes about 15% of the cervix (20), and is mainly found just below the internal os (21). Musculotropic antispasmodics directly relax smooth muscles. They are phosphodiesterase type IV inhibitors, structurally related to papaverine, have mild Calcium (Ca)-channel blocking effects, no anticholinergic effects and act directly on smooth muscle cells, inhibiting spasm (21). Neurotropic antispasmodics break the connection between the parasympathetic nerve and the smooth muscle. They are parasympatholytics acting as antagonists of acetylcholine at muscarinic receptors, thus inhibiting muscle spasm. Antispasmodics are commonly administered during labor in both developing and developed countries, although there is a paucity of scientific reports validating this (22). Butylscopolamine bromide (Buscopan) is an antispasmodic drug with a rapid onset <20 minutes (23).

1.3 Pre-Clinical & Clinical Experience with Investigational Medicinal Product (IMP)

Some side effects are reported for butylscopolamine; patients sometimes experience tachycardia and dryness of the mouth. Information provided by the pharmaceutical industry mentions that if given near delivery the fetal heart rate might be influenced (24) The possible influence on the fetal heart rate is likely caused by the temporary tachycardia experienced by the mother. However, none of the 17 studies included in the Cochrane review report an increase in birth asphyxia, nor an increase in required resuscitation or admission to the neonatal care unit. RELIS reports that central effects of butylscopolamine bromide are rare because the drug to a small extent crosses the blood-brain barrier and they state that the drug only to a small extent crosses the placenta, and therefore can be used in pregnancy (25)

The Cochrane Review from 2013 included 17 RCTs on the use of spasmolytics and duration of labor (22). Following this Cochrane report, another study (26) which included 382 Turkish women randomized to 1ml (20mg) butylscopolamine bromide or 1ml of saline in active labor has been published. This study found a reduction in duration of active labor for first time mothers of 57 minutes when treated with butylscopolamine bromide as soon as active stage of labor started.

In the Cochrane review, two studies have been conducted in Turkey, nine in India, three in Iran, two in Saudi Arabia and one each in Jamaica, Kenya, Italy, Nepal and the United States. All studies have used spasmolytics as routine treatment, in most cases as part of active management of labor. Thus, there are no studies that have used spasmolytics on slow progress in childbirth and there are no studies from the Western World that have used butylscopolamine bromide.

Overall, the studies find significant reduction in duration of first stage of labor and shortening of total duration of labor when using spasmolytics compared to placebo (opening phase 74 minutes shorter [13 studies, 1995 women], total birth length 85 minutes shorter [seven studies, 797 women]), but no effect on duration of second or third stages of birth. The review concludes: "There is insufficient evidence to make any conclusions regarding the safety of these drugs for both mother and baby. Large, rigorous randomized controlled trials are needed to evaluate the effect of antispasmodics on prolonged labor and to evaluate their effect on labor in a context of expectant management of labor" (22). Two large studies on first time mothers' labor duration find a mean dilatation of active phase of labor of 1.2 cm (27) and 1.4 cm per hour (28). In the present study we define women in risk of having a prolonged labor as those with a dilatation rate less than 1 cm per hour (crossing the alert line of the partogram) in the active phase of labor.

1.4 Rationale for the Study and Purpose

In summary, slow progress in labor is a common condition, especially in primiparous women, and is associated with several unwanted outcomes. Oxytocin is widely used to treat slow progress in labor, but has serious potential side effects. Previous studies demonstrate that spasmolytics may shorten duration of labor, but studies on the effect of spasmolytics as a prevention of slow progress in labor are lacking. The studies conducted to date seem to have a reassuring safety profile regarding undesirable maternal and neonatal events. Additional treatments to reduce duration of labor are highly warranted, and this study is powered to clarify whether butylscopolamine bromide has a role in first time labor.

If the IMP has the anticipated effect, labor will be shortened for women who receive the active drug. If the IMP has the anticipated effect, use of oxytocin may be lower in the active treatment group, and oxytocin side effects may be reduced.

1.5 Benefits and risks

RELIS states that Buscopan to a little degree passes the placenta, and that there is little reason to believe that Buscopan given to the mother affects the fetus (24).

However, Legemiddelhåndboka says that Buscopan given to the mother near term can give fetal tachycardia (29). The reason for fetal tachycardia is believed to be maternal tachycardia due to anticholinergic effects of Buscopan. These anticholinergic side effects are described as mild and transient.

Buscopan may be transferred to breast milk. Full clearance of the drug is believed to take 24 hours. However, the first 24 hours after birth the milk production is limited.

Given that Buscopan has the expected effect, the need for oxytocin to augment contraction may be reduced. Oxytocin, although widely used in labor, has been described as the drug most commonly related to preventable adverse events during labor and has a very unpredictable therapeutic index(11)). Reduced amounts of oxytocin may reduce unwanted side effects.

Side effects of **oxytocin**, as reported in Legemiddelhåndboken and Felleskatalogen include (29, 30):

Maternal: Common (≥1/100 to <1/10): Gastrointenstinal: Nausea, vomiting. Cardiovascular: Tachycardia, bradycardia. Neurological: Headache. Less common (≥1/1000 to <1/100): Cardiovascular: Arrhythmia. Rare (≥1/10000 to <1/1000): Skin: Rash. Immune system: Anaphylactic reaction with dyspnea, hypotension or shock. Unknown frequency: Blood/lymphatic system: Disseminated Intravascular Coagulation (DIC). Cardiovascular: Ischemia, prolonged QT interval. Airways: Pulmonary edema. Metabolism: Water intoxication, maternal hyponatremia. Pregnancy: Uterine hyper stimulation, tetanic contraction, uterine rupture. Urinary retention.

Fetal/neonatal: Unknown frequency: Metabolism: Neonatal hyponatremia. Pregnancy: Fetal distress, fetal asphyxia and death.

Side effects of Buscopan, as described in Legemiddelhåndboken and Felleskatalogen:

Maternal: Common (≥1/100 to <1/10): Gastrointestinal: Dryness in the mouth. Cardiology: Tachycardia, dizziness. Ophtalmology: Accomodation disturbance. Unknown frequency: Cardiology: Hypotension, flushing. Skin: Dyshidrosis. Immune system: Anaphylactic shock, anaphylactic reactions, dyspnea, skin reactions including urticarial, rash, erythema and itching. Other forms of hypersensitivity. Kidney: Urinary retention. Ophtalmology: Mydriasis, increased intraocular pressure. Rare cases with serious skin reactions like Steven-Johnsons syndrome have been reported.

According to WHO recommendations for augmentation of labor based on the Cochrane review from 2013 (22),(31), there are 5 RCTs comparing Buscopan with placebo including altogether 845 newborns with no differences in transfer to the Neonatal Intensive Care Unit. One RCT with 100 fetuses did not show any differences in prevalence of fetal distress. One RCT with 130 fetuses did not show any difference in fetal bradycardia, and two RCTs with 230 fetuses did not show any difference in fetal tachycardia. None of the 17 RCTs in the Cochrane review reported any side effects related to Buscopan (22).

Women who are allocated to the control group will contribute to answer an important medical question for future laboring women and their children. They will need an IV access, which could be considered a down side. However, it is routine procedure in the hospital to establish an IV line

when there is labor dystocia. The IV access may be an advantage if they women augmentation with oxytocin or if something unexpected happens.

Women who are allocated to the intervention group may experience a shorter labor if the IMP has the presumed effect. In that case, they will receive less oxytocin, and experience fewer oxytocin side effects. Based on previous studies, the reduction in the active phase of labor will be approximately one hour. This reduction will take place in the first stage (opening phase), and not the second stage of labor (expulsion phase).

Based on data from our department, 72% of primiparous women who crossed the alert line (the inclusion criterion of this study) of the partogram received augmentation with oxytocin with an average duration of 214 minutes. Of the women who crossed the alert line, 15% delivered by cesarean section and 25% had an operative vaginal delivery, compared to 2% and 16% among women with normal progress in labor, respectively.

Based on our evaluation, the anticipated benefits for the individual trial subject and the society from the current study seem to outweigh the foreseeable risks and inconveniences.

1.6 STUDY OBJECTIVES and related endpoints

The study is designed to estimate the effect of intravenously administered Butylscopolamine bromide on the duration of labor. Outcomes also include labor pain, use of oxytocin augmentation, mode of delivery, and a validated questionnaire on birth experience one month after delivery.

1.7 Primary Endpoint

Primary endpoint:

Duration of labor from the time when the participant was given IMP to delivery

Secondary Endpoints:

Endpoints regarding labor:

Duration from when the participant was given IMP, to 10 cm dilatation (time to event variable).

Mean cervical dilatation rate, calculated as mean cervical dilatation from IMP is given to 10 cm (continuous variable)

Duration of labor from the onset of active labor (at least 3 cm dilatation) to delivery (time to event variable)

Mode of delivery:

1. Spontaneous vaginal delivery vs operative delivery (vacuum, forceps or cesarean delivery) (dichotomous variable)
2. Vaginal delivery vs cesarean delivery (dichotomous variable)
3. Spontaneous vaginal delivery, vacuum delivery, forceps delivery, or emergency cesarean delivery (nominal variables)

Amount of oxytocin given, measured

1. As total time with treatment (continuous variable)
2. As International Units (IU). The midwife measures mL of oxytocin solution given

during labor. IU are calculated based on standardized concentration of the infusion solution [10 IU oxytocin in 1000 mL NaCl solution] (continuous variable).

Pain scores using a Visual Analogue Scale at baseline and 30 minutes after administration of each dose of IMP (continuous variable)

Postpartum hemorrhage (continuous variable)

Urinary retention, defined as need for urinary catheter before the participants leave the delivery ward (categorical variable)

Anal sphincter injury (categorical variable)

Endpoints regarding the infant:

Apgar score at 5 minutes and 10 minutes after delivery (ordinal variables)

pH levels in umbilical vein and artery after delivery (continuous variables)

Admission to the Neonatal Intensive Care Unit (categorical variable)

Postpartum endpoints:

Birth experience measured by the validated questionnaire Child Birth Experience Questionnaire (continuous variables for each sub category)

1.8 Exploratory endpoints

Cervical dilatation rate, calculated as a function of time (functional data)

Indication for operative delivery:

1. Labor dystocia (defined in Stimulering av rier/Langsom framgang i fødsel [eHåndbok]),
2. In first stage: If the action line is crossed, amniotomy should be performed (The Partogram has an alert and an action line. The alert line marks 1 cm per hour and the action line is 4 hours shifted to the right of this). After amniotomy, wait at least 1 hour before augmenting with oxytocin stimulation. If progress in birth await oxytocin. If the water is already broken when slow progress occurs, oxytocin infusion should be started.
3. Fetal distress (non-reassuring fetal heart rate tracing as defined in Fosterovervåkning under fødsel [eHåndbok])

Avvikende CTG	100-110 slag/min<br><br>150-170 slag/min<br><br>-Kort bradycardiepisode:<br><100 slag/min for > 3 min<br><80 slag/min > 2 min	> 25 slag/min<br>(saltatorisk mønster)<br><br>< 5 slag/min > 40 min	-Ukompliserte variable deselerasjoner<br>(slagtap > 60)

En kombinasjon av 2 eller flere avvikende faktorer gir et patologisk CTG			
Patologisk CTG	>170 slag/min<br><br>-Bestående bradycardi:<br><100 slag/min i > 10 min<br><80 slag/min i > 3 min uten stigende tendens	< 5 slag/min i > 60 min<br>Sinusoidalt mønster	-Kompliserte variable deselerasjoner:<br>varer > 60 sek.<br><br>-Tilbakevendende sene uniforme deselerasjoner
Preterminalt CTG	Fravær av variabilitet < 2 slag/min eller reaktivitet med eller uten deselerasjoner eller bradycardi		

4. Other indications

Maternal heart rate: Continuous registration at least 30 minutes from IMP is given.

Fetal heart rate: Continuous registration at least 30 minutes from IMP is given.

2 OVERALL STUDY DESIGN

The study is a double-blind, randomized placebo-controlled trial

Study Period	Estimated date of first patient enrolled: June 1 2019
	Anticipated recruitment period: 24 months
	Estimated date of last patient completed: May 31 2021
Treatment Duration:	One dose of Investigational Medical Product (IMP)
Follow-up:	1 month

3 STUDY POPULATION

3.1 Selection of Study Population

Academic hospital in Norway. Primiparous women who are giving birth at Department of Obstetrics Rikshospitalet, Oslo University Hospital are invited to participate.

The women will be invited approximately six weeks prior to their due date (gestational week 30), and will receive a mailed invitation letter. In the letter, they will be given opportunity to actively opt out of inclusion. At this time, women may give informed consent to participate, or they can be included when they arrive at the delivery ward. All women will have the opportunity to talk to a midwife or doctor before they give written informed consent, and contact information will be provided in the invitation. The Principal Investigator will sign the consent form.

Women who have received and understood information about the study and are willing to participate prior to start of labor may be included and sign the informed consent form when they arrive at the hospital to give birth.

3.2 Number of Patients

A maximum number of 250 patients will be included in this trial.

3.3 Inclusion Criteria

All of the following conditions must apply to the prospective patient at screening prior to receiving study agent:

- ≥18 years
- Primiparous women
- Spontaneous onset of labor
- Active phase of labor
- ≥37 weeks of gestation
- Vertex position
- Crossing the alert line, i.e.: cervical dilatation of less than one cm per hour in the active phase of first stage of labor (cervix dilation ≥3 - <10 cm)

3.4 Exclusion Criteria

Patients will be excluded from the study if they meet any of the following criteria:

- Multiple gestation
- Elective cesarean section

- Women in labor receiving oxytocin before start of active labor (cervix: 3 cm)
- Fully dilated cervix when crossing the alert line
- Preeclampsia defined as blood pressure ≥140/90 and proteinuria (+1 or more on a urine dipstick on more than one occasion) with debut after 20 weeks of pregnancy
- Previous major uterine surgery
- Known intestinal stenosis, ileus or megacolon
- Persisting maternal tachycardia (heart rate >130 beats per minute)
- Known maternal myasthenia gravis
- Persisting fetal tachycardia (fetal heart rate baseline >170 beats per minute)
- Hypersensitivity to any of the ingredients in 'IMP or placebo (butylscopolamine bromide or sodium chloride).
- Women with heart disease who are under surveillance with heart rate monitoring during labor
- Known fetal heart disease
- Untreated glaucoma

4 TREATMENT

For this study Butylscopolamine Bromide and placebo (sodium chloride) are defined as the Investigational Medicinal Product (IMP).

The intervention group
Women randomised to the intervention group will receive 20 mg Butylscopolamine bromide (20mg/ml) intravenously when they cross the alert line of the partogram, In addition they will be given standard care, i.e. augmentation with oxytocin as per ward procedure. If there is indication for Buscopan and oxytocin at the same time, both drugs can be given simultaneously.

The control group
Women randomised to the control group will receive saline solution 1 ml intravenously when they cross the alert line of the partogram, In addition they will be given standard care, i.e. augmentation with oxytocin as per ward procedure.

4.1 Drug Identity, Supply and Storage

Drug: Butylscopolamine Bromide (Buscopan), Boehringer Ingelheim, 20 mg/mL. 1 mL contains Butylscopolamine Bromide 20 mg, sodium chloride 6 mg and water.

Placebo: Sodium chloride 1 mL (Natriumklorid "Fresenius Kabi" oppløsningsvæske til parenteral bruk 9mg/mL).

The placebo and drug preparations will be visually identical – both are colorless fluids.

We refer to the storage and preparation instructions on the package leaflet.

4.2 Dosage and Drug Administration

IMP will be given, as soon as possible after it is noticed that a woman has crossed the alert line, as one dose of 1 mL (butylscopolamine bromide 20 mg/mL or placebo) intravenously.

To our knowledge, no dose finding studies have been performed on laboring women. The RCTs that have been performed have mostly used 20 mg iv. There is one unpublished study that used 40 mg iv, and one RCT that used 40 mg rectal dose. There is not enough data to perform complete comparisons between 20 mg iv and 40 mg iv, but so far 20 mg iv seems to be as effective as 40 mg iv when the measure of effect is duration of active phase of labor and the Buscopan is administered routinely in all laboring women (please see table).

Author (ref)	*Dose (mg) /adm*	*Points Cochrane **	*Country*	*Journal*	*N/parity*	*Time difference (minutes)*
Al Qahanti (32)	*40 mg im*	*5*	*Saudi Arabia*	*Ther Clin Risk Manag.*	*97/ para 0*	*50*
Makvandi (33)	*20 supp*	*4*	*Iran*	*Croat Med Journ*	*130/ para 0*	*89*

Mukaindo	*40 mg iv*	*7*	*Kenya*	*unpublished*		
Samuels (34)	*20 iv*	*7*	*Jamaica*	*BJOG*	*129/mix*	*72*
Sekhavat (35)	*20 iv*	*3*	*Iran*	*African Health Science*	*188/multipara*	*74*
Kirim (26)	*20 iv*	*Published after Cochrane review*	*Turkey*	*Journal of Maternal, Fetal and Neon. Med.*	*387/mix*	*57 minutes*

The study treatment will be administered to the woman by authorized site personnel only.

IMP must be given as soon as possible after randomization. If IMP is given more than 45 minutes after randomization, the midwife must check the cervical dilatation again before IMP is given.

The intravenous injection should be performed 'slowly' (in rare cases a marked drop in blood pressure and even shock may be produced by Buscopan).

4.3 Duration of Therapy

Patients will receive one dose of IMP, 1 mL intravenously. All other treatment is given according to ward procedures.

4.4 Schedule Modifications

4.5 Concomitant Medication

All concomitant medication (incl. vitamins, herbal preparation and other "over-the-counter" drugs) used by the patient will be recorded in the patient's file and CRF. Several drugs may enhance the anticholinergic effect of butylscopolamine and should not be administered during labor. These include:

Antihistamines

Tricyclic and tetracyclic antidepressants

Antipsychotic drugs

Other anticholinergic drugs (tiatropium, ipratropium, atropine)

Metoclopramide

Beta-adrenergic medications may give tachycardia

4.6 Participant Compliance

Compliance with medications is recorded by the midwife in charge. Given that the IMP is administered intravenously, compliance is not expected to be a problem.

4.7 Drug Accountability

The responsible site personnel will confirm receipt of study drug and will use the study drug only within the framework of this clinical study and in accordance with this protocol. Receipt, distribution, return, and destruction (if any) of the study drug will be properly documented on a list in the locked room, and in the medical record.

4.8 Drug Labeling

The IMPs will have standard labeling from the manufacturer including package insert until it is prepared by the unblinded study personnel.

The IMPs will be ordered from the hospital pharmacy's general drug supply according to the same procedures as for any patient who is given treatment with one of these drugs. The unblinded midwife who prepares the solution for intravenous injection will record the batch number and expiry date from the original drug package, write the information on the randomization sheet, and put it back into the envelopes used for randomization. The midwife thereafter closes and seals the envelopes. The envelopes are kept in the locked room in case of the need for unblinding. The batch number and expiry date will be recorded next to the participant number, which will be used for the respective solution for intravenous injection and recorded on the label used for the final solution for intravenous injection.
The solution container will be labeled with the following information:

- The name of the study
- The name of the Principal Investigator
- Department of Obstetrics
- "Buscopan or saline for iv injection"
- Participant number

4.9 Participant Numbering

Each woman is identified in the study by a unique participant number that is assigned when the woman signs the Informed Consent Form. Once assigned the participant number cannot be reused for any other woman.

5 STUDY PROCEDURES

5.1 Flow Chart

Table 1. Trial flow chart

	Screening Period	Delivery			End of maternity ward stay		Follow-up evaluation
Time		Baseline (≥3cm and < 10 cm dilatation) Crossing the alert line	IMP is given	30 min after IMP is given	Day of delivery	Day of discharge	1 month postpartu m
Informed consent	X						
Inclusion/exclusion Evaluation	X						
Medical History	X						
Concomitant Medication	X	X					
[a]Physical Examination fetus (CTG)		X	X	X			
[b]Vital signs mother		X	X		X	X	
Cervical dilatation		X					
Pain measurement (VAS)			X	X			
Treatment administration			X				
Adverse event, mother			X	X	X	X	
Adverse event, newborn/fetus			X	X	X	X	
[c]Physical Examination newborn (pediatrician)						1st day post partum	
Questionnaire (Delivery experience)							X

[a]Physical Examination fetus (CTG) includes continuous fetal heart rate tracing.

[b]Vital signs mother include blood pressure and pulse. Maternal height and body weight will be obtained from the pregnancy chart.

[c]Physical Examination newborn will include an examination of general appearance

5.2 By Visit

Informed consent

All participating women will sign an informed consent during pregnancy. They will receive the invitation to participate in the study six weeks before their due date if they are primiparous, planning a vaginal delivery, and are going to give birth at Oslo University Hospital Rikshospitalet. The laboring women and their fetuses will be monitored after inclusion using CTG (cardiotocography) and measurement of the maternal heart rate for 30 minutes after the treatment is initiated. Patient reported side effects (maternal) will be registered in the CRF based on a list of symptoms. Occurrence, degree and duration of side effects will be registered within 30 minutes after the treatment is given. Treatment of side effects will be registered. All women will be asked to assess labor pain before initiation of the treatment, and 30 minutes after initiation of the treatment, as well as to fill in a validated questionnaire on birth experience one month after delivery.

Informed consent must have been given voluntarily by each woman before any study specific procedures are initiated. The following data will be retrieved from the participants' medical charts and pregnancy charts:

Clinical status

Maternal medical history (including disease history and corresponding treatment details), weight, blood pressure and pulse. Fetal cardiotocography (at least 10 minutes if not already recorded).

Concomitant medication

All concomitant medication used by the woman within 7 days of treatment start must be recorded in the CRF. Concomitant medication will also be recorded at randomization

Physical examination fetus

The physical examination of the fetus includes continuous fetal heart monitoring with ST-analysis and will be recorded from the administration of IMP and 30 minutes after the administration, if any abnormal heart rate is discovered, the fetal heart rate will be monitored until delivery.

Physical examination newborn

Umbilical cord pH from the umbilical artery and vein will be obtained immediately after delivery. Pulse oximetry with O2 saturation will be performed 4 hours after delivery. Physical examination of the newborn will be performed on the first or second day postpartum by an experienced pediatrician from the neonatal intensive care unit. All newborns will be observed by trained personnel at the maternity ward throughout the stay.

Physical examination mother

Includes heart rate and blood pressure

Cervical dilatation

Will be examined immediately before randomization (after the woman has crossed the alert line of the partogram), when IMP is given (if there is more than 45 minutes between randomization and IMP). After IMP is given the midwife will examine the cervix according to ward routine, but at least every two hours.

Questionnaire Childbirth experience questionnaire (CEQ) developed and validated in Sweden consists of 22 items in 4 categories: own capacity, professional support, perceived safety and participation. We have added one question concerning request for caesarean section in next pregnancy

Pain measurement

Pain before and after administration of IMP will be self-reported on a visual analogue scale.

Adverse events

Adverse events will be recorded for both mother and fetus after 30 minutes after IMP is given, on the first day after delivery and at discharge from the maternity ward.

Eligibility criteria

All eligibility criteria should be assessed together with relevant baseline parameters prior to study inclusion (inclusion/exclusion criteria), and eligibility should be confirmed in the inclusion chart.

5.2.1 Before Treatment Starts

- Eligibility will be assessed by the inclusion chart
- Baseline is defined as the time point in labor when slow progress is detected. At baseline, vital signs from mother and fetus will be obtained, and we will perform baseline observations regarding medical history
- For the endpoint pain baseline measurements by a VAS scale will be obtained before randomization

5.2.2 During Treatment

All treatment events take place during labor. Eligible women will receive one dose of IMP . Efficacy is assessed by cervical dilation (measured at least every two hours), time to delivery, dose and time of oxytocin treatment needed and mode of delivery. Efficacy regarding pain is assessed by VAS scale measurements 30 minutes after initiation of IMP.

Toxicity is assessed by maternal vital signs (continuous heart rate measurements) and continuous fetal heart rate tracing.

Other treatments for labor arrest will be offered to the participants as per ward procedure. These treatments include artificial rupture of membranes and intravenous oxytocin infusion.

5.2.3 End of Treatment and/or End of Study Visit (as applicable)

5.2.4 The end of trial is defined as when the "one-month post-partum questionnaires" has been received from all participants, but not

A woman can withdraw from the study at any time, and will still receive treatment before and after birth as per ward procedure. Time to delivery, mode of delivery and dose of oxytocin will be recorded, at the discretion of the woman.

5.2.5 After End of Treatment (Follow-up)

Follow-up information will be obtained one month after delivery by a standardized questionnaire measuring experience of labor and birth.

5.3 Criteria for Participant or Treatment Discontinuation

Participants may be discontinued from study treatment. Specific reasons for discontinuing treatment for this study are:

- Safety reason as judged by the Principal Investigator
- Incorrect enrolment i.e., the participant does not meet the required inclusion/exclusion criteria for the study
- Deterioration in the participant's condition which in the opinion of the Principal Investigator warrants study medication discontinuation (to be recorded as an AE or under Investigator Discretion)
- Voluntary discontinuation by the participant who is at any time free to discontinue her participation in the study, without prejudice to further treatment.

5.4 Procedures for Discontinuation

5.4.1 Patient Discontinuation

All patients randomized will be included in the study population.

Patients who are randomized in spite of not fullfilling all of the inclusion criteria or fullfilling one or several exclusion criteria can be replaced. Patients who withdraw from the study after randomization cannot be replaced.

If the patient consents, end of study information will be obtained and recorded.

5.4.2 Treatment Discontinuation

If treatment was discontinued, all study assessments as described in this protocol should be recorded.

5.4.3 Trial Discontinuation

The whole trial may be discontinued at the discretion of the PI or the sponsor in the event of any of the following:

- Occurrence of AEs unknown to date in respect of their nature, severity and duration

- Medical or ethical reasons affecting the continued performance of the trial
- Difficulties in the recruitment of patients

The sponsor and principal investigator(s) will inform all investigators, the relevant Competent Authorities and Ethics Committees of the termination of the trial along with the reasons for such action. If the study is terminated early on grounds of safety, the Competent Authorities and Ethics Committees will be informed within 15 days.

6 ASSESSMENTS

6.1 Assessment of Efficacy Response

The following parameters will be recorded:

Duration of labor from the time when the participant was given IMP to delivery

Duration of labor from the onset of active labor (at least 3 cm dilatation) to delivery

Duration of the active phase of first stage of labor (at least 3 cm dilatation), from the time when it was noticed that the alert-line of the partogram was crossed (between 3 cm and 9 cm dilatation) and the participant was given IMP, to 10 cm dilatation, or emergency cesarean delivery.

Mode of delivery – spontaneous vaginal delivery, vacuum delivery, forceps delivery, emergency cesarean delivery

Indication for operative delivery

Amount of oxytocin given measured as

1. total time with treatment

2. International Units (IU) (the midwife measures mL of oxytocin solution given during labor. IU are calculated based on standardized concentration of the infusion solution [10 IU oxytocin in 1000 mL NaCl solution])

Apgar score at 5 minutes and 10 minutes after delivery

Admission to the Neonatal Intensive Care Unit

Pain scores using a Visual Analogue Scale at baseline and 30 minutes after administration of IMP

Birth experience measured by the validated questionnaire Child Birth Experience Questionnaire

Maternal heart rate – continuous registration at least 30minutes from administration of IMP

Fetal heart rate - continuous registration at least 30minutes from administration of IMP

Urinary retention

Anal sphincter injury

Postpartum hemorrhage

6.2 Safety and Tolerability Assessments

Safety will be monitored by the assessments described below as well as the collection of AEs. Significant findings that are present prior to the signing of informed consent must be included in the

relevant medical history/ current medical condition page of the CRF. For details on AE collection and reporting, refer to Section 7.5.

For the assessment schedule refer to Flow chart in Section 5.

Physical examination will include an examination of general appearance.

Vital signs mother include blood pressure and pulse.

Vital signs fetus include continuous fetal heart rate tracing.

Maternal height and body weight will be obtained from the pregnancy chart.

6.3 Other Assessments

The study participants will complete a questionnaire that examines experience of labor and birth one month after delivery (36).

7 SAFETY MONITORING AND REPORTING

The investigator is responsible for the detection and documentation of events meeting the criteria and definition of an adverse event (AE) or serious adverse event (SAE). Each patient will be instructed to contact the investigator immediately should they manifest any signs or symptoms they perceive as serious.

The methods for collection of safety data are described below.

7.1 Definitions

7.1.1 Adverse Event (AE)

An AE is any untoward medical occurrence in a patient administered a pharmaceutical product and which does not necessarily have a causal relationship with this treatment.

An adverse event (AE) can therefore be any unfavorable and unintended sign (including an abnormal laboratory finding), symptom, or disease temporally associated with the use of a medicinal (investigational) product, whether or not related to the medicinal (investigational) product.

The term AE is used to include both serious and non-serious AEs.

If an abnormal laboratory value/vital sign is associated with clinical signs and symptoms, the sign/symptom should be reported as an AE and the associated laboratory result/vital sign should be considered additional information that must be collected on the relevant CRF.

7.1.2 Serious Adverse Event (SAE)

Any untoward medical occurrence that at any dose:

- Results in death
- Is immediately life-threatening
- Requires in-patient hospitalization or prolongation of existing hospitalization
- Results in persistent or significant disability or incapacity
- Is a congenital abnormality or birth defect
- Is an important medical event that may jeopardize the participant or may require medical intervention to prevent one of the outcomes listed above.

Medical and scientific judgment is to be exercised in deciding on the seriousness of a case. Important medical events may not be immediately life-threatening or result in death or hospitalization, but may jeopardize the participant or may require intervention to prevent one of the listed outcomes in the definitions above. In such situations, or in doubtful cases, the case should be considered as serious. Hospitalization for administrative reason (for observation or social reasons) is allowed at the investigator's discretion and will not qualify as serious unless there is an associated adverse event warranting hospitalization.

A pre-planned hospitalization admission (i.e., elective or scheduled surgery arranged prior to the start of treatment) for pre-existing condition is not considered to be a serious adverse event.

7.1.3 Suspected Unexpected Serious Adverse Reaction (SUSAR)

Suspected Unexpected Serious Adverse Reaction: SAE (see section 8.1.2) that is unexpected as defined in section 8.2 and possibly related to the investigational medicinal product(s).

7.2 Expected Adverse Events

Expected AEs are: Maternal flushing, maternal tachycardia, fetal tachycardia. Other AEs are described in the SmPC.

7.3 Time Period for Reporting AE and SAE

For each patient the standard time period for collecting and recording AE and SAEs will begin at start of study treatment and will continue throughout the hospital stay.

During the course of the study all AEs and SAEs will be proactively followed up for each patient; events should be followed up to resolution, unless the event is considered by the investigator to be unlikely to resolve due to the underlying disease. Every effort should be made to obtain a resolution for all events, even if the events continue after discontinuation/study completion.

7.4 Recording of Adverse Events

If the patient has experienced adverse event(s), the investigator will record the following information in the CRF:

- The nature of the event(s) will be described by the investigator in precise standard medical terminology (i.e. not necessarily the exact words used by the patient).
- The duration of the event will be described in terms of event onset date and event ended data.
- The intensity of the adverse event will be described as mild, moderate and severe.
    - Mild; asymptomatic or mild symptoms; clinical or diagnostic observations only; intervention not indicated.
    - Moderate; minimal, local or noninvasive intervention indicated; limiting age-appropriate instrumental Activities of Daily Living.
    - Severe or medically significant but not immediately life-threatening; hospitalization or prolongation of hospitalization indicated.
- Specifically, mild AEs include dryness of the mouth, transient tachycardia and flushing. Moderate AEs include visual disturbances, urinary retention, and fall in blood pressure and skin reactions. SAEs include dyspnea, anaphylactic reactions, serious skin reactions including Steven-Johnson and anaphylactic shock.
- The Causal relationship of the event to the study medication will be assessed as one of the following:

Unrelated:
There is not a temporal relationship to investigational product administration (too early, or late, or investigational product not taken), or there is a reasonable causal relationship between non-investigational product, concurrent disease, or circumstance and the AE.

Unlikely:
There is a temporal relationship to investigational product administration, but there is not a reasonable causal relationship between the investigational product and the AE.

Possible:
There is reasonable causal relationship between the investigational product and the AE. Dechallenge information is lacking or unclear.

Probable:
There is a reasonable causal relationship between the investigational product and the AE. The event responds to dechallenge. Rechallenge is not required.

Definite:
There is a reasonable causal relationship between the investigational product and the AE.

- Action taken
- The outcome of the adverse event – whether the event is resolved or still ongoing.

7.5 Reporting Procedure

7.5.1 AEs and SAEs

All adverse events and serious adverse events will be recorded in the patient's CRF and according to CTCAE (Common Criteria for Adverse Events).

SAEs must be reported within 24 hours after the site has gained knowledge of the SAE. Every SAE must be documented by the investigator on the SAE pages (to be found as part of the CRF). The Serious Adverse Event Report Form must be completed, signed and sent to the medical officer (Head of Department Aase Pay). The initial report shall promptly be followed by detailed, written reports if necessary. The initial and follow-up reports shall identify the trial participants by unique code numbers assigned to the latter.

The medical officer keeps detailed records of all SAEs reported by the investigators and performs an evaluation with respect to causality and expectedness. Based on, among other, SAE reports the sponsor will evaluate whether the risk/benefit ratio associated with study is changed.

7.5.2 SUSARs

SUSARs will be reported to the Competent Authority according to national regulation. The following timelines should be followed:

The sponsor will ensure that all relevant information about suspected serious unexpected adverse reactions that are fatal or life-threatening is recorded and reported as soon as possible to the

Competent Authority in any case no later than seven (7) days after knowledge by the sponsor of such a case, and that relevant follow-up information is subsequently communicated within an additional eight (8) days.

All other suspected serious unexpected adverse reactions will be reported to the Competent Authority concerned as soon as possible but within a maximum of fifteen (15) days of first knowledge by the sponsor.

In order to avoid unnecessary unblinding of study personnel, an independent person at the Clinical Trial Unit at Oslo University Hospital, will unblind before sending to Competent Authority

SUSARs will be reported using the CIOMS form.

Reference Safety Information: No SARs are expected by the sponsor for the purpose of expedited reporting of SUSARs in this clinical trial, i.e. all adverse events that have a reasonable possibility of a causal relationship with the IMP and are "serious" will be reported as SUSARs.

7.5.3 Annual Safety Report

Once a year throughout the clinical trial, the sponsor will provide the Competent Authority with an annual safety report. The format will comply with national requirements.

7.5.4 Clinical Study Report

The adverse events and serious adverse events occurring during the study will be discussed in the safety evaluation part of the Clinical Study Report.

7.6 Procedures in Case of Emergency

The investigator is responsible for assuring that there are procedures and expertise available to cope with emergencies during the study.

In case of emergency, code break will be possible. Closed envelopes with information about IMP will be kept in a locked room at the maternity ward, as described in 9.2.

If a participant experiences a SAE during treatment, unblinding is permissible. Every effort should be made to contact the medical officer before unblinding, unless the SAE is considered to be life threatening.

7.7 Data Monitoring Committee (DMC)

The study will be supervised by an external monitor appointed by The Clinical Trials Unit at Oslo University Hospital. The monitor will perform reviews every 6 months throughout the study period..

8 DATA MANAGEMENT AND MONITORING

8.1 Case Report Forms

For Electronic Case Report Forms (eCRFs)

The Clinical Data Management System (CDMS) used for the eCRF in this study is Epidata. The eCRF system will be FDA Code of Federal Regulations 21 Part 11 compliant.

The designated investigator staff will enter the data required by the protocol into the eCase report forms (eCRF). The Investigator is responsible for assuring that data entered into the eCRF is complete, accurate, and that entry is performed in a timely manner. The signature of the investigator will attest the accuracy of the data on each eCRF. If any assessments are omitted, the reason for such omissions will be noted on the eCRFs. Corrections, with the reason for the corrections will also be recorded.

After database lock, the investigator will receive a digital copy of the participant data for archiving at the investigational site.

8.2 Source Data

Source data are all information in original records and certified copies of original records of clinical findings, observations, or other activities in a clinical trial necessary for the reconstruction and evaluation of the trial. Source data are contained in source documents (original records or certified copies).

The medical records for each patient should contain information which is important for the patient's safety and continued care, and to fulfill the requirement that critical study data should be verifiable.

To achieve this, the medical records of each patient should clearly describe at least:

- That the patient is participating in the study, e.g. by including the enrollment number and the study code or other study identification;
- Date when Informed Consent was obtained from the patient and statement that patient received a copy of the signed and dated Informed Consent;
- Results of all assessments confirming a patient's eligibility for the study;
- Diseases (past and current; both the disease studied and others, as relevant);
- Treatments withdrawn/withheld due to participation in the study;
- Results of assessments performed during the study;
- Treatments given, changes in treatments during the study and the time points for the changes;
- Non-Serious Adverse Events and Serious Adverse Events (if any) including causality assessments;
- Date of, and reason for, discontinuation from study treatment;

- Date of, and reason for, withdrawal from study;
- Additional information according to local regulations and practice.

The records for each participant should contain information which is important for the participant's safety and to fulfill the requirement that critical study data should be verifiable.

8.3 Study Monitoring

The investigator will be visited on a regular basis by the Clinical Study Monitor. For details, please refer to the Monitoring Plan. The Clinical Trials Unit at Oslo University Hospital is responsible for monitoring the study.

8.4 Confidentiality

The investigator shall arrange for the secure retention of the patient identification and the code list. Patient files shall be kept for the maximum period of time permitted by each hospital. The study documentation (CRFs, Site File etc.) shall be retained and stored during the study and for 15 years after study closure. All information concerning the study will be stored in a safe place inaccessible to unauthorized personnel.

8.5 Database management

Data management will be performed by the study group. The Data management procedures will be performed in accordance with NorCRIN guidelines http://www.norcrin.no/dokumentoversikt/. The data management process will be described in the study specific data handling plan and the study specific data handling report after database closure.

Data entered into EpiData will be validated as defined in the data validation plan. Validation includes, but is not limited to, validity checks (e.g. range checks), consistency checks and customized checks. A majority of edit checks will be triggered during data entry and will therefore facilitate efficient 'point of entry' data cleaning.

Data management personnel will perform both manual EpiData review and review of additional electronic edit checks to ensure that the data are complete, consistent and reasonable. The electronic edit checks will run continually throughout the course of the study and the issues will be reviewed manually online to determine what action needs to be taken.

Manual queries may be added to the system by clinical data management or study monitor. Clinical data managers and study monitors are able to remotely and proactively monitor the patient eCRFs to improve data quality.

All updates to queried data will be made by authorized study center personnel only and all modifications to the database will be recorded in an audit trail. Once the queries have been resolved, eCRFs will be signed by electronic signature. Any changes to signed eCRFs will be approved and resigned by the Investigator.

Once the full set of eCRFs have been completed and locked, the Sponsor will authorize database lock and all electronic data will be sent to the designated statistician for analysis. Subsequent changes to the database will then be made only by written agreement.

The data will be stored in a dedicated and secured area at Oslo University Hospital. Data will be stored in a de-identified manner, where each study participant is recognisable by his/her unique trial participant number. The data will be stored until 31/12 2035.

9 STATISTICAL METHODS AND DATA ANALYSIS

9.1 Sample Size calculation

In this study, we will include 250 women.

The sample calculation was based on a randomized controlled trial by Dencker et al from 2009 (37) and a review by Neal et al (27). In the trial, Dencker et al randomized 630 Swedish women who developed slow progress in the first part of active labor. Their inclusion criteria, definitions and time of randomization are similar to what is planned for our study, but they measured the duration from randomization to delivery, in contrast to our scope, which is the duration from IMP is given to delivery. Still, descriptive statistics from their "Early oxytocin group" is important for our sample size calculations. Dencker et al. found that in this group, the mean duration from randomization to delivery was 5.2 hours, SD 2.8 hours. In the review by Neal et al., a total of 17 studies and 7009 nulliparous women were summarized to have a weighted mean duration of labor at 360 minutes (6 hours), and a standard deviation of 216 minutes (3.5 hours). The article states that the mean values "closely parallel those found at median." Labor duration was calculated from onset of active phase, first stage, with a mean dilatation at start of active phase at 3.7 cm.

Our study will define active labor from 3 cm, and measure the duration from IMP is given to delivery. Since the intervention will not start until a woman crosses the alert line, the duration measured in the planned study, and the corresponding SD, will be expected to be shorter than the total duration and SD reported in Neal et al., which corresponds well to the numbers found in Dencker et al..

A mean difference of 60 minutes in labor duration is considered to be clinically relevant. Such a difference may be assumed to reduce the use of oxytocin, and it is realistic to obtain as all the other studies on spasmolytics found a difference of more than 55 minutes, with a mean difference all studies of 74 minutes.

A methodological issue is that while both the articles we use as a basis for the sample calculations report means and SDs for the duration of labor, this outcome is a time-to-event outcome, with emergency cesarean delivery as a censoring event. The primary outcome will be treated as such in the analyses, and this should also be reflected in the power calculation. However, background data on our primary endpoint are hard to find, and we have therefore based our sample size calculation on the results from Dencker et al.

In the study population, the expected proportion of emergency cesarean delivery will be approximately 7%. Hence, most (approximately 93%) duration times will be observed, and a sample size based on a traditional continuous outcome e.g. a two-sample t-test will give a good indication for the sample size. Due to the inherent skewness of labor duration data, one can expect published values of SD to be slightly overestimated.

With the SD of 2.8 given in Dencker et al., and a power of 80 % we would need in total 246 women (123 women in each group) to discover a difference of 60 minutes. Furthermore, we calculate that

we will need written informed consent from at least 600 women to be able to randomize approximately 250 participants (8% [50 women] will be lost because of elective cesarean section, 25% [140 women] will be induced or receive oxytocin before 3 cm, and 40% [165 women] will not cross the alert line). In addition some of the eligible women will not be randomized because the midwife on duty forget to take the necessary steps and some participants will withdraw from the study.

9.2 Randomization

9.2.1 Allocation- sequence generation

- The allocation sequence is based on computer-generated random numbers, and will be generated by an independent statistician
- the allocation ratio is 1 (equal probabilities to placebo and treatment)
- No stratification.

9.2.2 Allocation- procedure to randomize a patient

The midwife in charge of the patients in labor will enroll eligible participants. The participants will be assigned to treatment based on prespecified allocation envelopes with consecutive numbers that are kept in a locked room in the maternity ward, which is a separate ward on a different floor than the delivery ward. The numbered envelopes contain information about what IMP to prepare. When the midwife in charge on the maternity ward opens the envelope, the allocated treatment is revealed. The midwife in charge on the maternity ward is the only person who will know which treatment is given. After the allocated treatment is revealed, the midwife seals the envelope, and labels the envelope with the participant's name and date of birth.

Only personnel authorized by the principal investigator for preparing treatment will have access to treatment allocation, and the allocation will not be available until the patient has signed the informed consent form and deemed eligible to participate in the study. That is, authorized personnel will only know the allocation of included patients, but not for future patients. Once the patient has been included, the midwife in charge authorized for treatment preparation will reveal the allocation. Further procedure will be as follows:

1. Prepare the allocated treatment for administration
2. Stick the participant's name tag next to the unique allocation number on the prespecified allocation list
3. Sign the allocation list
4. Prepare and append label to the IMP in the same way that we do in clinical practice, with patient name, data of birth and allocated trial number
5. Dispatch all packaging identifying the allocated treatment
6. Lock the room with the envelopes to prevent blinded site staff knowledge of allocation
7. A third person, blinded to the content of the IMP, will bring the IMP from the maternity ward to the delivery ward

Site staff should take measures to maintain the blinding, e.g. not storing medication such that it is evident which treatment has been given by identifying missing medication.

9.2.3 Blinding and emergency unblinding

All patients and all labor ward personnel taking care of the patients during labor and birth, the investigators, the data analyst and the statistician will be blinded. The treatment drug or placebo will be prepared by the midwife in charge at the maternity ward, once a patient is included in the study. The midwife in charge on the maternity ward will not be involved in the delivery at any time. The midwife in charge on the maternity ward will be the only person not blinded.

The midwife in charge on the maternity ward will give the prepared IMP to a third person, who is blinded, and will give the IMP to the midwife on duty who takes care of the participant.

In the event of an SAE, the Investigator may only break the treatment code if the appropriate management of the patient necessitates immediate knowledge of the current treatment (in case of severe anaphylactic reactions). Although it is advantageous to retain the blind for all patients prior to final trial analysis, when an SAE may be a serious adverse reaction unexpected or otherwise judged reportable on an expedited basis, it is recommended that the blind should be broken only for that specific patient, by the CTU, even if the Investigator has not broken the blinding.

Unblinding of the treatment allocation is permissible only if the safety and well-being of the patient is being compromised. The decision to reveal the treatment allocation during the study may only be done by the principal investigator or the attending physician on duty. If the doctors are delayed, and the SAE is considered to be life-threatening, the midwife in charge can decide to reveal the treatment allocation. If neither the doctor nor the midwife in charge is available, the midwife on duty who takes care of the participant may open the envelope to reveal the allocation. In this way, only the allocation for the involved participant will be revealed. The date and time of un-blinding must be documented in the eCRF and in the patient's hospital records.

9.3 Population for Analysis

The following populations will be considered for the analyses:

- Intention to treat (ITT) population: All randomized participants, regardless of protocol adherence.

- Per-protocol population (PP): Includes all participants who have followed the protocol, i.e. one dose of the study medication (IMP). Randomized patients who were not eligible are excluded.

- Safety population: Includes all participants who have received one dose of study medication IMP. Participants who withdraw from the study will be included in the safety analysis. A list of withdrawn participants, preferably with the reasons for withdrawal, will be made.

The primary population is the ITT population.

9.4 Planned analyses

The main statistical analysis is planned when

- The planned number of patients has been included

- All included patients have either finalized their last assessment or have/are withdrawn according to protocol procedures

- All data have been entered, verified and validated according to the data management plan

Prior to the main statistical analysis, the data base will be locked for further entering or altering of data. A separate statistical analysis plan (SAP) will provide further details on the planned statistical analyses. The SAP will be finalized, signed and dated prior to database lock. The treatment allocation will be revealed after the database lock and used in the statistical analysis.

No interim analysis is planned.

Deviation from the original statistical plan will be described and justified in the Clinical Study Report. Amendments to plan can be done until day of DB lock.

9.5 Statistical Analysis

We will make a SAP as a separate document that fully details the planned analyses. The statistical analyses will be pre-specified in full detail in the SAP.

9.5.1 Primary analysis

This protocol is designed to address a single primary endpoint, which is the duration of labor from when the participant was given IMP, to delivery

This is a time-to-event outcome, where vaginal delivery is the event of interest and emergency cesarean delivery is the censoring event.

The study is designed to establish the superiority of intravenously administered Butylscopolamine bromide compared to placebo on a single primary endpoint; the duration of labor from when the participant was given IMP, to delivery. The null hypothesis is that there is no difference in the duration of labor from when the participant was given IMP, to delivery, between the two treatment groups.

The alternative hypothesis is that there is a difference in the duration of labor from when the participant was given IMP, to delivery, between the two treatment groups.

Superiority of Butylscopolamine bromide on the duration of labor from when the participant was given IMP, to delivery, is claimed if the primary null hypothesis is rejected, and the group difference is in favor of Butylscopolamine bromide.

Statistical significance is claimed if the null hypothesis is rejected on the significance level (alpha) of 0.05 (two-sided). That is, if the p-value of the null hypothesis test is less than or equal to 0.05.

As there is only one identified primary analysis, there will be no adjustments for multiple testing in the secondary analyses. Secondary analyses on secondary endpoints will be regarded as supportive.

The primary endpoint will be analyzed using Weibull regression, and the p-value for the statistical hypothesis test for the primary endpoint will be the p-value from this analysis. Unadjusted Kaplan-Meier plots will be presented. The effect measures will include median (and quartiles) duration in the two treatment groups, and the proportion of women who give birth within 2, 4, 6, 8, 10 and 12

hours after randomization. All efficacy analyses will be presented by the size (point estimate) of the difference between the treatments and the associated 95% confidence interval.

The statistical analyses will be done in SPSS v23, or R version 3.5.0 (https://www.r-project.org/).

Robustness of the results will be checked using Cox regression, by including baseline characteristics known to be associated with labor duration as covariates, and performing the analyses with different options for data imputation.

9.5.2 Secondary analyses

Secondary endpoints in this study contain

1. Time-to-event variables (duration of labor from the time when the participant was given IMP to 10 cm dilatation, and duration of labor from the onset of active labor (at least 3 cm dilatation) to delivery,

2. Continuous variables (mean cervical dilatation, amount of oxytocin given, pain scores on VAS scales, pH values from umbilical artery and vein, postpartum hemorrhage and responses to CEQ),

3. Categorical or discrete variables (mode of delivery, admission to Neonatal Intensive Care Unit, urinary retention, anal sphincter injuries and Apgar scores).

Time-to-event variables will be analyzed as the primary endpoint.

Continuous and categorical variables will be analyzed by simple bivariate analyses (t-test, Mann-Whitney test or Pearson chi square test, as appropriate), with linear or logistic regression as robustness analyses.

9.5.3 Background and Demographic Characteristics

Patient demographics and baseline characteristics will be summarized for all participants and by randomization group.

9.5.4 Exploratory Analysis

Hypothesis generating exploratory analyses will be performed on exploratory endpoints. These analyses will be regarded as post-hoc analyses.

9.5.5 Safety analyses

Safety analyses are limited to descriptive statistics and tabulations.

9.5.6 Other analyses (e.g. health economics, patient reported outcomes etc.)

Patient reported outcomes include pain score before and 30 minutes after IMP is given and birth experience four weeks after birth.

9.5.7 Descriptive statistics

This section should describe how descriptive statistics will be presented, e.g. demographics and baseline characteristics and patient flow.

10 STUDY MANAGEMENT

10.1 Investigator Delegation Procedure

The principal investigator is responsible for making and updating a "delegation of tasks" listing all the involved co-workers and their role in the project. He will ensure that appropriate training relevant to the study is given to all of these staff, and that any new information of relevance to the performance of this study is forwarded to the staff involved.

10.2 Protocol Adherence

Investigators ascertain they will apply due diligence to avoid protocol deviations.
All significant protocol deviations will be recorded and reported in the Clinical Study Report (CSR).

10.3 Study Amendments

If it is necessary for the study protocol to be amended, the amendment and/or a new version of the study protocol (Amended Protocol) must be notified to and approved by the Competent Authority and the Ethics Committee according to EU and national regulations.

10.4 Audit and Inspections

Authorized representatives of a Competent Authority and Ethics Committee may visit the center to perform inspections, including source data verification. Likewise the representatives from sponsor may visit the center to perform an audit. The purpose of an audit or inspection is to systematically and independently examine all study-related activities and documents to determine whether these activities were conducted, and data were recorded, analyzed, and accurately reported according to the protocol, Good Clinical Practice (ICH GCP), and any applicable regulatory requirements. The principal investigator will ensure that the inspectors and auditors will be provided with access to source data/documents.

11 ETHICAL AND REGULATORY REQUIREMENTS

The study will be conducted in accordance with ethical principles that have their origin in the Declaration of Helsinki and are consistent with ICH/Good Clinical Practice and applicable regulatory requirements. Registration of patient data will be carried out in accordance with national personal data laws.

11.1 Ethics Committee Approval

The study protocol for the pilot study has been approved by the Regional Committee of Medical and Health Research Ethics (Reference Number 2018/2380).

The investigator is responsible for informing the ethics committee of any serious and unexpected adverse events and/or major amendments to the protocol as per national requirements.

11.2 Other Regulatory Approvals

The study has been approved by the Norwegian Medicines Agency (18/09179-14).

The protocol will also be registered in www.clinicaltrials.gov before inclusion of the first patient.

11.3 Informed Consent Procedure

The investigator is responsible for giving the patients full and adequate verbal and written information about the nature, purpose, possible risk and benefit of the study. They will be informed as to the strict confidentiality of their patient data, but that their medical records may be reviewed for trial purposes by authorized individuals other than their treating physician.

It will be emphasized that the participation is voluntary and that the patient is allowed to refuse further participation in the protocol whenever she wants. This will not prejudice the patient's subsequent care. Documented informed consent must be obtained for all patients included in the study before they are registered in the study. This will be done in accordance with the national and local regulatory requirements. The investigator is responsible for obtaining signed informed consent.

A copy of the patient information and consent will be given to the patients. The signed and dated patient consent forms will be filed in the Investigator Site File binder.

11.4 Participant Identification

The investigator is responsible for keeping a list of all patients (who have received study treatment or undergone any study specific procedure) including patient's date of birth and personal number, full names and last known addresses.

The patients will be identified in the CRFs by patient number.

12 TRIAL SPONSORSHIP AND FINANCING

The study is sponsored by funds from Oslo University Hospital. The funds are assigned Lise Gaudernack.

13 TRIAL INSURANCE

The Principal Investigator has insurance coverage for this study through membership of the Drug Liability Association (see http://www.laf.no for more details).

14 PUBLICATION POLICY

Upon study completion and finalization of the study report the results of this study will either be submitted for publication and/or posted in a publicly assessable database of clinical study results.

The results of this study will also be submitted to the Competent Authority and the Ethics Committee according to EU and national regulations.

All personnel who have contributed significantly with the planning and performance of the study (Vancouver convention 1988) may be included in the list of authors.

15 REFERENCES

1. Cheng YW, Shaffer BL, Bryant AS, Caughey AB. Length of the first stage of labor and associated perinatal outcomes in nulliparous women. Obstet Gynecol. 2010;116(5):1127-35.
2. Lavender T, Wallymahmed AH, Walkinshaw SA. Managing labor using partograms with different action lines: a prospective study of women's views. Birth. 1999;26(2):89-96.
3. Le Ray C, Fraser W, Rozenberg P, Langer B, Subtil D, Goffinet F, et al. Duration of passive and active phases of the second stage of labour and risk of severe postpartum haemorrhage in low-risk nulliparous women. Eur J Obstet Gynecol Reprod Biol. 2011;158(2):167-72.
4. Nystedt A, Hogberg U, Lundman B. The negative birth experience of prolonged labour: a case-referent study. J Clin Nurs. 2005;14(5):579-86.
5. Norsk-gynekologisk-forening. http://legeforeningen.no/Fagmed/Norsk-gynekologisk-forening/Veiledere/Veileder-i-fodselshjelp-2014/Stimulering-av-rier/ 2014 [
6. Berglund S, Grunewald C, Pettersson H, Cnattingius S. Severe asphyxia due to delivery-related malpractice in Sweden 1990-2005. BJOG. 2008;115(3):316-23.
7. Milsom I, Ladfors L, Thiringer K, Niklasson A, Odeback A, Thornberg E. Influence of maternal, obstetric and fetal risk factors on the prevalence of birth asphyxia at term in a Swedish urban population. Acta Obstet Gynecol Scand. 2002;81(10):909-17.
8. Oscarsson ME, Amer-Wahlin I, Rydhstroem H, Kallen K. Outcome in obstetric care related to oxytocin use. A population-based study. Acta Obstet Gynecol Scand. 2006;85(9):1094-8.
9. Bernitz S, Oian P, Rolland R, Sandvik L, Blix E. Oxytocin and dystocia as risk factors for adverse birth outcomes: a cohort of low-risk nulliparous women. Midwifery. 2014;30(3):364-70.
10. Waldenstrom U. Experience of labor and birth in 1111 women. J Psychosom Res. 1999;47(5):471-82.
11. Clark SL, Simpson KR, Knox GE, Garite TJ. Oxytocin: new perspectives on an old drug. Am J Obstet Gynecol. 2009;200(1):35 e1-6.
12. Rygh AB, Skjeldestad FE, Korner H, Eggebo TM. Assessing the association of oxytocin augmentation with obstetric anal sphincter injury in nulliparous women: a population-based, case-control study. BMJ Open. 2014;4(7):e004592.
13. Leach S. 'Have you had a wee yet?' Postpartum urinary retention. Pract Midwife. 2011;14(1):23-5.
14. Nyflot LT, Sandven I, Stray-Pedersen B, Pettersen S, Al-Zirqi I, Rosenberg M, et al. Risk factors for severe postpartum hemorrhage: a case-control study. BMC Pregnancy Childbirth. 2017;17(1):17.
15. Brimdyr K, Cadwell K, Widstrom AM, Svensson K, Neumann M, Hart EA, et al. The Association Between Common Labor Drugs and Suckling When Skin-to-Skin During the First Hour After Birth. Birth. 2015.
16. Buchanan SL, Patterson JA, Roberts CL, Morris JM, Ford JB. Trends and morbidity associated with oxytocin use in labour in nulliparas at term. Aust N Z J Obstet Gynaecol. 2012;52(2):173-8.
17. Selin L, Almstrom E, Wallin G, Berg M. Use and abuse of oxytocin for augmentation of labor. Acta Obstet Gynecol Scand. 2009;88(12):1352-7.
18. Rossen J. Hvor mange kvinner stimuleres med oxytocin under fødselen Fødselsnytt nr 2 2013

https://www.fhi.no/hn/helseregistre-og-registre/mfr/; 2013 [
19. Gaudernack L, Frøslie K, Michelsen T, Voldner N, Lukasse M. De-medicalization of birth by reducing the use of oxytocin for augmentation among first-time mothers–a prospective intervention study. BMC Pregnancy and Childbirth. 2018;18(1):76.
20. Leppert PC. Anatomy and physiology of cervical ripening. Clin Obstet Gynecol. 1995;38(2):267-79.
21. Buhimschi IA, Buhimschi CS, Pupkin M, Weiner CP. Beneficial impact of term labor: nonenzymatic antioxidant reserve in the human fetus. Am J Obstet Gynecol. 2003;189(1):181-8.

22. Rohwer AC, Khondowe O, Young T. Antispasmodics for labour. Cochrane Database Syst Rev. 2013(6):CD009243.
23. Wilson CM, Lillywhite N, Matta B, Mills P, Wiltshire S. Intravenous buscopan for analgesia following laparoscopic sterilisation. Anaesthesia. 1999;54(4):389-92.
24. legemiddelinformasjon Rp. http://www.helsebiblioteket.no/pasientinformasjon/oppslagsverk/relis-legemiddelinformasjon 22.10.17 [
25. RELIS. Butylskopolamin og hjeteytmepåvirkning hos foster https://relis.no/sporamslogsvar/3-6107?source=relisdb 2010 [08.03.2010:[

26. Kirim S, Asicioglu O, Yenigul N, Aydogan B, Bahat N, Bayrak M. Effect of intravenous hyoscine-N-butyl bromide on active phase of labor progress: a randomized double blind placebo controlled trial. J Matern Fetal Neonatal Med. 2015;28(9):1038-42.
27. Neal JL, Lowe NK, Ahijevych KL, Patrick TE, Cabbage LA, Corwin EJ. "Active labor" duration and dilation rates among low-risk, nulliparous women with spontaneous labor onset: a systematic review. J Midwifery Womens Health. 2010;55(4):308-18.
28. Zhang J, Troendle J, Mikolajczyk R, Sundaram R, Beaver J, Fraser W. The natural history of the normal first stage of labor. Obstet Gynecol. 2010;115(4):705-10.
29. T F. Legemiddelhåndboka for helsepersonell 2013. . Fagbokforlaget Vigmostad & Bjørke AS; 2013.
30. Felleskatalogen. .https://www.felleskatalogen.no/medisin/buscopan-sanofi-aventis-547153 [
31. WHO. WHO recommendations for augmentation of labour. http://www.who.int/reproductivehealth/publications/maternal_perinatal_health/augmentation-labour/en/ 2014 [
32. Qahtani NH, Hajeri FA. The effect of hyoscine butylbromide in shortening the first stage of labor: A double blind, randomized, controlled, clinical trial. Therapeutics and clinical risk management. 2011;7:495-500.
33. Makvandi S, Tadayon M, Abbaspour M. Effect of hyoscine-N-butyl bromide rectal suppository on labor progress in primigravid women: randomized double-blind placebo-controlled clinical trial. Croat Med J. 2011;52(2):159-63.
34. Samuels LA, Christie L, Roberts-Gittens B, Fletcher H, Frederick J. The effect of hyoscine butylbromide on the first stage of labour in term pregnancies. Bjog. 2007;114(12):1542-6.
35. Sekhavat L, Karbasi SA, Fallah R, Mirowliai M. Effect of hyoscine butylbromide first stage of labour in multiparus women. African health sciences. 2012;12(4):408-11.
36. Dencker A, Taft C, Bergqvist L, Lilja H, Berg M. Childbirth experience questionnaire (CEQ): development and evaluation of a multidimensional instrument. BMC Pregnancy Childbirth. 2010;10:81.
37. Dencker A, Berg M, Bergqvist L, Ladfors L, Thorsen LS, Lilja H. Early versus delayed oxytocin augmentation in nulliparous women with prolonged labour--a randomised controlled trial. BJOG. 2009;116(4):530-6.
