# Supplementary material for: The effect of intravenous hyoscine butylbromide on slow progress in labor (BUSCLAB): A double-blind randomized placebo-controlled trial
Source: PLoS Med. 2024 Mar 28;21(3):e1004352. doi: 10.1371/journal.pmed.1004352 (PMC11008832; doi:10.1371/journal.pmed.1004352)
Supplement: S2 Text — (PDF) [file pmed.1004352.s003.pdf]

STATISTICAL ANALYSIS PLAN for BUSCLAB

Administrative information:

Sponsor name	Department of Obstetrics, Oslo University Hospital
Sponsor address	Miriam Kim Nyberg<br>Head of Department<br>Department of Obstetrics<br>Oslo University Hospital<br>PO Box 4950 Nydalen<br>N-0424 Oslo<br>Norway<br><br>Telephone: +47 23 07 00 00<br>E-mail: mirnyb@ous-hf.no
EudraCT number / REC number	EudraCT number: 2018-002338-19<br>REC number: 2017/1385
Trial title	BUSCLAB – A Double-Blind Randomized Placebo-Controlled Trial Investigating the Effect of Intravenous Butylscopolamine Bromide to Prevent Slow Progress in Labor
Trial ID	BUSCLAB – Buscopan in Labor
Trial registration number	NCT03961165

SAP and protocol version:

SAP version and date	Version 1.0, May 19, 2022
Protocol version	BUSCLAB001 Version No. 14, May 26, 2021

SAP revision history:

Protocol version	SAP version	Section number changed	Description and reason for change	Date changed
1.0	1.0	NA	NA	May 19, 2022

SIGNATURE PAGE

PRINCIPAL/COORDINATING INVESTIGATOR:

Trond Melbye Michelsen, MD, PhD
Oslo University Hospital

Trond M. Michelsen — 19/05/2022

Signature — Date (dd/mmm/yyyy)

TRIAL STATISTICIAN:

Nina Gunnes, MSc, PhD
Oslo University Hospital

Nina Gunnes — 19/05/2022

Signature — Date (dd/mmm/yyyy)

QC STATISTICIAN [if applicable]:

Name, title
Affiliation

Signature — Date (dd/mmm/yyyy)

ABBREVIATIONS

AE	adverse event
BMI	body mass index
BUSCLAB	Buscopan in Labor
CTG	cardiotocography
CEQ	Childbirth Experience Questionnaire
CI	confidence interval
CSR	clinical study report
ECS	emergency cesarean section
HR	hazard ratio
IMP	investigational medical product
IU	international units
NICU	neonatal intensive care unit
OR	odds ratio
PI	principal investigator
SAE	serious adverse event
SAP	Statistical Analysis Plan
SD	standard deviation
TMF	Trial Master File
VAS	visual analogue scale
WHO	World Health Organization

TABLE OF CONTENTS

1 INTRODUCTION 5
- 1.1 Background and rationale 5
- 1.2 Trial Objectives 5

2 TRIAL METHODS 6
- 2.1 Trial Design 6
- 2.2 Randomization 6
- 2.3 Sample size 6
- 2.4 Statistical Framework 7
- 2.5 Statistical Interim Analyses and Stopping Guidance 8
- 2.6 Timing of Final Analysis 8
- 2.7 Timing of Outcome Assessments 8

3 STATISTICAL PRINCIPLES 8
- 3.1 Confidence Intervals and P values 8
- 3.2 Adherence and Protocol Deviations 9
- 3.3 Analysis Data Sets 9

4 TRIAL POPULATION 10
- 4.1 Screening Data, Eligibility and Recruitment 10
- 4.2 Withdrawal/Follow-up 10
- 4.3 Baseline Patient Characteristics 11

5 ANALYSIS 12
- 5.1 Outcome Definitions 12
- 5.2 Analysis Methods 17

6 SAFETY ANALYSES 23
- 6.1 Adverse Events 24
- 6.2 Clinical Laboratory Parameters 24
- 6.3 Vital Signs 24

7 STATISTICAL SOFTWARE 24

8 REFERENCES 25
- 8.1 Literature References 25
- 8.2 Reference to Data Handling Plan 25
- 8.3 Reference to the Trial Master File and Statistical Documentation 25

1 Introduction

1.1 Background and rationale

Prolonged labor is a common complication of childbirth, especially in primiparous women. It is associated with several adverse outcomes (e.g., negative birth experience, operative delivery, chorioamnionitis, and admission to the neonatal intensive care unit [NICU]).

Augmentation with oxytocin is widely used to treat slow progress in labor by increasing contractions. However, oxytocin has some potential serious side effects, such as fetal asphyxia, operative delivery, anal sphincter injuries, postpartum hemorrhage, postpartum urinary retention, delayed initiation of breastfeeding, and negative birth experience. Moreover, the efficacy of oxytocin is uncertain. Hence, there is a need to evaluate alternative or adjuvant treatments for slow progress in the first stage of spontaneous labor.

Antispasmodics, or spasmolytics, are drugs that relieve spasms of smooth muscle tissue. They can also be used to shorten duration of labor and, thus, contribute to the use of oxytocin being reduced. Antispasmodics have been shown to reduce the duration of the first stage of labor and shorten the total duration of labor, but no effect has been found on the duration of the second or third stage of labor. Studies on the effect of antispasmodics as a prevention of slow progress in labor are lacking.

Butylscopolamine bromide, which is marketed under the trade name Buscopan, is an antispasmodic drug with a rapid onset of less than 20 minutes. Reported side effects of butylscopolamine bromide include tachycardia and dryness of the mouth. The fetal heart rate might also be influenced when given near delivery. Central effects of butylscopolamine bromide are rare because the drug only to a small extent crosses the blood-brain barrier or crosses the placenta. Studies conducted to date seem to have a reassuring safety profile regarding undesirable maternal and neonatal events.

The Buscopan in Labor (BUSCLAB) study is powered to clarify whether butylscopolamine bromide has a role in first-time labor. Prolonged labor is defined as a dilatation rate less than 1 cm per hour, i.e., crossing the alert line of the World Health Organization (WHO) partograph in the active phase of labor.

If butylscopolamine bromide has the anticipated effect, labor will be shortened for women who receive the active drug. If so, use of oxytocin will be lower in the active treatment group, and side effects caused by oxytocin may be reduced.

1.2 Trial Objectives

This trial primarily aims at assessing the efficacy of intravenously administered Buscopan to prevent slow progress in labor compared with placebo in first-time mothers who cross the alert line for labor dystocia according to the WHO partograph.

1.2.1 Primary Objective

The primary objective of the trial is to compare the two treatment groups with respect to duration of labor from when the investigational medicinal product (IMP) is administered to vaginal delivery and assess whether Buscopan is superior to placebo.

1.2.2 Secondary Objectives

The secondary objectives of the trial involve comparing the two treatment groups with respect to the secondary outcomes (see Section 5.1.2).

1.2.3 Exploratory Objectives

The exploratory objectives of the trial involve comparing the two treatment groups with respect to the exploratory outcomes (see Section 5.1.3).

2 Trial Methods

2.1 Trial Design

BUSCLAB is an interventional study designed as a single-center double-blind phase III randomized placebo-controlled clinical superiority trial with a treatment allocation ratio of 1:1.

2.2 Randomization

The randomization process is described in detail in Section 9.2 in the study protocol.

The allocation sequence is based on computer-generated random numbers. A biostatistician who will not be involved in inclusion of participants, data handling, or statistical data analysis will assign participants to study drug or placebo by block randomization with randomly mixed block sizes of two, four, and six. The allocation ratio is 1:1 (equal probabilities to placebo and treatment). The midwife in charge of the women in the delivery ward enrolls eligible trial participants, i.e., trial participants fulfilling all of the inclusion criteria and none of the exclusion criteria. The participants are assigned to treatment based on prespecified allocation envelopes. Only personnel authorized by the principal investigator (PI) for preparing treatment will have access to the treatment allocation. The numbered envelopes contain information about what IMP to prepare (Buscopan or placebo). A different midwife in the postnatal ward, which is located at a different floor from the delivery ward, opens the envelope to reveal the allocated treatment. This midwife prepares the IMP and delivers it to the staff in the delivery ward, however, not directly to the midwife taking care of the participant.

2.3 Sample size

The sample-size calculations are described in detail in Section 9.1 in the study protocol.

The required sample size is based on the primary outcome (duration of labor from the time when the woman is given IMP to vaginal delivery). A difference in mean duration of labor of 60 minutes between the two treatment groups is considered clinically relevant. Approximately 250 women will be included in this study – 125 women in each group.

The sample calculation was based on a randomized controlled trial by Dencker et al. from 2009 and a review by Neal et al. from 2010.[1,2] In the trial, Dencker et al. randomized 630 Swedish women who developed slow progress in the first part of active labor. Their inclusion criteria, definitions, and time of randomization are similar to our trial, but they measured the duration from randomization to delivery, in contrast to our scope, which is the duration from IMP administration to delivery. Still, descriptive statistics of their early oxytocin group are important for our sample size calculations. Dencker et al. found that in this group, the mean duration from randomization to delivery was 5.2 hours (standard deviation [SD]: 2.8 hours).[1] In the review by Neal et al., a total of 17 studies and 7009 nulliparous women were summarized to have a weighted mean duration of labor at 360

minutes (6 hours) and SD of 216 minutes (3.5 hours).[2] The paper states that the mean values "closely parallel those found at median". Labor duration was calculated from onset of active phase, first stage, with a mean dilatation at start of active phase at 3.7 cm.

Our trial defines active labor from 3 cm and measures the duration from IMP administration to delivery. Since the intervention will not start until a woman crosses the alert line of the WHO partograph, the duration measured in the current trial and the corresponding SD are expected to be shorter than the total duration and SD reported in Neal et al., which corresponds well to the numbers found in Dencker et al.

A mean difference of 60 minutes in labor duration is considered to be clinically relevant. Such a difference may be assumed to reduce the use of oxytocin, and it is realistic to obtain as all the other studies on spasmolytics found a difference of more than 55 minutes, with a mean difference of 74 minutes.

A methodological issue is that while both of the papers that we use as a basis for the sample size calculation report means and SDs for the duration of labor, our primary outcome is a time-to-event outcome, with emergency cesarean section (ECS) as a censoring event. This should also be reflected in the sample size calculation. However, background data on our primary outcome are hard to find, and we have therefore based our calculation on the results from Dencker et al.

In study population of the current trial, the expected proportion of emergency cesarean delivery will be approximately 7%. Hence, a majority of the duration times (approximately 93%) will be observed, and a sample size calculation based on, e.g., a two-sample *t* test of a continuous outcome will give a good indication of the sample size. Due to the inherent skewness of labor duration data, one can expect published values of SD to be slightly overestimated.

With the SD of 2.8 given in Dencker et al. and a power of 80% we would need in total 246 women (123 women in each group) to discover a difference of 60 minutes.

2.4 Statistical Framework

The trial is designed to establish superiority of Buscopan over placebo for the primary outcome. Analyses of secondary and exploratory outcomes will be regarded as supportive and exploratory, respectively.

2.4.1 Hypothesis Test

In each analysis, the null hypothesis is defined as no difference between the two treatment groups with respect to the outcome in question. The corresponding two-sided alternative hypothesis states that the groups do in fact differ in that regard.

The null hypothesis in the analysis of the primary outcome is that the respective hazard rates of the time from IMP administration to vaginal delivery in the two treatment groups are equal, and the alternative hypothesis is that the hazard rates differ between the groups.

2.4.2 Decision Rule

Superiority of Buscopan over placebo is claimed if the primary null hypothesis is rejected at a two-sided significance level of 5% (that is, if the P value of the hypothesis test is less than 0.05) and the

treatment difference is in favor of the Buscopan group. See Section 5.2 for more details on the specific analyses.

2.5 Statistical Interim Analyses and Stopping Guidance

There will be no interim analyses in this trial.

The whole trial may be discontinued at the discretion of the PI or the sponsor in the instances listed below:

- Adverse events (AEs), including serious adverse events (SAEs), unknown to date in respect of their nature, severity, and duration
- Medical or ethical reasons affecting continued performance of the trial
- Difficulties in recruitment of women to the trial

Further details concerning guidelines to be used for treatment and trial discontinuation are provided in Sections 5.3 and 5.4 in the study protocol.

2.6 Timing of Final Analysis

The main statistical analysis is planned when the following items are completed:

- The required number of women have been included
- All included women have either finalized their last assessment, withdrawn from the trial, or been excluded from the trial according to protocol procedures
- All data have been entered, verified, and validated according to the Data Management Plan
- The database has been locked for further entering and/or altering of data

Results of the analyses of the various outcomes may be reported in separate publications.

2.7 Timing of Outcome Assessments

A trial flow chart is shown in Section 5.1 in the study protocol.

The primary outcome (duration of labor from IMP administration to vaginal delivery) is measured at delivery.

All secondary outcomes regarding the mother are measured during labor, at delivery, or shortly after delivery except for urinary retention, which is measured before the trial participant leaves the delivery ward, and birth experience, which is measured by the validated Childbirth Experience Questionnaire (CEQ) one month postpartum.[3]

All secondary outcomes regarding the child are measured shortly after delivery except for change in fetal heart rate 30 minutes after IMP administration, which is measured during labor.

All exploratory outcomes (cervical dilatation rate, maternal heart rate, indication for operative delivery, and fetal heart rate) are measured during labor.

3 Statistical Principles

3.1 Confidence Intervals and P values

All hypothesis tests in the efficacy analyses of primary, secondary, and exploratory outcomes will be two-sided. A treatment difference is denoted as statistically significant if the P value is less than the predefined significance level of 5%. Correspondingly, a confidence level of 95% will be used for the two-sided confidence interval (CI) for the treatment difference.

There will be no adjustments for multiplicity, as there is only one primary analysis in this trial.

3.2 Adherence and Protocol Deviations

3.2.1 Adherence to Allocated Treatment

Adherence to allocated treatment is defined as the trial participant receiving the prescribed dose of the IMP to which she has been randomized. The IMP is administered intravenously only once by the midwife in charge at the delivery ward. Hence, non-compliance is not expected to be a problem in this trial.

Adherence to allocated treatment will be presented by number and percentage of trial participants receiving the correct dose of the assigned IMP.

3.2.2 Protocol Deviations

The following items are defined as *major* protocol deviations that may affect the efficacy of the intervention in this trial:

- No IMP administered
- Incorrect IMP (i.e., IMP other than that allocated) administered
- Incorrect dose (i.e., not the prescribed dose) of IMP administered
- Study personnel and/or trial participant unblinded to the treatment allocation
- Incorrect data on outcomes collected and documented

The following item is defined as a *minor* protocol deviation:

- IMP administered more than 45 minutes after the last vaginal examination
- Failure to attend follow-up evaluation one month postpartum

All major and minor protocol deviations will be recorded, tabulated by type and treatment group, and reported in publications of the study. Sensitivity analyses excluding trial participants with major deviations will also be conducted to assess the impact on overall conclusions.

3.3 Analysis Data Sets

The intention-to-treat (ITT) set comprises all eligible, randomized trial participants with a signed informed consent, regardless of protocol adherence.

The full-analysis (FA) set comprises all trial participants in the ITT set who have received at least one dose of IMP.

The per-protocol (PP) set comprises all eligible, randomized trial participants with a signed informed consent who have followed the protocol, that is, received one prescribed dose of the same IMP to which they have been randomized. Women with any major protocol deviations as defined in Section 3.2.2 will be excluded from the PP set.

The safety set comprises all eligible, randomized trial participants having received at least one dose of IMP. Women in the safety set will be analyzed according to the IMP received.

The main analysis of each outcome will be based on the FA set unless otherwise specified in Section 5.2. Sensitivity analyses restricted to the PP set will also be conducted for comparison purposes.

4 Trial Population

4.1 Screening Data, Eligibility and Recruitment

The trial inclusion and exclusion criteria are specified in Sections 3.3 and 3.4, respectively, in the study protocol.

The number of screened women (i.e., women assessed for eligibility for the trial with respect to the inclusion/exclusion criteria), the number of eligible women, and the number of non-eligible women will be presented in a flow diagram according to the Consolidated Standards of Reporting Trials (CONSORT). The CONSORT flow diagram will include the following information:

- Number of screened women
    - Number and percentage of screened women eligible for the trial
        - Number and percentage of eligible women randomized to treatment
            - Number and percentage of randomized women receiving allocated treatment
                - Number and percentage of treated women allocated to the Buscopan group
                - Number and percentage of treated women allocated to the placebo group
            - Number and percentage of randomized women *not* receiving allocated treatment (including reasons)
                - Number and percentage of non-treated women allocated to the Buscopan group
                - Number and percentage of non-treated women allocated to the placebo group
        - Number and percentage of eligible women *not* randomized to treatment (including reasons)
    - Number and percentage of screened women *not* eligible for the trial (including reasons)

In the event of any woman receiving a treatment other than that assigned (e.g., Buscopan given to a woman allocated to the placebo group), this will be included in the flow diagram (including reasons).

The number of women withdrawing or being lost to follow-up at the various stages of the trial will also be included in the flow diagram (including reasons). Loss to follow-up only involves failure to complete the CEQ one month postpartum and does not affect the observation of any of the other outcomes.

4.2 Withdrawal/Follow-up

Treatment discontinuation is not an issue in the current trial, as IMP is administered intravenously as a single dose. The possible levels of withdrawal and loss to follow-up are as follows:

- Withdrawal, or loss to follow-up before randomization
- Withdrawal, or loss to follow-up after randomization but before receiving the allocated treatment
- Withdrawal or loss to follow-up after receiving the allocated treatment

Women who are withdrawn from the trial or lost to follow-up after randomization cannot be replaced. However, women erroneously included in the trial, that is, non-eligible women who are included in the trial even though not all of the inclusion criteria are fulfilled and/or at least one the exclusion criteria is fulfilled, may be replaced.

The status of all eligible, randomized women at the end of the trial may be defined by the following potentially overlapping categories:

- Given IMP and assessed for outcomes
- Given IMP but not assessed for outcomes
- Not given IMP but assessed for outcomes
- Not given IMP and not assessed for outcomes
- Withdrawn from the trial
- Lost to follow-up

Note that a woman may fall into more than one category, e.g., if she is given IMP and assessed for outcomes but then subsequently withdraws or is lost to follow-up before the follow-up evaluation one month postpartum. The number of women within each category (or combination of categories) will be presented in a CONSORT flow diagram or tabulated by treatment group.

A list of trial participants withdrawn from the trial or lost to follow-up will be made, preferably with the reasons for the incomplete follow-up (if available).

The number of women included in the FA set and the respective numbers of women corresponding to withdrawal and loss to follow-up will be presented in a CONSORT flow diagram or tabulated by treatment group.

More information regarding withdrawal and participant discontinuation is given in Sections 5.3 and 5.4 in the study protocol.

4.3 Baseline Patient Characteristics

Baseline is defined as the point in time when the trial participant crosses the alert line of the WHO partograph and slow progress in labor is detected, that is, immediately before randomization.

Baseline characteristics of the trial participants include age, marital status, higher education, height, weight, chronic diseases, and medications used during pregnancy. Marital status, higher education, chronic diseases, and medications used during pregnancy are considered as dichotomous variables, whereas age, height, and weight are considered as continuous variables.

Age (in years) is calculated as date of baseline minus date of birth and is given as a decimal number. It will further be categorized as follows: < 25 years, 25–29 years, 30–34 years, and ≥ 35 years.

A continuous variable for body mass index (BMI) will be derived from height (in meters) and weight (in kilograms) and calculated as weight divided by height squared. BMI will further be categorized according to the WHO's definitions of underweight (BMI: < 18.5 kg/m^2), normal weight (BMI: 18.5–24.9 kg/m^2), overweight (BMI: 25.0–29.9 kg/m^2), and obesity (BMI: ≥ 30.0 kg/m^2).

Pain score (integer from 0 to 10) and maternal and fetal heart rates (in beats per minute) are also measured at baseline and considered as continuous variables. In addition, cervical dilation (in cm) is measured, which is considered as a continuous variable. If the IMP is given more than 45 minutes after the last vaginal examination, cervical dilation will be estimated at IMP administration by using linear interpolation. The number and percentage of women for whom this applies will be reported.

Baseline characteristics, including pain score, maternal and fetal heart rates, and cervical dilatation, will be summarized and tabulated by treatment group for the trial participants included in the FA set. Descriptive statistics of categorical variables will include frequency counts and percentages, whereas descriptive statistics of continuous variables will include mean, SD, median, lower and upper quartiles, and range.

Any potential imbalance between the two treatment groups with respect to baseline characteristics will be tested by using Pearson's chi-square test or Fisher's exact test (as appropriate) for categorical data and Student's two-sample *t* test or the Mann-Whitney *U* test (as appropriate) for continuous data.

5 Analysis

This trial is designed to address a single primary outcome, of which there is only one identified main analysis. All other efficacy analyses of the secondary and exploratory outcomes will be regarded as supportive and exploratory, respectively.

Efficacy analyses will be based primarily on the FA set (see Section 3.3). In the event of the FA set not being identical to the PP set due to major protocol deviations for any of the eligible, randomized trial participants, sensitivity analyses of the outcomes restricted to the PP set will be conducted as well. However, the FA set constitutes the primary analysis set.

There is only a single primary outcome in this trial. Hence, there will not be made adjustments for multiple testing in any of the analyses.

5.1 Outcome Definitions

5.1.1 Definition of Primary Outcome

5.1.1.1 Duration of labor from IMP administration to vaginal delivery

Duration of labor from IMP administration to vaginal delivery (in minutes) is derived as the difference between time of vaginal delivery and time of IMP administration. This is a time-to-event variable, with vaginal delivery as the event of interest and ECS as a competing event.

5.1.2 Definitions of Secondary Outcomes

5.1.2.1 Duration from IMP administration to cervical dilation of 10 cm
Duration from IMP administration to cervical dilation of 10 cm (in minutes) is derived as the difference between time of cervical dilation of 10 cm and time of IMP administration. This is a time-to-event variable, with cervical dilation of 10 cm as the event of interest and ECS as a competing event.

5.1.2.2 Mean cervical dilation rate
Mean cervical dilation rate (in cm per hour) is derived as the difference between 10 cm and cervical dilation at IMP administration divided by the difference between time of cervical dilation of 10 cm and time of IMP administration. This is a continuous variable.

5.1.2.3 Duration of labor from onset of active labor to vaginal delivery
Duration of labor from onset of active labor (i.e., cervical dilation of at least 3 cm) to vaginal delivery (in minutes) is derived as the difference between time of vaginal delivery and time of onset of active labor. This is a time-to-event variable, with vaginal delivery as the event of interest and ECS as a competing event.

5.1.2.4 Oxytocin augmentation
Duration of oxytocin augmentation (in minutes) is measured as total time of infusion of oxytocin during labor, excluding time intervals corresponding to cease in treatment. This is a continuous variable.

Amount of oxytocin is measured as total amount of oxytocin solution administered during labor and converted to international units (IU) based on a standardized concentration of the infusion solution (10 IU oxytocin per 1000 ml NaCl solution). This is a continuous variable.

5.1.2.5 Treatment with oral bicarbonate
Treatment with oral bicarbonate (no/yes) is registered during labor. This is a dichotomous variable.

5.1.2.6 Change in maternal heart rate 30 minutes after IMP administration
Change in maternal heart rate (in beats per minute) 30 minutes after IMP administration is derived as the difference between the maternal heart rates measured 30 minutes after IMP administration and at baseline, that is, right before IMP administration. This is a continuous variable.

5.1.2.7 Change in pain score 30 minutes after IMP administration
Change in pain score 30 minutes after IMP administration is derived as the difference between pain score measured 30 minutes after IMP administration and pain score measured at baseline, that is, right before IMP administration. Pain score is reported as an integer between 0 (“no pain”) and 10 (“maximum pain”). This is a continuous variable.

5.1.2.8 Obstetric anal sphincter injury
Obstetric anal sphincter injury is defined as an obstetric perineal tear of grade 3 (injury to perineum involving the anal sphincter complex) or grade 4 (injury to perineum involving the anal sphincter complex and anal epithelium). This is a dichotomous variable.

5.1.2.9 Mode of delivery
Operative delivery (forceps/vacuum delivery or ECS) vs. spontaneous vaginal delivery is derived from mode of delivery. This is a dichotomous variable.

ECS vs. vaginal delivery (spontaneous vaginal delivery or forceps/vacuum delivery) is derived from mode of delivery. This is a dichotomous variable.

Operative vaginal delivery (forceps/vacuum delivery) vs. spontaneous vaginal delivery is derived from mode of delivery. This is a dichotomous variable.

5.1.2.10 Postpartum hemorrhage
Postpartum hemorrhage (in ml) is measured after delivery. This is a continuous variable.

Postpartum hemorrhage ≥ 500 ml is derived from postpartum hemorrhage. This is a dichotomous variable.

Postpartum hemorrhage ≥ 1,000 ml is derived from postpartum hemorrhage. This is a dichotomous variable.

Postpartum hemorrhage ≥ 1,500 ml is derived from postpartum hemorrhage. This is a dichotomous variable.

5.1.2.11 Urinary retention
Urinary retention is defined as using a urinary catheter after delivery but before transfer from the delivery ward. This is a dichotomous variable.

5.1.2.12 Birth experience
Birth experience is measured one month postpartum by using the validated CEQ, which includes 22 questions.[3] Three of the questions are answered by putting a mark on a visual analogue scale (VAS) ranging from 0 to 100, and the VAS scores are subsequently categorized as 0–40 = 1, 41–60 = 2, 61–80 = 3, and 81–100 = 4. The remaining 19 questions are answered by using a four-point numeric rating scale from 1 to 4.

A total CEQ score is calculated as the mean score of all 22 questions. In addition, separate CEQ sub-scores are calculated as the mean score of questions related to the following four dimensions: “own capacity”, “professional support”, “perceived safety”, and “participation”. If more than half of the answers to the questions included in a specific CEQ score are missing, the score is set to missing. The CEQ scores are continuous variables.

5.1.2.13 Change in fetal heart rate 30 minutes after IMP administration
Change in fetal heart rate (in beats per minute) 30 minutes after IMP administration is derived as the difference between the fetal heart rates measured by cardiotocography (CTG) 30 minutes after IMP administration and at baseline, that is, right before IMP administration. This is a continuous variable.

5.1.2.14 Apgar score
5-minute Apgar score < 7 is derived from the Apgar score measured 5 minutes after delivery. This is a dichotomous variable.

10-minute Apgar score < 7 is derived from the Apgar score measured 10 minutes after delivery. This is a dichotomous variable.

5.1.2.15 pH level
The pH level of the umbilical artery is measured after delivery. This is a continuous variable.

A pH level of the umbilical artery < 7.0 is derived from the pH level of the umbilical artery measured after delivery. This is a dichotomous variable.

The pH level of the umbilical vein is measured after delivery. This is a continuous variable.

A pH level of the umbilical vein < 7.1 is derived from the pH level of the umbilical vein measured after delivery. This is a dichotomous variable.

5.1.2.16 Admission to the NICU

Admission of the neonate to the NICU directly from the delivery ward is registered within a couple of hours after delivery. This is a dichotomous variable.

5.1.3 Definitions of Exploratory Outcomes

5.1.3.1 Cervical dilation rate

Cervical dilation rate (in cm per hour) is derived from cervical dilation measured at different points in time from IMP administration to cervical dilation of 10 cm. This is a continuous variable as a function of time.

5.1.3.2 Maternal heart rate

Maternal heart rate (in beats per minute) is registered continuously for at least 30 minutes after IMP administration. This is a continuous variable as a function of time.

5.1.3.3 Indication for operative delivery

Indication for operative delivery is registered as labor dystocia, fetal distress, and other indications (see Section 1.8 in the study protocol). This is a nominal variable.

5.1.3.4 Fetal heart rate

Fetal heart rate (in beats per minute) is registered continuously for at least 30 minutes after IMP administration. This is a continuous variable as a function of time.

5.1.4 Overview of Outcomes

A short overview of all outcomes in this trial is shown in Table 1.

Table 1: Overview of primary, secondary, and exploratory outcomes.

Number	Level	Outcome	Type	Time frame
1	Primary	Duration of labor from IMP administration to vaginal delivery	Time-to-event variable	At delivery
2	Secondary	Duration from IMP administration to cervical dilation of 10 cm	Time-to-event variable	During labor
3	Secondary	Mean cervical dilation rate	Continuous variable	During labor
4	Secondary	Duration of labor from onset of active labor to vaginal delivery	Time-to-event variable	At delivery
5	Secondary	Duration of oxytocin augmentation	Continuous variable	During labor

6	Secondary	Amount of oxytocin	Continuous variable	During labor
7	Secondary	Treatment with oral bicarbonate	Dichotomous variable	During labor
8	Secondary	Change in maternal heart rate 30 minutes after IMP administration	Continuous variable	During labor
9	Secondary	Change in pain score 30 minutes after IMP administration	Continuous variable	During labor
10	Secondary	Obstetric anal sphincter injury	Dichotomous variable	At delivery
11	Secondary	Operative delivery	Dichotomous variable	At delivery
12	Secondary	Emergency cesarean section	Dichotomous variable	At delivery
13	Secondary	Operative vaginal delivery vs. spontaneous vaginal delivery	Dichotomous variable	At delivery
14	Secondary	Postpartum hemorrhage	Continuous variable	After delivery
15	Secondary	Postpartum hemorrhage ≥ 500 ml	Dichotomous variable	After delivery
16	Secondary	Postpartum hemorrhage ≥ 1,000 ml	Dichotomous variable	After delivery
17	Secondary	Postpartum hemorrhage ≥ 1,500 ml	Dichotomous variable	After delivery
18	Secondary	Urinary retention	Dichotomous variable	Before transfer from the delivery ward
19	Secondary	Total score on birth experience	Continuous variable	One month postpartum
20	Secondary	Sub-score on birth experience for “own capacity” dimension	Continuous variable	One month postpartum
21	Secondary	Sub-score on birth experience for “professional support” dimension	Continuous variable	One month postpartum
22	Secondary	Sub-score on birth experience for “perceived safety” dimension	Continuous variable	One month postpartum
23	Secondary	Sub-score on birth experience for “participation” dimension	Continuous variable	One month postpartum
24	Secondary	Change in fetal heart rate 30 minutes after IMP administration	Continuous variable	During labor

25	Secondary	5-minute Apgar score < 7	Dichotomous variable	5 minutes after delivery
26	Secondary	10-minute Apgar score < 7	Dichotomous variable	10 minutes after delivery
27	Secondary	pH level of the umbilical artery	Continuous variable	After delivery
28	Secondary	pH level of the umbilical artery < 7.0	Dichotomous variable	After delivery
29	Secondary	pH level of the umbilical vein	Continuous variable	After delivery
30	Secondary	pH level of the umbilical vein < 7.1	Dichotomous variable	After delivery
31	Secondary	Admission to the neonatal intensive care unit	Dichotomous variable	After delivery
32	Exploratory	Cervical dilation rate	Continuous variable as a function of time	During labor
33	Exploratory	Maternal heart rate	Continuous variable as a function of time	During labor
34	Exploratory	Indication for operative delivery	Nominal variable	During labor
35	Exploratory	Fetal heart rate	Continuous variable as a function of time	During labor

5.2 Analysis Methods

The main analysis of all outcomes will be based on the entire FA set with a few exceptions as listed below.

- The main analysis of mean cervical dilatation rate (secondary outcome) will be based on a subset of the FA set restricted to trial participants with a cervical dilatation of 10 cm before delivery.
- The main analysis of obstetric anal sphincter injury (secondary outcome) will be based on a subset of the FA set restricted to vaginal deliveries.
- The main analysis of operative vaginal delivery vs. spontaneous vaginal delivery (secondary outcome) will be based on a subset of the FA set restricted to vaginal deliveries.
- The main analysis of the CEQ scores (secondary outcomes) will be based on a subset of the FA set restricted to trial participants having completed the CEQ one month postpartum.
- The main analysis of indication for operative delivery (exploratory outcome) will be based on a subset of the FA set restricted to operative deliveries.

The crude (i.e., unadjusted) analysis is to be regarded as the main analysis for all outcomes except for changes of maternal heart rate, pain score, and fetal heart rate 30 minutes after IMP administration, of which the main analyses will be adjusted for maternal heart rate, pain score, and fetal heart rate, respectively, at baseline.

If there appears to be any imbalance with respect to categorical age or categorical BMI between the women in the two treatment groups (see Section 4.3), sensitivity analyses adjusting for the

variable(s) in which there is an imbalance will be conducted for all outcomes. Furthermore, it is reasonable to assume that duration of labor, cervical dilatation rate, and change in pain score will depend on labor progression at IMP administration. Hence, if there appears to be any imbalance with respect to cervical dilation before IMP administration (see Section 4.3), sensitivity analyses of the following outcomes will be adjusted for this variable:

- Duration of labor from IMP administration to vaginal delivery (primary outcome)
- Duration of labor from IMP administration to cervical dilatation of 10 cm (secondary outcome)
- Mean cervical dilatation rate (secondary outcome)
- Duration of labor from onset of active labor to vaginal delivery (secondary outcome)
- Change in pain score 30 minutes after IMP administration (secondary outcome)

The result of each efficacy analysis will be presented by the point estimate of the relevant effect measure of treatment difference (reported with two decimals) and the associated two-sided 95% CI (reported with two decimals) in addition to the P value of the corresponding two-sided hypothesis test (reported with three decimals).

5.2.1 Primary Outcome

5.2.1.1 Main Analysis

Duration of labor from IMP administration to vaginal delivery will be analyzed by Weibull regression. The null hypothesis of the analysis is defined as no difference in the hazard rate between the two treatment groups.

As vaginal delivery and ECS may be regarded as two competing events, ECS will first be treated as a censoring event, giving the cause-specific hazard ratio (HR) of vaginal delivery between women in the intervention group and women in the placebo group. Correspondingly, we will also use Weibull regression to analyze duration of labor from IMP administration to ECS, treating vaginal delivery as a censoring event.

The cause-specific HR of vaginal delivery between the treatment groups will be evaluated in light of the cause-specific HR of ECS. Given that the cause-specific HR of ECS does not indicate increased risk of ECS in the intervention group, superiority of Buscopan over placebo on the duration of labor from IMP administration to vaginal delivery is claimed if the null hypothesis of no difference between the treatment groups is rejected at the two-sided significance level of 5% and the treatment difference is in favor of the Buscopan group (i.e., cause-specific HR of vaginal delivery exceeding 1). This would imply a shorter duration of labor in the intervention group.

5.2.1.2 Summary Measures

The respective cause-specific HRs of vaginal delivery and ECS between women in the intervention group and women in the placebo group will be presented with associated two-sided 95% CIs and corresponding P values.

Curves of the cumulative incidences of vaginal delivery and ECS will be plotted by treatment group. The mean and median duration of labor from IMP administration to delivery will also be reported by treatment group and mode of delivery (vaginal delivery or ECS) in addition to the respective

numbers and percentages of women giving birth within 2, 4, 6, 8, 10, and 12 hours after IMP administration.

5.2.1.3 Assumption Checks and Alternative Analyses

Appropriateness of using Weibull regression in the main analysis will be checked. If the distributional assumptions are not satisfied, Cox proportional-hazards regression will be used in the main analysis unless the proportional-hazards assumption is violated, in which case a comparison of the cumulative incidences between the two treatment groups at 8 hours after IMP administration will be made instead.

5.2.1.4 Missing Data

Missing data assessments are based on a blinded review of the data, i.e., without knowledge about the treatment allocation of the trial participants.

Time of IMP administration and time of delivery are registered for all trial participants in addition to mode of delivery. Hence, there will be no missing data on the primary outcome, nor will there be any missing data on treatment allocation.

Furthermore, there will be no missing data on the potential adjustment variables in a sensitivity analysis in case of an imbalance in baseline characteristics (see Sections 4.3 and 5.2), as age, BMI, and cervical dilatation before IMP administration are registered for all trial participants.

5.2.1.5 Sensitivity Analyses

To check the robustness of the results of the main analysis of the FA set, a sensitivity analysis similar to the main analysis but restricted to the PP set will be conducted if the two analysis sets differ.

Robustness of the results of the main analysis will also be checked by using Cox proportional-hazards regression.

In case of an imbalance between the two treatment groups with respect to either of the variables categorical age, categorical BMI, and cervical dilatation before IMP administration (see Sections 4.3 and 5.2), a sensitivity analysis similar to the main analysis adjusting for the variable(s) causing the imbalance will be conducted.

5.2.1.6 Subgroup Analyses

To explore a potential treatment effect modification by labor progression before IMP administration, subgroup analyses similar to the main analysis will be conducted by including a binary variable indicating whether IMP was administered early or late in labor, as defined by cervical dilatation at IMP administration (< 5 cm or ≥ 5 cm), in addition to an interaction term between this variable and the binary treatment variable. The resulting cause-specific HRs of vaginal delivery and ECS between the two treatment groups in each labor progression subgroup will be presented with associated two-sided 95% CIs and corresponding P values in addition to the P values of the interaction term in both models.

No further subgroup analyses of the primary outcome are planned.

5.2.2 Dichotomous Secondary Outcomes

5.2.2.1 Main Analysis

Dichotomous secondary outcomes will be analyzed by binary logistic regression, with odds ratio (OR) as the effect measure. The null hypothesis of each analysis is defined as no difference in the odds of the outcome in question between the two treatment groups. The two-sided alternative hypothesis is that the odds in the intervention group differs from that in the placebo group.

The null hypothesis of no treatment effect is rejected if the P value from the analysis is less than the predefined two-sided significance level of 5%. If the treatment difference is in favor of the Buscopan group, this suggests a reduced risk of the outcome associated with the intervention (OR < 1), as all dichotomous outcomes in the current trial are considered adverse in nature.

5.2.2.2 Summary Measures
The OR of each outcome between the intervention group and the placebo group will be presented with the associated two-sided 95% CI and the corresponding P value.

The number and percentage of women with the outcome in question will be presented by treatment group.

5.2.2.3 Assumption Checks and Alternative Analyses
The appropriateness of binary logistic regression as the main analysis will be checked.

5.2.2.4 Missing Data
Missing data assessments are based on a blinded review of the data, i.e., without knowledge about the treatment allocation of the trial participants.

There are no missing data on any of the dichotomous outcomes except for obstetric anal sphincter injury (0.8%), 10-minute Apgar score (2.4%), pH level of the umbilical artery (32.9%), and pH level of the umbilical vein (13.3%). Furthermore, there are no missing data on treatment allocation.

Best-case analysis (i.e., best-case [single] imputation) will be used to handle missingness of dichotomous outcomes with a proportion of missing values less than 5%. The best-case scenario for obstetric anal sphincter injury is no perinatal tear or a tear of grade 1 or 2, whereas the best-case scenario for 10-minute Apgar score is ≥ 7. Missing values of the dichotomized pH levels of the umbilical artery and the umbilical vein will be handled by using multiple imputation of the corresponding continuous variables followed by dichotomization of these two variables. The variables that will be used in the imputation process include allocated treatment, categorical age, and categorical BMI. The imputation process will be conducted separately for the pH levels of the umbilical artery and the umbilical vein.

There will be no missing data on the potential adjustment variables in sensitivity analyses in case of an imbalance in baseline characteristics (see Sections 4.3 and 5.2), as age and BMI are registered for all trial participants.

5.2.2.5 Sensitivity Analyses
To check the robustness of the results of the main analysis of the FA set, a sensitivity analysis similar to the main analysis but restricted to the PP set will be conducted if the two analysis sets differ.

A simple bivariate analysis (Pearson’s chi-square test or Fisher’s exact test, as appropriate) will also be conducted.

Additional sensitivity analyses similar to the main analysis will be conducted by using complete-case analysis for all outcomes and worst-case (single) imputation of missing values of obstetric anal sphincter injury and 10-minute Apgar score. For pH levels of the umbilical artery and umbilical vein, sensitivity analyses similar to the main analysis will be conducted with a mutual imputation process for both pH levels, allowing for prediction of missing values of one pH level by observed values of the other pH level in addition to allocated treatment, categorical age, and categorical BMI.

In case of an imbalance between the two treatment groups with respect to the variables categorical age and categorical BMI (see Sections 4.3 and 5.2), a sensitivity analysis similar to the main analysis adjusting for the variable(s) causing the imbalance will be conducted.

5.2.2.6 Subgroup Analyses

No subgroup analyses are planned.

5.2.3 Continuous Secondary Outcomes

5.2.3.1 Main Analysis

Continuous secondary outcomes will be analyzed by linear regression. The null hypothesis of each analysis is defined as no difference in the mean outcome between the two treatment groups, and this is tested against the two-sided alternative hypothesis that the means do in fact differ.

The null hypothesis of no treatment effect is rejected if the P value from the analysis is less than the predefined two-sided significance level of 5%. For mean cervical dilation rate, outcomes related to birth experience (CEQ scores), and the respective pH levels of the umbilical artery and umbilical vein, a treatment difference in favor of the Buscopan group corresponds to a *positive* regression coefficient of the treatment allocation. Conversely, this corresponds to a *negative* regression coefficient of the treatment allocation for duration of oxytocin augmentation, amount of oxytocin, change in pain score 30 minutes after IMP administration, postpartum hemorrhage, and changes in maternal and fetal heart rates 30 minutes after IMP administration.

5.2.3.2 Summary Measures

The regression coefficient of treatment allocation will be presented with the associated two-sided 95% confidence interval and the corresponding P value.

Mean and SD or median and interquartile range (as appropriate) of each outcome will be presented by treatment group.

5.2.3.3 Assumption Checks and Alternative Analyses

The appropriateness of linear regression as the main analysis, including the normality assumption of the residuals, will be checked.

When the conditions of linear regression are not met, quantile regression will be used to estimate the difference in the median outcome between the two treatment groups instead.

5.2.3.4 Missing Data

Missing data assessments are based on a blinded review of the data, i.e., without knowledge about the treatment allocation of the trial participants.

There are a few missing values of maternal heart rate 30 minutes after IMP administration (0.4%), pain scores at baseline and 30 minutes after IMP administration (0.4% and 2.4%, respectively), and fetal heart rate 30 minutes after IMP administration (0.4%), resulting in missing values of change in maternal heart rate 30 minutes after IMP administration (0.4%), change in pain score 30 minutes after IMP administration (2.4%), and change in fetal heart rate 30 minutes after IMP administration (0.4%). In addition, there are a few missing values of pH level of the umbilical artery (32.9%) and pH level of the umbilical vein (13.3%). No other continuous outcomes have missing data. Moreover, there are no missing data on treatment allocation.

Mean (single) imputation, performed separately within each treatment group, will be used to handle missingness of continuous variables with a proportion of missing values less than 5%. For the three outcomes of change, missing values of the variables corresponding to baseline and 30 minutes after IMP administration will first be imputed before the changes are calculated. Missing values of pH levels of the umbilical artery and the umbilical vein will be handled by using multiple imputation. The variables that will be used in the imputation process include allocated treatment, categorical age, and categorical BMI. The imputation process will be conducted separately for the pH levels of the umbilical artery and the umbilical vein.

There will be no missing data on the potential adjustment variables in sensitivity analyses in case of an imbalance in baseline characteristics (see Sections 4.3 and 5.2), as age, BMI, and cervical dilatation before IMP administration are registered for all trial participants.

5.2.3.5 Sensitivity Analyses

To check the robustness of the results of the main analysis of the FA set, a sensitivity analysis similar to the main analysis but restricted to the PP set will be conducted if the two analysis sets differ.

In addition, a sensitivity analysis similar to the main analysis will be conducted by using complete-case analysis. For pH levels of the umbilical artery and umbilical vein, sensitivity analyses similar to the main analysis will be conducted with a mutual imputation process for both pH levels, allowing for prediction of missing values of one pH level by observed values of the other pH level in addition to allocated treatment, categorical age, and categorical BMI.

In case of an imbalance between the two treatment groups with respect to the variables categorical age and categorical BMI (see Sections 4.3 and 5.2), a sensitivity analysis similar to the main analysis adjusting for the variable(s) causing the imbalance will be conducted. In addition, sensitivity analyses of mean cervical dilatation rate and change in pain score 30 minutes after IMP administration will be adjusted for cervical dilatation before IMP administration in the event of an imbalance with respect to this variable (see Sections 4.3 and 5.2).

5.2.3.6 Subgroup Analyses

No subgroup analyses are planned.

5.2.4 Time-to-event Secondary Outcomes

5.2.4.1 Main Analysis

See Section 5.2.1.1.

For duration of labor from IMP administration to cervical dilatation of 10 cm, only ECS occurring *before* a cervical dilation of 10 cm is treated as a censoring event when estimating the cause-specific HR of cervical dilatation of 10 cm between women in the intervention group and women in the placebo group. Correspondingly, cervical dilatation of 10 cm is treated as a censoring event when estimating the cause-specific HR of ECS prior to a cervical dilatation less than 10 cm between the two treatment groups.

5.2.4.2 Summary Measures
See Section 5.2.1.2.

5.2.4.3 Assumption Checks and Alternative Analyses
See Section 5.2.1.3.

5.2.4.4 Missing Data
Missing data assessments are based on a blinded review of the data, i.e., without knowledge about the treatment allocation of the trial participants.

Time of onset of active labor, time of IMP administration, time of cervical dilatation of 10 cm (trial participants with a cervical dilatation of 10 cm before delivery only), time of delivery, and mode of delivery are registered for all trial participants. Hence, there are no missing values on any of the time-to-event outcomes, nor are there missing data on treatment allocation.

Furthermore, there will be no missing data on the potential adjustment variables in sensitivity analyses in case of an imbalance in baseline characteristics (see Sections 4.3 and 5.2), as age, BMI, and cervical dilatation before IMP administration are registered for all trial participants.

5.2.4.5 Sensitivity Analyses
See Section 5.2.1.5.

5.2.4.6 Subgroup Analyses
No subgroup analyses are planned.

5.2.5 Exploratory Outcomes
Analyses of exploratory outcomes will be regarded as hypothesis-generating post-hoc analyses and not detailed in the Statistical Analysis Plan (SAP).

Indication for operative delivery will typically be analyzed by binary/multinomial logistic regression, whereas functional data analysis may be used to analyze cervical dilatation rate, maternal heart rate, and fetal heart rate. However, these analyses might be altered along the way based on preliminary results in the pursuit of interesting clinical hypotheses.

6 Safety Analyses
Topics related to safety monitoring and reporting are described in detail in Section 7 in the study protocol.

Safety is monitored by assessments of the mother and child during labor, at delivery, and during the stay at the postnatal ward. A physical examination of the child includes continuous fetal heart rate tracing (CTG) and is performed at baseline (i.e., when the mother crosses the alert line), when IMP is

given, and 30 minutes after IMP is given. Vital signs of the mother include blood pressure, which is measured at hospital admission, and maternal heart rate, which is measured when IMP is given, on the day of delivery, twice a day during the stay at the postnatal ward, and on the day of discharge from the postnatal ward. In addition, a physical examination of the neonate is performed by a pediatrician on the first day postpartum, which includes an examination of general appearance. AEs of the mother and the neonate are recorded from IMP administration throughout the hospital stay.

The safety set comprises all eligible, randomized trial participants having received at least one dose of IMP. General safety evaluations will be based on the occurrence, intensity, and type of the AEs in addition to clinically significant changes in vital signs and physical examination parameters. Safety analyses are limited to descriptive statistics and tabulations of AEs (based on all AEs and on women with AEs) and maternal and fetal heart rates by received treatment for all women included in the safety set.

6.1 Adverse Events

Recording of AEs begins at IMP administration and continues throughout the hospital stay. All AEs are coded by using MedDRA, Version 22. The intensity of each AE is described as mild, moderate, or severe. AEs with missing intensity will be considered as severe. A potential causal relation between the study medication and an AE will classified as no or yes.

Only treatment-emerging AEs (TEAEs) and treatment-emerging SAEs (TESAEs), i.e., AEs and SAEs occurring after IMP administration, will be tabulated. Any AEs prior to IMP administration will not listed or tabulated.

The numbers and percentages of women with any TEAEs, women with 1, 2, and 3+, respectively, TEAEs, women with any TESAEs, and women with TEAEs leading to study discontinuation will be presented overall and by system organ class and treatment group.

The numbers of AEs and SAEs leading to study discontinuation and the numbers and percentages of women with AEs and SAEs leading to study discontinuation will be presented overall and by system organ class and treatment group. In addition, a summary table of AEs reported by at least 20% of the women in the safety population will be presented by system organ class and treatment group.

A detailed patient narrative will be given for any SAE in addition to listing. Tabulations by diagnosis will also be presented.

6.2 Clinical Laboratory Parameters

No clinical laboratory parameters were collected and assessed to identify adverse events in this trial.

6.3 Vital Signs

Vital signs (maternal blood pressure [mmHg], maternal heart rate [beats per minute], and fetal heart rate [beats per minute]) will be summarized by treatment group and points in time.

7 Statistical Software

Statistical analyses will mainly be conducted by using Stata for Windows (StataCorp LLC) and/or R for Windows (R Foundation for Statistical Computing). Some analyses will also be conducted by using IBM SPSS Statistics for Windows (IMB Corp).

8 References

8.1 Literature References

1. Dencker A, Berg M, Bergqvist L, Ladfors L, Thorsén LS, Lilja H. Early versus delayed oxytocin augmentation in nulliparous women with prolonged labour--a randomised controlled trial. *BJOG*. 2009;116(4):530-536. doi:10.1111/j.1471-0528.2008.01962.x
2. Neal JL, Lowe NK, Ahijevych KL, Patrick TE, Cabbage LA, Corwin EJ. "Active labor" duration and dilation rates among low-risk, nulliparous women with spontaneous labor onset: a systematic review. *J Midwifery Womens Health*. 2010;55(4):308-318. doi:10.1016/j.jmwh.2009.08.004
3. Dencker A, Taft C, Bergqvist L, Lilja H, Berg M. Childbirth experience questionnaire (CEQ): development and evaluation of a multidimensional instrument. *BMC Pregnancy Childbirth*. 2010;10:81. Published 2010 Dec 10. doi:10.1186/1471-2393-10-81

8.2 Reference to Data Handling Plan

The Data Management Plan, Version 1, is included in the Trial Master File (TMF), Section 12.1.

8.3 Reference to the Trial Master File and Statistical Documentation

The TMF and the statistical documentation are held separately in a password-protected database with restricted access and as a paper copy secured in a locked place. The statistical documentation includes details of the randomization process and specific protocol deviations that the statistician needs to refer to when executing the SAP.

The statistical documentation will be kept for 15 years for future reference.
