# Supplementary material for: The effect of intravenous hyoscine butylbromide on slow progress in labor (BUSCLAB): A double-blind randomized placebo-controlled trial
Source: PLoS Med. 2024 Mar 28;21(3):e1004352. doi: 10.1371/journal.pmed.1004352 (PMC11008832; doi:10.1371/journal.pmed.1004352)
Supplement: S1 Fig — (a) Investigational medicinal product (IMP). (b) Physical examination fetus included cardiotocography (CTG) with continuous fetal heart rate tracing. (c) Vital signs mother included blood pressure and pulse. Maternal height and body weight was obtained from the pregnancy chart. (d) Pain measurement using visual analogue score. (e) Physical examination newborn included an examination of general appearance. (PDF) [file pmed.1004352.s004.pdf]

	STUDY PERIOD						
**TIME**	**Screening Period**	**Delivery**			**End of postnatal stay**		**Follow-up evaluation**
**Time point**		**Baseline (≥3cm and < 10 cm dilation) Crossing the alert line**	**[a]IMP is given**	**30 min after IMP is given**	**Day of delivery**	**Day of discharge**	**1 month postpartum**
**ENROLMENT:**							
Informed consent	X						
Inclusion/exclusion Evaluation	X						
Medical History	X						
Concomitant Medication	X	X					
[b]Physical Examination fetus (CTG)		X	X	X			
[c]Vital signs mother		X	X		X	X	
Cervical dilation		X					
[d]Pain measurement (VAS)			X	X			
**INTERVENTIONS:**							
Treatment administration			X				
**ASSESSMENTS:**							
Adverse event, mother			X	X	X	X	
Adverse event, newborn/fetus			X	X	X	X	
[e]Physical Examination newborn (pediatrician)						1st day post partum	
Childbirth Experience Questionnaire							X
