# Supplementary material for: The effect of intravenous hyoscine butylbromide on slow progress in labor (BUSCLAB): A double-blind randomized placebo-controlled trial
Source: PLoS Med. 2024 Mar 28;21(3):e1004352. doi: 10.1371/journal.pmed.1004352 (PMC11008832; doi:10.1371/journal.pmed.1004352)
Supplement: Attachment — Submitted filename: Response to reviewers 080923 (1).docx [file pmed.1004352.s007.docx]

| Comments from: | Answer to Editors and reviewers |
| --- | --- |
|  |  |
| **EDITORIAL MANAGER** |  |
|  |  |
| To enhance the reproducibility of your results, we recommend that you deposit your laboratory protocols in protocols.io, where a protocol can be assigned its own identifier (DOI) such that it can be cited independently in the future. Additionally, PLOS ONE offers an option to publish peer-reviewed clinical study protocols. Read more information on sharing protocols at <https://plos.org/protocols?utm_medium=editorial-email&utm_source=authorletters&utm_campaign=protocols> | - Thank you for this suggestion. The BUSCLAB study protocol has already been published in PLOS ONE: [10.1371/journal.pone.0276613](https://doi.org/10.1371/journal.pone.0276613). Please let us know if you still suggest that the protocol should be deposited at protocols.io. |
|  |  |
| Please ensure that the paper adheres to the PLOS Data Availability Policy (see <http://journals.plos.org/plosmedicine/s/data-availability>), which requires that all data underlying the study’s findings be provided in a repository or as Supporting Information. For data residing with a third party, authors are required to provide instructions with contact information for obtaining the data. PLOS journals do not allow statements supported by “data not shown” or “unpublished results.” For such statements, authors must provide supporting data or cite public sources that include it. | Unfortunately, due to ethical reasons, we are not able to make the underlying data publicly available.  Firstly, the BUSCLAB study was approved by The Regional Committee for Medical and Health Research Ethics (REC) in 2018/2019 (2018/2380), and the written consent form was based on the 2018 version from REC. The General Data Protection Regulation (GDPR) was introduced in Norway on July 20, 2018. Hence, the template used for the written consent form was made prior to the GDPR. Therefore, the participants have not consented to data sharing.  Secondly, the primary endpoint in the BUSCLAB study is time from administration of the investigational medicinal product (IMP) to vaginal delivery. Hence, the date and time of birth of the infant are part of the primary endpoint. We therefore cannot publically share data underlying the primary and several of the secondary endpoints without compromising participant privacy.  Thirdly, combination of the variables underlying the presented data can possibly identify participants and thereby compromise participant privacy.  We therefore suggest the following Data Availability Statement:  Data Availability Statement  There are ethical and legal restrictions on sharing these clinical data. The primary outcome was time from administration of the Investigational Medicinal Product to delivery. The data underlying this outcome contains birth date and time, and cannot be shared. Moreover, combinations of the variables collected could possibly identify participants and thereby compromise participant privacy. In addition, the participants have not consented to sharing of collected data with international researchers according to GDPR recommendations.  Contact email to the study sponsor: [mirnyb@ous-hf.no](mailto:mirnyb@ous-hf.no) |
|  |  |
| **EDITORS** |  |
|  |  |
| **GENERAL COMMENTS** |  |
|  |  |
| Please cite the reference numbers in square brackets (e.g., “We used the techniques developed by our colleagues [19] to analyze the data”). Citations should be preceding punctuation. | We have changed the references to the Vancouver style with square brackets as requested, and we have made sure that all citations are preceding punctuation. |
|  |  |
| Please cite your Supporting Information as outlined here: <https://journals.plos.org/plosmedicine/s/supporting-information> | We have tried to follow the outlined guidelines when citing Supporting information |
|  |  |
| We suggest referring to Buscopan® (Buscopan) as butylscopolamine bromide throughout your manuscript. In general, we recommend using the international nonproprietary name (rINN), which in your case would seem to be hyoscine butylbromide. | We now refer to Buscopan and butylscopolamine bromide as hyoscine butylbromide (HBB) throughout our manuscript, with a few exceptions when using the name Buscopan to name the treatment drug. |
|  |  |
| Please remove the statements following the Acknowledgments from the main text. The data should only be included in the corresponding section in the online submission form. | We have removed the Acknowledgement section from the main text. |
|  |  |
| **ACADEMIC EDITOR COMMENTS** |  |
|  |  |
| Like one of the reviewers, I have never heard of anyone using this treatment so some more information on its use would be helpful. | Please read new text added on p. 5–6 in the Introduction section of the paper: *Antispasmodics are drugs that relieve spasms of smooth muscle tissue, breaking the connection between the parasympathetic nerves and the smooth muscle by acting as antagonists of acetylcholine at muscarinic receptors and thereby inhibiting muscle spasms [24]. In general medicine, the drug is used for reducing spasms in the intestines and urinary tract, abdominal pain of unclear cause, irritable bowel disease, advanced cancer, colonoscopy, radiological examinations, and spasmodic conditions in the female genitalia [25]. The cervix is composed of connective tissue and smooth muscle and innervated by parasympathetic nerve fibers, which constitutes about 50%–60% of the internal cervix [26]. Musculotropic antispasmodics like hyoscine butylbromide (equivalent to butylscopolamine bromide, Buscopan®) directly relax smooth muscles [26]. Antispasmodics are most commonly used during labor in low- and middle-income countries [24].*  *To our knowledge, except from one study from Saudi Arabia [30], there are no studies from high-income countries exploring the effect of hyoscine butylbromide on labor duration. Most previous studies have been conducted in India, Pakistan, Iran, Iraq, Turkey, Nigeria, Mexico, Egypt, and Jamaica [28, 29, 31]*  *We have only found one Egyptian study on the effect of hyoscine butylbromide on slow labor [32].* |
|  |  |
| I also think that it would be useful to present the mean and 95% CI of the difference in labour duration between intervention and control (apologies if they did this and I missed it). They give the hazard ratio. However, the meta-analysis expresses the effect in terms of mean difference. It would help in comparing their results with the meta-analysis to have the mean and 95% CI of their difference. If, for example, the 95% CI of the mean difference in the current study included the point estimate of the mean difference from the meta-analysis, you might conclude that the trial doesn’t tell you very much. | We fully appreciate the Academic Editor’s suggestion to report the difference in mean duration of labor between the two treatment groups to make our results more comparable to those of the cited meta-analysis. Based on crude (i.e., unadjusted) linear regression, the estimated difference in mean duration of labor (in minutes) from administration of the investigational medicinal product (IMP) to any mode of delivery (including emergency cesarean section) between women receiving butylscopolamine bromide (hyoscine butylbromide) and women receiving placebo was -4.62 (95% confidence interval [CI] [-61.50, 52.26]; *p* = 0.873). The corresponding result restricted to women with vaginal delivery was -12.84 (95% CI [-68.29, 42.61]; *p* = 0.649). However, we are a bit hesitant as to whether this should be included in the paper, as this type of analysis does not incorporate the incomplete nature of the data in that time from IMP administration to vaginal delivery is not fully observed for women with emergency cesarean section. Therefore, we are of the opinion that only results based on survival analysis should be reported for the time-to-event outcomes. |
|  |  |
| **EDITOR-IN-CHIEF COMMENTS** |  |
|  |  |
| For the main text, please present the data from the intention-to-treat (ITT) analysis and explain how you accounted for missing follow-up in your analysis. | We thank the Editor-in-Chief for giving us the opportunity to clarify this important issue related to the statistical analysis. The main analysis of each outcome was indeed considered an intention-to-treat (ITT) analysis. The ITT set originally included 250 women. However, one woman did not receive the investigational medicinal product (IMP), that is, neither butylscopolamine bromide (hyoscine butylbromide) nor placebo. The reason why she was not given the IMP was unrelated to the assigned treatment. As the primary outcome of the study was time from IMP administration to vaginal delivery, we chose to exclude her from the ITT set, leaving us with 249 women in the so-called full-analysis (FA) set, which was considered as close as possible to the ITT ideal of including all randomized subjects. Section 5.2.1 in the Statistical Principles for Clinical Trials by the European Medicines Agency (<https://www.ema.europa.eu/en/documents/scientific-guideline/ich-e-9-statistical-principles-clinical-trials-step-5_en.pdf>) discusses the elimination of randomized subjects who took no trial medication: “… The intention-to-treat principle would be preserved despite the exclusion of these patients provided, for example, that the decision of whether or not to begin treatment could not be influenced by knowledge of the assigned treatment …” For all practical purposes, the FA set was identical to the ITT set in the current study, as it included 99.6% (249 / 250) of the women.  Please see the response to Reviewer #3 for more details on this matter. |
|  |  |
| In accordance with the statistical reviewer’s comments, please justify the use of Weibull regression (versus Cox proportional analysis) for your survival analysis and demonstrate that Weibull regression is a valid choice for your study. | In order to detect a real difference in the hazard rate of vaginal delivery between the two treatment groups, we considered the statistical power of the parametric Weibull regression model to be greater than that of the semi-parametric Cox regression model as long as the assumptions of proportional hazard rates and a Weibull-distributed baseline hazard rate were fulfilled. Thus, we chose Weibull regression as the main analysis of each of the three time-to-event outcomes, whereas Cox regression was applied in a sensitivity analysis. However, the results from the two analytical approaches were quite similar, as may be seen in S3 Results, which includes all statistical analyses of the outcomes. For instance, the hazard ratios of vaginal delivery between the two treatment groups based on Weibull regression and Cox regression were 1.00 (95% confidence interval [CI] [0.77, 1.29]; *p* = 0.993) and 0.99 (95% CI [0.76, 1.29]; *p* = 0.941), respectively. The proportional-hazards assumption was tested using Schoenfeld residuals and found valid (*p* ≥ 0.408 for all three time-to-event outcomes). Furthermore, comparing plots of the Nelson-Aalen estimated cumulative hazard function and the fitted cumulative hazard function using Weibull regression for each time-to-event outcome did not indicate any violation against the assumption of the baseline hazard rate following a Weibull distribution. |
|  |  |
| In addition, please provide more details on the clinical background of butylscopolamine bromide in labor (e.g., is it commonly used in labor? Which countries commonly use it?). | Please see new text in answer to Academic Editor, above and in the Introduction section of the manuscript |
|  |  |
| **COMPETING INTEREST STATEMENT** |  |
|  |  |
| All authors must declare their relevant competing interests per the PLOS policy, which can be seen here: <https://journals.plos.org/plosmedicine/s/competing-interests> | All authors have declared relevant competing interests. The form was submitted along with the initial submission. |
|  |  |
| For authors with ties to industry, please indicate whether any of the interests has a financial stake in the results of the current study. | Not applicable |
|  |  |
| **DATA AVAILABILITY STATEMENT** |  |
|  |  |
| PLOS Medicine requires that the de-identified data underlying the specific results in a published article be made available, without restrictions on access, in a public repository or as Supporting Information at the time of article publication, provided it is legal and ethical to do so. Please see the policy at <http://journals.plos.org/plosmedicine/s/data-availability> and FAQs at <http://journals.plos.org/plosmedicine/s/data-availability#loc-faqs-for-data-policy> | Unfortunately, due to ethical reasons, we are not able to make the underlying data publicly available.  Firstly, the BUSCLAB study was approved by the Regional Committee for Medical and Health Research Ethics (REC) in 2018/2019 (2018/2380), and the written consent form was based on the 2018 version from REC. The General Data Protection Regulation (GDPR) was introduced in Norway on July 20, 2018. Hence, the template used for the written consent form was made prior to the GDPR. Therefore, the participants have not consented to data sharing.  Secondly, the primary endpoint in the BUSCLAB study is time from administration of the investigational medicinal product (IMP) to vaginal delivery. Hence, the date and time of birth of the infant are part of the primary endpoint. We therefore cannot publically share data underlying the primary and several of the secondary endpoints without compromising participant privacy.  Thirdly, combination of the variables underlying the presented data can possibly identify participants and thereby compromise participant privacy.  We therefore suggest the following Data Availability Statement: see comment below |
|  |  |
| The Data Availability Statement (DAS) requires revision. For each data source used in your study:  a) If the data are freely or publicly available, note this and state the location of the data: within the paper, in Supporting Information files, or in a public repository (include the DOI or accession number).  b) If the data are owned by a third party but freely available upon request, please note this and state the owner of the data set and contact information for data requests (web or email address). Note that a study author cannot be the contact person for the data.  c) If the data are not freely available, please describe briefly the ethical, legal, or contractual restriction that prevents you from sharing it. Please also include an appropriate contact (web or email address) for inquiries (again, this cannot be a study author). | There are ethical and legal restrictions on sharing these clinical data. The primary outcome was time from administration of the investigational medicinal product to vaginal delivery. The data underlying this outcome contains date and time of birth and cannot be shared. Moreover, combinations of the variables collected could possibly identify participants and thereby compromise participant privacy. In addition, the participants have not consented to sharing of collected data with international researchers according to the General Data Protection Regulation (GDPR) recommendations.  Contact email to the study sponsor: [mirnyb@ous-hf.no](mailto:mirnyb@ous-hf.no) |
|  |  |
| **ABSTRACT** |  |
|  |  |
| Please report your abstract according to CONSORT for abstracts, following the PLOS Medicine abstract structure (Background, Methods and Findings, Conclusions); <https://www.equator-network.org/reporting-guidelines/consort-abstracts/> | We have rewritten the abstract according to the CONSORT guidelines, some text is removed and some new text added:  ***Background:*** *Prolonged labor is a common condition associated with maternal and perinatal complications. The standard treatment with oxytocin for augmentation of labor increases the risk of adverse outcomes such as fetal asphyxia, operative delivery, anal sphincter injuries, postpartum urinary retention, and postpartum hemorrhage. Hyoscine butylbromide is a spasmolytic drug with few side effects shown to shorten labor when used in a general population of laboring women. However, research on its effect on preventing prolonged labor is lacking. We aimed to assess the effect of hyoscine butylbromide on the duration of labor in nulliparous women showing early signs of slow labor.* ***Methods and findings:*** *In this double-blind randomized placebo-controlled trial, we included 249 nulliparous women at term with one fetus in cephalic presentation and spontaneous start of labor showing early signs of prolonged labor by crossing the alert line of the World Health Organization partograph. One-hundred-and-twenty-five participants were randomized to receive 1 mL hyoscine butylbromide (Buscopan®) (20 mg/mL), while 124 received 1 mL sodium chloride intravenously. The trial was conducted at Oslo University Hospital in Norway from May 2019 to December 2021. The primary outcome was duration of labor from administration of the investigational medicinal product (IMP) to vaginal delivery, which was analyzed by Weibull regression to estimate the cause-specific hazard ratio (HR) of vaginal delivery between the two treatment groups, with associated 95% confidence interval (CI). A wide range of secondary maternal and perinatal outcomes were also evaluated. Time-to-event outcomes were analyzed by Weibull regression, whereas continuous and dichotomous outcomes were analyzed by median regression and logistic regression, respectively. All main analyses were based on the intention-to-treat set of women receiving either of the two treatments. The follow-up period lasted during the postpartum hospital stay. All personnel, participants, and researchers were blinded to the treatment allocation. Median (mean) labor duration from IMP administration to vaginal delivery was 401 (440.8) minutes in the hyoscine butylbromide group vs. 432.5 (453.6) minutes in the placebo group.* *We found no statistically significant association between IMP and duration of labor from IMP administration to vaginal delivery: cause-specific HR of 1.00 (95% CI [0.77, 1.29]; p = 0.993). However, we did find an increase in the change in maternal heart rate 30 minutes after IMP administration in the hyoscine butylbromide group compared to the placebo group, with a difference in medians of 3.03 beats/min (95% CI [0.35, 5.71] beats/min; p = 0.027), and a decrease in postpartum hemorrhage, with a difference in medians of -50.00 mL (95% CI [-96.45, -3.55] mL; p = 0.035). Out of 596 eligible women, 249 were included.* ***Conclusion:*** *One intravenous dose of 20 mg hyoscine butylbromide was not found to be superior to placebo in preventing slow labor progress in a population of first-time mothers at risk of prolonged labor. Further research is warranted to answer whether increased and/or repeated doses of hyoscine butylbromide might have an effect on duration of labor.* |
|  |  |
| Please ensure that all numbers presented in the abstract are present and identical to numbers presented in the main manuscript text. | We have ensured that the numbers in the abstract are identical to numbers presented in the main manuscript text. |
|  |  |
| PLOS Medicine requests that main results are quantified with 95% CIs as well as p values.  Please include.  When reporting p values please report as p<0.001 and where higher as the exact p value p=0.002, for example.  For the purposes of transparent data reporting, if not including the aforementioned please clearly state the reasons why not. | All results of the statistical analyses presented in the paper are now reported with 95% confidence intervals as well as *p* values. |
|  |  |
| Please include any important dependent variables that are adjusted for in the analyses. | We did not adjust for any of the dependent variables in the statistical analyses. Since our study was based on data from a randomized controlled trial, the crude (i.e., unadjusted) analyses were regarded as the main analyses of all outcomes but the changes in maternal and fetal heart rates 30 minutes after administration of the investigational medicinal product (IMP), of which the main analyses were adjusted for the corresponding maternal or fetal heart rate at baseline (please see the Statistical Analysis section in the manuscript). As stated in the Statistical Analysis Plan (Section 5.2 in S2 Statistical Analysis Plan), sensitivity analyses adjusting for *maternal age* (categorical variable) and/or *maternal body mass index* (categorical variable) would be conducted for all outcomes in case of any imbalance with respect to either of these two baseline characteristics between women in the two treatment groups. Also, if there appeared to be any imbalance with respect to *cervical dilation before IMP administration* (continuous variable), sensitivity analyses of the time-to-event outcomes of labor duration and the continuous outcomes of cervical dilation rate would be adjusted for this baseline characteristic. However, we found no imbalances of any of these three baseline characteristics, as may be seen in Table 1. Thus, no sensitivity analyses adjusting for either of them were conducted, in accordance with the Statistical Analysis Plan.  Please see the Statistical Analysis section of the paper and the response to Reviewer #3 for more details on this matter. |
|  |  |
| Throughout, suggest reporting statistical information as follows to improve clarity for the reader “22% (95% CI [13%,28%]; p</=)”. Please amend throughout the abstract and main manuscript.  Please note the use of commas to separate upper and lower bounds, as opposed to hyphens as these can be confused with reporting of negative values. | We agree with the Editor-in-Chief that the suggested reporting of results would improve clarity for the reader and have amended the manuscript accordingly. |
|  |  |
| When a p value is given, please specify the statistical test used to determine it. Please report p values as p<0.001 and where higher as ‘p=0.002’ | All *p* values are reported as suggested, and the statistical tests upon which they are based appear from the text. |
|  |  |
| **Abstract Background** |  |
|  |  |
| Provide the context of why the study is important. The final sentence should clearly state the study question. | Please see new text in the abstract: *Hyoscine butylbromide is a spasmolytic drug with few side effects shown to shorten labor when used in a general population of laboring women. However, research on its effect on preventing prolonged labor is lacking. We aimed to assess the effect of hyoscine butylbromide on the duration of the active phase of labor in nulliparous women showing early signs of slow labor.* |
|  |  |
| **Abstract Methods and Findings** |  |
|  |  |
| Please ensure that all numbers presented in the abstract are present and identical to numbers presented in the main manuscript text. | We have ensured that the numbers in the abstract are identical to numbers presented in the main manuscript text. |
|  |  |
| Please include the study design, population and setting, number of participants, years during which the study took place, length of follow up, and main outcome measures. | Please see this new text abstract: *Follow-up period lasted during postpartum hospital stay.*  In addition please see answer to Editor-in-Chief regarding the abstract. |
|  |  |
| Please include the actual amounts and/or absolute risk(s) of relevant outcomes (including NNT or NNH where appropriate), not just relative risks or correlation coefficients. (example for absolute risks: PMID: 28399126). | We welcome the Editor-in-Chief’s suggestion to include absolute (and not just relative) measures. We have chosen to do so for the primary outcome in the main text rather than in the abstract, as this will be provided in a graphical form. Figure 4 displays the respective cause-specific hazard rates of vaginal delivery and emergency cesarean section as functions of time since administration of the investigational medicinal product (in minutes) for the two treatment groups, fitted from Weibull regression in the main analysis.  We have added the following sentence in the third paragraph of the Results section of the paper: “Curves of the respective cause-specific hazard rates of vaginal delivery and emergency cesarean section as functions of time since IMP administration are displayed in Fig 4.” |
|  |  |
| Please include a summary of adverse events if these were assessed in the study. | We thank the Editor-in-Chief for this comment. There were no serious adverse events (SAEs) observed in our study. We registered and graded the adverse events (AEs) according to Common Terminology Criteria for Adverse Events (CTCAE) v5.0. We observed 73 participants with grade 1 (asymptomatic or mild symptoms) AEs and one participant had grade 2 (moderate symptoms) AEs. A more in-depth description of the adverse events together with the remaining secondary outcomes will be published later in 2023/2024. |
|  |  |
| In the last sentence of the Abstract Methods and Findings section, please describe the main limitation(s) of the study’s methodology. | The main limitations of the study are described in the last sentences in the Discussion. Please see the abstract and response to the Editor-in-Chief above. |
|  |  |
| Please specify who was blinded to the intervention and control. | The study participants, the health personnel that treated them and the researchers plotting and analyzing the data were blinded to the treatment allocation |
|  |  |
| Please define the intervention and control states. | Please see the Consort flow chart, the Inclusion and Exclusion criteria |
|  |  |
| Please provide the number in each group. | Please see the revised Consort flow chart |
|  |  |
| Please state that analysis was intention to treat. | We have added the following sentence to the Methods and Findings section of the abstract stating that the main analyses of all outcomes were based on the intention-to-treat set: “All main analyses were based on the intention-to-treat set of women receiving either of the two treatments.” |
|  |  |
| Please provide the number of participants lost to follow up in each group. | There were no participants lost to follow-up in this study. |
|  |  |
| **AUTHOR SUMMARY** |  |
|  |  |
| At this stage, we ask that you include a short, non-technical Author Summary of your research to make findings accessible to a wide audience that includes both scientists and non-scientists. The Author Summary should immediately follow the Abstract in your revised manuscript. This text is subject to editorial change and should be distinct from the scientific abstract. Please see our author guidelines for more information: <https://journals.plos.org/plosmedicine/s/revising-your-manuscript#loc-author-summary>.  The summary should include 2-3 single sentence, individual bullet points under each of the questions.  It may be helpful to review currently published articles for examples which can be found on our website here <https://journals.plos.org/plosmedicine/> | An author summary has been added to the paper. |
|  |  |
| **INTRODUCTION** |  |
|  |  |
| ll.84-85: Please reference the studies referred to in the statement. | We have elaborated more on the disadvantages of prolonged labor and added three new references: number 6, 8 and 9 in this text in the Introduction section:  *In addition to increased risk of operative delivery, prolonged labor is associated with chorioamnionitis [3], a negative birth experience [4, 5], shoulder dystocia [6], low Apgar scores[6], and admission to the neonatal intensive care unit (NICU) [3, 6]. There are also increased risks of severe postpartum hemorrhage [7], anal incontinence [8], urinary retention [9], hematomas [9], and ruptured sutures [9]* |
|  |  |
| ll.84-86: Please revise the two sentences to emphasize the aspect of your study investigating the effect of butylscopolamine bromide on slow labor and not labor in general. | We have rewritten these lines. The new text is to be found in the last paragraph of the Introduction section: *In contrast to previous research on the effect of hyoscine butylbromide on labor duration in general, the double-blind randomized placebo-controlled trial BUSCLAB (BUSCopan in LABor) aimed to assess the effect of hyoscine butylbromide on the duration of the active phase of labor in nulliparous women showing early signs of slow labor.* |
|  |  |
| Please address past research and explain the need for and potential importance of your study (e.g., more details on the implications of prolonged labor, past research on butylscopolamine bromide in labor). Indicate whether your study is novel and how you determined that. If there has been a systematic review of the evidence related to your study (or you have conducted one), please refer to and reference that review and indicate whether it supports the need for your study. | We address previous research and argue why our study is novel by the following text in the last paragraph of the Introduction section:  *To our knowledge, except from one study from Saudi Arabia [30], there are no studies from high-income countries exploring the effect of hyoscine butylbromide on labor duration. Most previous studies have been conducted in India, Pakistan, Iran, Iraq, Turkey, Nigeria, Mexico, Egypt, and Jamaica [28, 29, 31]. We have only found one Egyptian study on the effect of hyoscine butylbromide on slow labor [32].*  We have not found any systematic review related to the effect of hyoscine butylbromide on slow labor progress. However, regarding  the effect on labor duration in general, we refer to three systematic reviews in our paper (reference numbers 23, 27, and 28). |
|  |  |
| **METHODS AND RESULTS** |  |
|  |  |
| Please complete the CONSORT checklist and ensure that all components of CONSORT are present in the manuscript, including [how randomization was performed, allocation concealment, blinding of intervention, definition of lost to follow-up, power statement]. Please include the completed CONSORT checklist as Supporting Information.  When completing the checklist, please use section and paragraph numbers, rather than page numbers. | The CONSORT checklist is completed and refers to section and paragraph for each topic. |
|  |  |
| In accordance with ICMJE requirements, PLOS Medicine requires prospective, public registration of a data sharing plan (as part of mandatory clinical trials registration) for all clinical trials that began enrollment on or after January 1, 2019. | \| Response; Will individual participant data be available (including data dictionaries)? \| No; What data in particular will be shared? \| Not available; What other documents will be available? \| Study Protocol, Statistical Analysis Plan, Informed Consent Form; When will data be available (start and end dates)? \| Not applicable; With whom? \| Not applicable; For what types of analyses? \| Not applicable; By what mechanism will data be made available? \| Not applicable |
|  |  |
| The main analysis should be intention to treat (ie, all individuals randomized are included in the analysis in the groups to which they were originally assigned. If the study included dropouts, specify whether their data are imputed and if so using what method. Please refer to as modified ITT). | Please see the response to the previous comment regarding the intention-to-treat (ITT) analysis.  We have added to the sentence describing the full-analysis (FA) set in the first paragraph of the Analysis Sets section of the paper: “The full-analysis (FA) set comprised all trial participants in the ITT set who received the IMP, thereby excluding trial participants not given the IMP due to reasons unrelated to the assigned treatment. The FA set was thus regarded as a modified ITT set for which the ITT principle was still preserved.” |
|  |  |
| PLOS Medicine requests that main results are quantified with 95% CIs as well as p values.  Please include.  When reporting p values please report as p<0.001 and where higher as the exact p value p=0.002, for example. For the purposes of transparent data reporting, if not including the aforementioned please clearly state the reasons why not. | All results of the statistical analyses presented in the paper are now reported with 95% confidence intervals as well as *p* values. |
|  |  |
| Please include any important dependent variables that are adjusted for in the analyses. | We did not adjust for any of the dependent variables in the statistical analyses. Since our study was based on data from a randomized controlled trial, the crude (i.e., unadjusted) analyses were regarded as the main analyses of all outcomes but the changes in maternal and fetal heart rates 30 minutes after administration of the investigational medicinal product (IMP), of which the main analyses were adjusted for the corresponding maternal or fetal heart rate at baseline (please see the Statistical Analysis section in the manuscript). As stated in the Statistical Analysis Plan (Section 5.2 in S2 Statistical Analysis Plan), sensitivity analyses adjusting for *maternal age* (categorical variable) and/or *maternal body mass index* (categorical variable) would be conducted for all outcomes in case of any imbalance with respect to either of these two baseline characteristics between women in the two treatment groups. Also, if there appeared to be any imbalance with respect to *cervical dilation before IMP administration* (continuous variable), sensitivity analyses of the time-to-event outcomes of labor duration and the continuous outcomes of cervical dilation rate would be adjusted for this baseline characteristic. However, we found no imbalances of any of these three baseline characteristics, as may be seen in Table 1. Thus, no sensitivity analyses adjusting for either of them were conducted, in accordance with the Statistical Analysis Plan.  Please see the Statistical Analysis section of the paper and the response to Reviewer #3 for more details on this matter. |
|  |  |
| Suggest reporting statistical information as detailed above – see under ABSTRACT | We have amended the manuscript according to the suggestions detailed above (under ABSTRACT). All findings in the manuscript are now reported with the appropriate effect measure and the associated 95% confidence interval and *p* value. |
|  |  |
| Please present numerators and denominators for percentages, at least in the Tables [not necessarily each time they’re mentioned]. | The numerators of all percentages in Tables 1 and 2 are presented to their left, whereas the denominators are presented in the respective column headings for all women, women in the butylscopolamine bromide (hyoscine butylbromide) group, and women in the placebo group. |
|  |  |
| Please report the number of patients, and account for all methods used in your study. | All methods used in the study are described in the Statistical Analysis Plan (S2 Statistical Analysis Plan) and in the Statistical Analysis section of the paper. The number of subjects are reported in the Results section of the paper and in the CONSORT flow diagram displayed in Figure 2 in the paper. |
|  |  |
| Please define the length of follow up (eg, in mean, SD, and range). | Women were followed for adverse events from randomization until discharge from hospital after delivery. Unfortunately, we lack information on the date of discharge and must therefore rely on reporting the standard clinical practice.  We have added the following sentence in the third paragraph of the Procedures Following Treatment Administration section of the paper: “All women and infants were followed for adverse events from randomization to discharge from the hospital up to three days after delivery, in accordance with clinical practice.” |
|  |  |
| Please provide the actual numbers of events for the outcomes, not just summary statistics or ORs. | Numbers and percentages of observed levels of all dichotomous variables are included in Table 2 in the paper. We have also added numbers of events of all time-to-event variables to the table. |
|  |  |
| In the trial registry, the secondary outcomes measure ‘changes in maternal and fetal heart rates 30 minutes after IMP administration (in beats/min)” and the secondary dichotomous outcomes are not listed and appear to differ from the primary and secondary outcome measures in the submitted manuscript. Please clarify and explain the discrepancy. If the outcomes were not prespecified in the protocol, please indicate that they were post hoc and explain why they were added. Post hoc comparisons should be presented as hypothesis generating rather than conclusive. | We thank the Editor-in-Chief for giving us the opportunity to clarify this. The outcomes listed in ClinicalTrials.gov was registered before finalizing the statistical analysis plan (SAP). Unfortunately, the information in ClinicalTrials.gov was not updated after the protocol and the SAP were signed.  The description of registration of maternal and fetal heart rates before and 30 minutes after IMP administration is described in the study protocol (S1 Study Protocol), and the statistical analysis is described in the SAP (S3 Statistical Analysis Plan). |
|  |  |
| In addition, the trial registration/protocol lists the outcomes of ‘Pain scores using a Visual Analogue Scale at baseline and 30 minutes after administration of IMP’, ‘Urinary retention, defined as need for urinary catheter before the participants leave the delivery ward’, ‘Anal sphincter injury’, ‘Birth experience measured by the validated questionnaire Child Birth Experience Questionnaire’. (a) Can you please present those results as part of this manuscript, or indicate why that is not possible? (b) Can you please indicate when you plan to publish those results?  Please present the safety data for the study including numbers of specific events and whether or not adverse events are thought to be related to treatment. | We considered that it would be too extensive to provide the results of the statistical analyses of all secondary outcomes in one paper. Hence, results of the statistical analyses of the remaining secondary outcomes and descriptive statistics of adverse events will be reported in a later publication in late 2023 or early 2024.  The safety data include assessments of mother and child during labor, at delivery, and during the stay at the postnatal ward. Some of these outcomes are reported in Table 2 as secondary outcome measures: changes in maternal heart rate, changes in fetal heart rate, pH level of umbilical artery, pH level of umbilical vein, 5- minute Apgar score, 10- minute Apgar score, and admission to the neonatal intensive care unit.  There were no serious adverse events (SAEs) observed in our study. We observed 73 participants with grade 1 (asymptomatic or mild symptoms) adverse events (AEs) and one participant had grade 2 (moderate symptoms) AEs. The analysis of adverse events is not yet completed. We plan on publishing this later in 2023/2024. |
|  |  |
| Please include the study protocol document and analysis plan, with any amendments, as Supporting Information to be published with the manuscript if accepted. | The study protocol is added as a supplementary material (S1 Study Protocol). |
|  |  |
| l.211: “[…] admission of the newborn to the NICU within the first hours after birth.” – In the trial registry, this outcome measure is defined as ‘Within 2 hours after birth’, please revise. | The right term is *the first couple of hours*, meaning the period the mother was still admitted to the labor ward. This text has been revised, see the Methods section, last sentence before the Sample size calculation. |
|  |  |
| ll.332-334: We suggest adding details about the treatment group in which an association for postpartum hemorrhage ≥ 1500 mL in one of the sensitivity analyses was seen | New text has been added in the paragraph before the last paragraph of the Results section*: (one case in the hyoscine butylbromide group and seven cases in the placebo group)* |
|  |  |
| **DISCUSSION** |  |
|  |  |
| Please present and organize the Discussion as follows: a short, clear summary of the article’s findings; what the study adds to existing research and where and why the results may differ from previous research; strengths and limitations of the study; implications and next steps for research, clinical practice, and/or public policy; one-paragraph conclusion. Please remove any subheadings. | We have tried to rearrange the Discussion section according to the Editor-in-Chief’s recommendations. Please see the new version of the paper. |
|  |  |
| l.414: Please add a period at the end of the sentence. | A period has been added to this sentence in the 10^th^ paragraph of the Discussion section: *Our finding of decreased postpartum hemorrhage in the hyoscine butylbromide group is in accordance with previous research by Imaralu et al., who also found a statistically significant reduction in postpartum hemorrhage associated with hyoscine butylbromide [46]* |
|  |  |
| **FIGURES** |  |
|  |  |
| For all Figures, please ensure that you have complied with our figures requirements <http://journals.plos.org/plosmedicine/s/figures>. | Figure 1 is of low quality and is replaced by the World Health Organization partograph. |
|  |  |
| Please provide titles and legends for all figures (including those in Supporting Information files). | We thank the Editor-in-Chief for these comments. We have now included titles and legends for all figures. |
|  |  |
| Please consider avoiding the use of red and green in order to make your figure more accessible to those with colour blindness | We have used the colors blue (Stata color: “navy”) and red (Stata color: “maroon”). Please let us know if this should be changed. |
|  |  |
| In the flow diagram, please indicate the number of individuals in each group analyzed in the ITT analysis. In addition, please change the abbreviation for ‘months’ from ‘mo’ to ‘mos’. | The CONSORT flow diagram has been revised. The 11 participants wrongly included in the trial is now described in a separate box. We hope that this step will clarify the number of participants in each group.  Sadly, we have not been able to identify where the abbreviation for months is written, and consequently, we have not corrected this error. The word “months” only occurs one time in the trial flow diagram and is spelled out in full as “months”. |
|  |  |
| Please in the figure legend/description, define abbreviations used in each figure (including those in Supporting Information files). | Abbreviations are now defined in the figure legends. |
|  |  |
| Figure 1: The quality of Figure 1 is low. | We have replaced Figure 1 with the partograph from the World Health Organization (WHO). The WHO partograph is added as a TIF-file with the correct resolution. |
|  |  |
| Figure 2: Please define ‘NaCl’. | The nomenclature for NaCl in Figure 2 is corrected to sodium chloride. |
|  |  |
| Figure 3: Please add a unit for percentage. | We thank the Editor-in-Chief for this comment. Figure 3 has been edited as suggested. |
|  |  |
| Figure S5: Figure S5 (Supplementary Material S5) appears not be cited in the main text of your manuscript. Please revise. | This figure has been removed. |
|  |  |
| Figure S5: Please define ‘NaCl’. Please change ‘45 unkonown’ to 45 unknown’, ‘1 missing signed consentform’ to 1’ missing signed consent form’, ‘1 not given IMP due to maternal illness not mentioned in exclution criteria’ to ‘‘1 not given IMP due to maternal illness not mentioned in exclusion criteria’, | The information in Figure S5 has been incorporated in the CONSORT flow diagram. Figure S5 has consequently been removed from the revised manuscript. |
|  |  |
| **TABLES** |  |
|  |  |
| Please note the use of commas to separate upper and lower bounds, as opposed to hyphens as these can be confused with reporting of negative values. Suggest reporting statistical information as detailed above – see under ABSTRACT | We have amended the manuscript according to the suggestions detailed above (under ABSTRACT). All findings in the manuscript are now reported with the appropriate effect measure and the associated 95% confidence interval and *p* value. We have used commas to separate upper and lower confidence limits. |
|  |  |
| Please provide titles and legends for all tables (including those in Supporting Information files). | We have provided titles and legends for all tables, which are placed at the end of the manuscript. |
|  |  |
| Please define all abbreviations used in the table below each table (including those in Supporting Information files). | All abbreviations used in a table is placed below the table in question. |
|  |  |
| Table 1: Please remove the p-values presented in Table 1 and the according footnotes. | The *p* values in Table 1 and the associated footnotes have been deleted. |
|  |  |
| Table S4: Please define ‘CTG’, ‘IMP, ‘VAS’ | These abbreviations are now defined in the Supplementary Material section of the paper. |
|  |  |
| **REFERENCES** |  |
|  |  |
| PLOS uses the numbered citation (citation-sequence) method and first six authors, et al.  Please ensure that journal name abbreviations match those found in the National Center for Biotechnology Information (NCBI) databases (<http://www.ncbi.nlm.nih.gov/nlmcatalog/journals>), and are appropriately formatted and capitalised.  Please also see <https://journals.plos.org/plosmedicine/s/submission-guidelines#loc-references> for further details on reference formatting. | The references are changed to the *Vancouve*r style, with square brackets. |
|  |  |
| Where website addresses are cited, please specify the date of access. | The dates have been added to the website addresses cited in this paper. |
|  |  |
| **REVIEWER 1** |  |
|  |  |
| Table 1 - Report either mean or median depending on whether you have a gaussian or non-gaussian distribution. This accounts for several of your variables. | Table 1 has been modified according to the reviewer’s suggestion. Mean and standard deviation are reported for normally distributed variables, whereas median and interquartile range are reported for skewed variables. |
|  |  |
| Table 2 - report either mean or median depending on whether you have a gaussian or non-gaussian distribution. | Table 2 has been modified according to the reviewer’s suggestion. Mean and standard deviation are reported for normally distributed variables, whereas median and interquartile range are reported for skewed variables. |
|  |  |
| Table 2 - Why do you report OR and not RR, when reporting results from an RCT? | Log-binomial regression (with risk ratio / relative risk [RR] as the effect measure) is occasionally hampered by potential convergence problems and risk of overfitting response probabilities to values exceeding 1. Even though this did not seem to be an issue in the current study, we chose to analyze dichotomous outcomes by using logistic regression (with odds ratio [OR] as the effect measure). Thus, we reported ORs instead of RRs in the paper. Nevertheless, findings from log-binomial regression were similar to those of logistic regression, with effect measures in the same direction and almost identical *p* values, as expected. None of the regression analyses of the dichotomous outcomes were statistically significant. |
|  |  |
| Table 2 - suggest information on missing values moved to a footnote below the table, to improve readability. | We acknowledge the reviewer’s suggestion to move numbers and percentages of missing values to a footnote below Table 2 to improve readability. Still, we worry that this will make the information less accessible and more complex, as missing values are reported for as much as 11 of the 23 outcomes – both overall and for each of the two treatment groups. Therefore, we have left this information in the body of the table pending further notice. |
|  |  |
| Table 2 - instead of reporting PPH > 1000 ml yes n (%) or no n(%), simply just report n(%) for the women with pph >1000 ml. This goes for all outcomes. Report the positive findings, so instead of Apgar <7 at 5 minutes - no, please report the actual number and percentage who has a low apgar. | We acknowledge that Table 2 would be condensed (fewer rows) by following the reviewer’s suggestion to restrict the reporting of numbers and percentages in the table to positive findings. However, we feel that is important to report all observed levels of categorical variables (including “no” and “missing”, if appropriate) to ease the reading of the table in that all numbers add up to the total and all percentages add up to 100. We would be happy to revise the table in accordance with the comment if it falls under the requirements of PLOS Medicine. |
|  |  |
| I am not sure why you should adjust for maternal heart rate prior to intervention. Your randomization should have equally distributed your participants into two groups. When looking at your table 1, you have no reason to believe otherwise. | We agree with the reviewer that there are no indications of any imbalance between the two treatment groups with respect to maternal and fetal heart rates at baseline based on the *p* values of the two-sample *t* tests in Table 1 in the paper. Nevertheless, variables of change are usually highly dependent of the baseline level. Therefore, we chose to adjust the statistical analyses of changes in maternal and fetal heart rates 30 minutes after administration of the investigational medicinal product for the corresponding maternal or fetal heart rate at baseline, as this is common practice when analyzing variables of change. This was also prespecified in the Statistical Analysis Plan (Section 5.2 in S2 Statistical Analysis Plan). |
|  |  |
| Figure (no number) and figure 2 should be merged into one diagram. In my point of view no need for two almost identical diagrams. | We thank the reviewer for this suggestion and have incorporated the information in Figure S5 in the CONSORT flow diagram (Figure 2). Figure S5 has consequently been removed from the revised manuscript. |
|  |  |
| **Abstract-Introduction** |  |
|  |  |
| Suggest leaving out some of the comments on oxytocin and focus on the possible effect of buscopan | Some text about oxytocin has been removed, and new text regarding the effect of Buscopan has been added. Please see answer to the Editor-in-Chief. |
|  |  |
| **Abstract - result** |  |
|  |  |
| Only report either mean (SD) or median (IQR) - not both. | We agree with the reviewer that reporting both the mean and the median of the primary outcome might seem a bit excessive. However, we argue that this is necessary, as readers might expect the mean to compare with other studies in addition to the median, which is the most suited summary statistic of a skewed variable, in accordance with the comments above related to Tables 1 and 2. |
|  |  |
| Why do you report HZ and not RR? | We thank the reviewer for allowing us to clarify the findings in the paper. We have reported the hazard ratio (not the risk ratio), as this is the effect measure corresponding to Weibull regression and Cox regression used to analyze the time-to-event outcomes. |
|  |  |
| **Methods** |  |
|  |  |
| I might have missed it, but I could locate any detailed information on recruitment, information of eligible (oral?), when signing the informed consent. I have checked the published protocol as well. However, it seems, you only describe a written information 6 weeks prior to due date. When do they sign the informed consent? Are there any delay between randomization and intervention? Do you have any post randomization dropouts due to a possible delay? | We have added some new text explaining this in the last paragraph of the Inclusion Criteria section: *As the participants were informed about the study in a letter sent to them during pregnancy, some women had signed the consent form before admission to hospital, confirming that they would participate provided labor would be slow and the inclusion criteria for participation were fulfilled. Other participants signed after admission to labor and delivery. All midwives in the labor ward were trained in the study procedures and could provide oral information when needed.*  The intervention took place shortly after the randomization (within 15 minutes to maximum 2 hours), so we did not have any dropouts due to delay. |
|  |  |
| **Results** |  |
|  |  |
| There is no need to include information both the text and, in the consort, flow diagram on recruitment. Suggest that you leave the detailed description to the consort flowchart and shorten the initial text of your result section.  Please consider moving the result on the primary outcome further up in the result section. You write 1.5 page before you report your primary finding. | We have removed some of the text concerning enrollment of participants by adding a subsection describing the analysis sets in the Methods section (please see the Analysis sets section of the paper). Findings of our primary outcome are now presented in the third and fourth paragraphs of the Results section of the paper. |
|  |  |
| **CEQ** |  |
|  |  |
| I cannot find any information on the result of the CEQ. And is the CEQ translated and validated in a Norwegian setting? In the protocol you refer to the original Swedish questionnaire. | The questionnaire is not validated in Norwegian, only in Swedish. We don’t think it is problematic to use it in a Norwegian setting, as people in the two countries understand each other’s language and the obstetric care is very similar. We chose not to elaborate more on this because these findings will be published in a later publication, and we have therefore removed the text about the Childbirth Experience Questionnaire (CEQ). |
|  |  |
| **REVIEWER 2** |  |
|  |  |
| First of all, are spasmolytics really commonly administered drugs during labor (L 76-77)? Could the authors give other references than ref. 22 to support this statement, since I am not sure that this is the case in developed countries (it is certainly not used in the UK or in Denmark). | Thank you for an important comment. You are right in that we have no references on spasmolytics being commonly used during labor, especially not in high-income countries. Some text is removed and new text added. Please see the response to the Academic Editor above and the abstract of the revised manuscript. |
|  |  |
| A very important point in comparing this trial to other trials would be the study population. I am uncertain if this trial actually aims to prevent or treat prolonged labor as the participants are described to “show early signs” of prolonged labor. Does this population differ considerably from that of the previous studies, as the active labor phase was defined from 3 cm cervical dilation, and the mean cervical dilation at inclusion was 5 cm? Deviating from the alert line at 3 or 4 cm is likely to be a common (?) situation in nullips. Could the authors comment on this aspect and refer to studies using the WHO partograph. | We agree with the reviewer that this is an important point and have therefore added new text and research, also to be found in the third paragraph of the Discussion section: *In our study we aimed to include women at risk of prolonged labor by crossing the alert line of the partograph, because these women are more exposed to operative delivery and prolonged labor (crossing the action line of the partograph, 4 hours to the right of the alert line). Research by WHO found that 19.2 % of nulliparous women crossed the alert line and 11.7 % crossed the action line. Among the ones crossing the alert line only, the rate of caesarean section was 5%, and 20% had an operative vaginal delivery. Among the ones also crossing the action line the caesarean section rate was 26% and the vaginal operative rate was 25%. In comparison, for the ones not crossing any lines, the caesarean section rate was 0,7% and the operative vaginal delivery rate 11% .* |
|  |  |
| Please also comment on the use of oxytocin, which was approximately four hours’ duration in each group. Do the authors have information about the cervical dilation at time of oxytocin administration? Please state the number of women who had oxytocin in each group as these women are likely to be “true” prolonged labor parturients. | (215 / 249 = 86.3 %) in the FA-set received oxytocin, (110 / 125 = 88.0 %) in hyoscine butylbromide group and (105 / 124 = 84.7 %) in the placebo group)  New text has been added to the 11th paragraph of the Discussion section:  *Our study provides novel information on oxytocin by measuring the exact dose that was given for labor augmentation; 88.0% (110 / 125) in the hyoscine butylbromide group and (84.7%) (105 / 124) in the placebo group received oxytocin during labor.*  Unfortunately, we do not have information on cervical dilation at the time of oxytocin administration. |
|  |  |
| I can tell from the protocol that the primary outcome was the mean duration from IMP to delivery, bus this is not very clear from this manuscript. Please add “mean” to the primary outcome. | We deeply apologize for the misunderstanding regarding the primary outcome. This is, in fact, time from administration of the investigational medicinal product to vaginal delivery, as specified in the Outcomes section and the Statistical Analysis section of the paper. The primary outcome and the other two time-to-event outcomes were analyzed by using Weibull regression and Cox regression, of which the effect measure is the hazard ratio between the two treatment groups (as opposed to, e.g., the difference in means in linear regression). |
|  |  |
| The nomenclature primiparous can certainly be used to describe women carrying their first child/during their first labor; however, using this term might be confusing, as it is also used to describe women with one previous labor. I suggest to use the word nulliparous to describe the women in this trial so that there is no doubt to the reader. | We have replaced the word primiparous with nulliparous throughout the revised manuscript. |
|  |  |
| Introduction; I suggest to markedly reduce the information on possible oxytocin disadvantages as it takes up too much space and is not the focus of this study. | We have markedly reduced the information on oxytocin disadvantages in the revised manuscript. Please see the second paragraph of the Introduction section. |
|  |  |
| Discussion; This paragraph could overall be much better structured into summary, interpretation of the results (including more general discussion of why this trial is negative as compared to previous trials with significant effect of BB rather than discussing one demographic or outcome at a time), weaknesses (i.e. possible bias in the randomization process with envelopes and using an unblinded person to prepare the medication, precision in including the targeted population including concurrent use of oxytocin) and strengths, generalizability, future research (including moving the discussion about to this place in the discussion as studying multiple administrations is a hypothesis from the trial), and conclusion. | We have tried to rearrange the Discussion section according to your recommendations, new text in the second paragraph: see comment from reviewer 1 and new text: *This procedure contrasts with the procedure at the study hospital, which follows the WHO recommendations of starting oxytocin infusion if four hours have passed without progress.*  *In addition, women giving birth at the study hospital were older and had higher educational level than nulliparous women in general. Hence, the study participants may have represented a selected group of women with reduced external validity of the study as a result.* |
|  |  |
| **Minor points** |  |
|  |  |
| The line “our hospital of 40 mg vs 20 mg was not compared in the meta-analysis” is  unclear as it is stated in the previous lines that the meta-analysis did compare these two doses (L 385). Please revise. | The sentence has been removed. |
|  |  |
| L 388 I suggest to replace “the same” with “our” hospital. | “Same” has been replaced with “our”. |
|  |  |
| L 407-409 The authors should consider if the rationale is the other way around; in a population with a higher risk of prolonged labor (i.e. in this case higher maternal in this study) it could be more difficult to show an effect of the intervention. | We agree that the reviewer has an important comment here and have added the following sentence: *On the other hand, it is possible that such a treatment is not appropriate or sufficient for these women, there might be other reasons for slow progress of labor in this population.* |
|  |  |
| Conclusion: The statement “Larger and/or repeated doses of the medication might reduce duration of labor when labor is prolonged, a hypothesis well suited for further research” (L 450-452) is not a conclusion to be drawn from this data and should be deleted or moved. | The mentioned text is moved to the end of the Discussion section, immediately preceding the Conclusion section. |
|  |  |
| **REVIEWER 3** |  |
|  |  |
| 1) The main analysis (called ‘FA’) is basically complete case analysis. There is no intention to treat analysis at all in the paper. ‘Intention to treat’ means ‘once randomised always analysed’, therefore means analysis of 261 randomised participants which was not possible and not done. The excluded 12 participants may introduce bias to the results as randomisation was disrupted, which is a limitation of the study. | We thank the reviewer for allowing us to elaborate on this important aspect of the statistical analysis. It is true that a total of 261 women were randomized to treatment. However, 10 of the women did not meet the eligibility criteria of the study (not all inclusion criteria and/or at least one exclusion criterion) for some reason, and one woman later withdrew her consent. After conferring with the Clinical Trials Unit at our hospital, it was agreed to replace these women in order to include 250 *eligible* women to comply with the sample size calculation. Thus, only the 250 eligible women constituted the intention-to-treat (ITT) set, as the remaining 11 women should never have been randomized in the first place. We acknowledge that this was not apparent from the original CONSORT flow diagram, which has been edited to better convey this information (Figure 2 in the paper).  Furthermore, one woman in the ITT set did not receive the investigational medicinal product (IMP), that is, neither butylscopolamine bromide (hyoscine butylbromide) nor placebo. The reason why she was not given the IMP was unrelated to the assigned treatment. As the primary outcome of the study was time from IMP administration to vaginal delivery, we chose to exclude her from the ITT set, leaving us with 249 women in the so-called full-analysis (FA) set, which was considered as close as possible to the ITT ideal of including all randomized subjects. For all practical purposes, the FA set was identical to the ITT set in the current study, as it included 99.6% (249 / 250) of the women. It may be regarded as a modified ITT set, restricted to women having received at least one of the treatments to be compared, irrespective of whether the received treatment equaled that being assigned.  Section 5.2.1 in the Statistical Principles for Clinical Trials by the European Medicines Agency (<https://www.ema.europa.eu/en/documents/scientific-guideline/ich-e-9-statistical-principles-clinical-trials-step-5_en.pdf>) discusses the elimination of randomized subjects due to various reasons: “… There are a limited number of circumstances that might lead to excluding randomized subjects from the full analysis set including the failure to satisfy major entry criteria (eligibility violations), the failure to take at least one dose of trial medication and the lack of any data post randomization … The intention-to-treat principle would be preserved despite the exclusion of these patients provided, for example, that the decision of whether or not to begin treatment could not be influenced by knowledge of the assigned treatment …”  Therefore, we humbly argue that the main analysis of each outcome, which was based on the FA set, was not a complete-case analysis but rather an ITT analysis.  We have added to the sentence describing the full-analysis (FA) set in the first paragraph of the Analysis Sets section of the paper: “The full-analysis (FA) set comprised all trial participants in the ITT set who received the IMP, thereby excluding trial participants not given the IMP due to reasons unrelated to the assigned treatment. The FA set was thus regarded as a modified ITT set for which the ITT principle was still preserved.” |
|  |  |
| **Statistical analysis** |  |
|  |  |
| 2 a) Firstly, why using Weibull regression for time to event analysis instead of Cox model? Weibull model requires strong assumption that the change in hazard rate is linear. However we didn’t see this anywhere, then why Weibull model? | In order to detect a real difference in the hazard rate of vaginal delivery between the two treatment groups, we considered the statistical power of the parametric Weibull regression model to be greater than that of the semi-parametric Cox regression model as long as the assumptions of proportional hazard rates and a Weibull-distributed baseline hazard rate were fulfilled. Thus, we chose Weibull regression as the main analysis of each of the three time-to-event outcomes, whereas Cox regression was applied in a sensitivity analysis. However, the results from the two analytical approaches were quite similar, as may be seen in S3 Results, which includes all statistical analyses of the outcomes. For instance, the hazard ratios of vaginal delivery between the two treatment groups based on Weibull regression and Cox regression were 1.00 (95% confidence interval [CI] [0.77, 1.29]; *p* = 0.993) and 0.99 (95% CI [0.76, 1.29]; *p* = 0.941), respectively.  The proportional-hazards assumption was evaluated by visual inspection of log-log survival curves and found valid. It was also formally tested by using Schoenfeld residuals (*p* ≥ 0.408 for all three time-to-event outcomes).  Furthermore, comparing plots of the Nelson-Aalen estimated cumulative hazard function and the fitted cumulative hazard function from the Weibull regression for each time-to-event outcome did not indicate any violation against the assumption of the baseline hazard rate following a Weibull distribution.  We have added the following sentence to the third paragraph of the Results section of the paper: “The assumptions of proportional hazards and Weibull-distributed baseline hazard rate were evaluated and found valid.” |
|  |  |
| 2 b) Competing risk analysis. As the emergency cesarean section is a competing risk event, it’s not adequate just to censor it. This competing risk issue can be addressed in the competing risk framework using, for example, Fine-Gray model. | We thank the reviewer for this insightful comment. We fully appreciate the competing-risks situation related to all of the time-to-event outcomes, which has been addressed both in the statistical analysis plan (S3 Statistical Analysis Plan, Section 5.2.1.1) and in the Outcomes section of the paper. As stated in the statistical analysis plan regarding the primary outcome, the cause-specific hazard ratio (HR) of vaginal delivery between the two treatments, treating emergency cesarean section (ESC) as censoring, would “be evaluated in light of the cause-specific HR of ECS”. To properly take the competing risks into account, we also provided curves of the cumulative incidence of the competing risks for each of the outcomes by applying the *stcompet* command in Stata, which generates cumulative incidence in the presence of competing events.  The proportion of ECSs in the FA set was relatively small (21/ 249 = 8.4%). In addition, there were no indications of increased risk of ECS associated with hyoscine butylbromide, with a cause-specific HR of 1.11 (95% confidence interval [CI] [0.47, 2.62]; *p* = 0.811) in the main analysis of the primary time-to-event outcome and an odds ratio of 1.10 (95% CI [0.45, 2.69]; *p* = 0.835) in the main analysis of the secondary dichotomous outcome. Therefore, the cause-specific HR of vaginal delivery were considered to (approximately) reflect the association between treatment and the cumulative incidence of vaginal delivery.  However, we acknowledge the naïve approach by taking competing events as censored observations. Consequently, we have conducted additional post-hoc competing-risks regression analyses of the three time-to-event outcomes to obtain estimated sub-hazard ratios (SHRs) based on the Fine-Gray method: 1.00 (95% CI [0.77, 1.30]; *p* = 0.990) for time from administration of the investigational medicinal product (IMP) to vaginal delivery, 0.95 (95% CI [0.73, 1.23]; *p* = 0.703) for time from onset of active labor to vaginal delivery, and 0.89 (95% CI [0.69, 1.15]; *p* = 0.392) from IMP administration to cervical dilation of 10 cm.  We have added the following sentence in the fourth paragraph of the Results section of the paper: “Results of a post-hoc competing-risks regression analysis of time from IMP administration to vaginal delivery were similar to the results of the main analysis, with a sub-hazard ratio (SHR) of 1.00 (95% CI [0.77, 1.30]; *p* = 0.990).”  We have also added a sentence to the sixth paragraph of the Results section of the paper: “SHRs obtained from post-hoc competing-risks regression analyses of the secondary time-to-event outcomes were similar to the cause-specific HRs from the main analyses.” |
|  |  |
| 2 c) The primary analysis needs to be adjusted for important/key baseline variables which seems not done in the paper. | We appreciate the reviewer’s call for adjustment for baseline characteristics in the analyses. This matter was discussed with other statisticians at the Clinical Trials Unit at Oslo University Hospital during the preparation of the statistical analysis plan *prior to* unblinding of the data. As the BUSCLAB study is based on data from a randomized controlled trial, we were advised to consider the crude (i.e., unadjusted) analysis as the main analysis for all but three of the outcomes: changes in maternal and fetal heart rates and maternal pain score 30 minutes after administration of the investigational medicinal product (IMP). The main analyses of the first two outcomes would be adjusted for maternal/fetal heart rate at baseline, whereas the main analysis of the last one (to be included in a future publication) would be adjusted for maternal pain score at baseline. Furthermore, sensitivity analyses adjusting for key baseline characteristics would only be presented *provided that* an imbalance with respect to such variables was observed between the two treatment groups. Relevant key baseline characteristics in this regard included *maternal age* (categorical variable), *maternal body mass index* (categorical variable), and *cervical dilation before IMP administration* (continuous variable), as it was conceivable that these variables might affect some of the outcomes. More details on this are provided in the statistical analysis plan (S3 Statistical Analysis Plan, Section 5.2).  We found no imbalance in the key baseline characteristics, as specified in the third paragraph of the Analysis Sets section of the paper: “Descriptive statistics of baseline characteristics of the women in the FA set, overall and by treatment group, are given in Table 1. There were no signs of imbalance between the two treatment groups with respect to any of the baseline characteristics other than slight differences in the continuous variables for maternal weight (Mann-Whitney *U* test: *p* = 0.028) and BMI (Mann-Whitney *U* test: *p* = 0.009). Still, we found no statistically significant association between categorical maternal BMI and treatment group.” Therefore, no adjusted sensitivity analyses were conducted, in accordance with the prespecified statistical analysis plan.  We have also added to a sentence concerning this in the second paragraph of the Statistical Analysis section in the paper: “Since we observed no imbalance in relevant baseline characteristics between the two treatment groups, all analyses were crude except for the analyses of the changes in maternal and fetal heart rates 30 minutes after IMP administration, which were adjusted for maternal and fetal heart rates at baseline, respectively (S3 Statistical Analysis Plan, Section 5.2).”  Nevertheless, a sensitivity analysis of the primary outcome (duration of labor from IMP administration to vaginal delivery) by using Weibull regression and adjusting for the three aforementioned key baseline characteristics displayed similar results as in the main analysis, with a cause-specific hazard ratio (HR) of vaginal delivery of 1.05 (95% confidence interval [CI] [0.80, 1.37]; *p* = 0.718) and a cause-specific HR of emergency cesarean section of 1.22 (95% CI [0.48, 3.12]; *p* = 0.677). The corresponding numbers for Cox regression with the same adjustment were 1.04 (95% CI [0.80, 1.37]; *p* = 0.755) and 0.94 (95% CI [0.36, 2.50]; *p* = 0.907), respectively. |
|  |  |
| **Sample size calculation** |  |
|  |  |
| 3) A short paragraph on sample size with important details is needed in the main paper, rather than just refer to the protocol. | New text about sample size calculations is added before the Analysis Sets section: *The sample size calculations were based on an RCT by Dencker et al. from 2009, which found that among first-time mothers with spontaneous start of labor, the mean duration of labor from randomization to delivery was 5.2 hours, with a standard deviation (SD) of 2.8 hours [36]. A difference in means of 60 minutes in labor duration was considered clinically relevant, as all the other studies on spasmolytic found a difference of more than 55 minutes. With a statistical power of 80% and an SD of 2.8 as given in Dencker et al., our sample size calculations showed that we would require 246 women in total to discover a difference in duration of labor of 60 minutes between the two treatment groups.* |
