# Supplementary material for: The effect of intravenous hyoscine butylbromide on slow progress in labor (BUSCLAB): A double-blind randomized placebo-controlled trial
Source: PLoS Med. 2024 Mar 28;21(3):e1004352. doi: 10.1371/journal.pmed.1004352 (PMC11008832; doi:10.1371/journal.pmed.1004352)
Supplement: Attachment — Submitted filename: Respons to reviewers.docx 10.11.23 (1).docx [file pmed.1004352.s008.docx]

| Comments from | Answers to Editors  and Reviewers |
| --- | --- |
| **Editor**  Per CONSORT, it is essential that you report the safety data for the trial, including the number of specific events and whether the adverse events are considered to be related to treatment (along with details about how the AEs were recorded in the Methods section). The adverse events should be presented in a table unless they are very brief, in which case it is sufficient to include them as a paragraph in the main text (with appropriate statistics). | Information, description, and results from the safety set is now added to *Methods* and *Result* sections. This randomized controlled trial is a part of a PhD project, and three more papers are in progress. Further details from the safety set are described in depth in a paper that will be finalized shortly.  *Abstract*: *Among 255 randomized women having received one dose IMP, a total of 74 women reported a mild adverse event, with 46.5% in the hyoscine butylbromide group and 11.1% in the placebo group (Pearson’s chi-square test: p < 0.001).*  *Methods, Analysis set*, first paragraph, last sentence: *Finally, the safety set comprised all randomized trial participants who ever received the IMP, irrespective of eligibility.  Methods, Analysis set:* Second paragraph: *The safety set included 255 women: the 249 women in the FA set in addition to six non-eligible, erronously randomized women who received one dose of the IMP*.  *Results*, eighth paragraph: *In the safety set, a total of 74 women reported mild grade 1 adverse events, with 46.5% in the hyoscine butylbromide group and 11.1% in the placebo group (Pearson’s chi-square test: p < 0.001).* |
| Regarding the statisticians point about ITT, we suggest that you consider using ‘modified ITT’ to ensure full transparency regarding the participants who were included in the analysis. | We appreciate the Editor’s suggestion for the preferred terminology to be applied in the paper.  To ensure full transparency, we now refer to all eligible, randomized trial participants with a signed informed consent as the *restricted intention-to-treat set*, thereby clarifying that this excludes all non-eligible women erroneously randomized to treatment (10 in total) and women later withdrawing their consent (1 in total) (please see the *Analysis sets* subsection).  Furthermore, we use the term *modified intention-to-treat set* to describe all eligible, randomized trial participants with a signed informed consent who actually received the investigational medicinal product (please see the *Abstract* and the *Analysis sets* subsection). This is identical to the so-called *full-analysis set*, upon which the main analysis of each outcome was based. |
| You have calculated the mean and 95% CI of the difference in the duration of labor, but you do not include it because of the effect of not observing the full duration of labor in women who had an intrapartum cesarean section. While the explanation is appreciated, if you feel that this measure is not interpretable, it may be best to remove the reference to the meta-analysis results where this is the outcome. It would be good to calculate the difference in exactly the same way as was done in the trials that made up the meta-analysis, compare your results with the meta-analysis, and then discuss the limitations of this as an outcome. If you prefer not to present your own results with this measure, it remains rather questionable whether it makes sense to discuss the same measure from previous studies. | We acknowledge and follow the argument of the Editor and have decided to include the difference in mean duration of labor from administration of the investigational medicinal product to delivery between the two treatment groups, both for women with any mode of delivery (including emergency cesarean section) and restricted to women with vaginal delivery (please see the 2^nd^ paragraph of the *Discussion* section). |
| **Reviewer 1**  1. Abstract: The abstract should be further condensed to improve the readability. A suggestion could be to follow the Abstract CONSORT Group. CONSORT for reporting randomised trials in journal and conference abstracts. Lancet. 2008 Jan 26;371(9609):281-3. doi: 10.1016/S0140-6736(07)61835-2. PMID: 18221781. | Please see answers regarding **General editorial points** 1a-d below. |
|  |  |
| 2. Methods: Trial design: line 144 -145 suggest delete - or move: initiate the whole section by stating: further detailed information on the design can be found in the published protocol (ref). | The suggested text: *further detailed information on the design may be found in the published protocol article* has been added and the following has been removed: *Approximately 600 of the women who give birth in this facility are first-time mothers at term with a singleton pregnancy* |
| 3. Result section: You have now deleted all information regarding the participant flow for this section, this is not according to the CONSORT guidance | Text added in second paragraph in the Analysis set section  *From May 2019 to December 2021, 1900 women were assessed for eligibility. A total of 261 women were randomized to treatment. Ten of the randomized women were subsequently excluded due to fulfilling one of the exclusion criteria, and one randomized woman later withdrew her consent, leaving 250 women in the restricted ITT set (126 in the hyoscine butylbromide group, 124 in the placebo group). Furthermore, one woman in the hyoscine butylbromide group did not receive the IMP, and the FA set therefore comprised 249 women (125 in the hyoscine butylbromide group, 124 in the placebo group). The PP set included 248 women (124 in the Hyoscine butylbromide group, 124 in the placebo group), excluding one woman in the hyoscine butylbromide group of whom the midwife was unblinded to the IMP administered.* *The safety set included 255 women: the 249 women in the FA set in addition to six non-eligible, erronously randomized women who received one dose of the IMP. The Consolidated Standards of Reporting Trials (CONSORT) flow diagram of the enrollment of trial participants is shown in Fig 2.* |
| 4. You need to briefly include information on recruitment, randomization and who received intervention and differences/no differences in baseline characteristics. | See answer above (point 3 from the Editor) |
| 5.    Line 371 You report both median and mean. You should only report one, depending on Gaussian or non-gaussian distribution. | We appreciate the Reviewer’s comment regarding the reporting of measures of central tendency. The distribution of duration of labor from administration of the investigational medicinal product to delivery is truly right-skewed, which would suggest reporting the *median*. However, in order to comply with the reporting of other studies, we have chosen also to report the *mean* duration of labor in the two treatment groups, both irrespective of mode of delivery and restricted to vaginal delivery. |
| 6.    Discussion: PPH difference of 50 ml, and maternal heartrate difference of 3 beat/min, are those differences of clinical relevance? | New text added in the end of the 1^st^ paragraph of the Discussion: ..*and not regarded as clinically significant.* |
| 7.    Table 1: For your dichotomous outcome - consider only to report one in the table (not both Yes and No) and the number of missing when relevant. I prefer you report "Yes" | We appreciate the Reviewer’s suggestion to restrict the reporting of frequencies of dichotomous variables to one of the two possible categories only (in addition to “missing” when relevant), as the frequencies of excluded categories are obtainable from the reported numbers.  However, if possible, we would prefer to report the frequencies of *all* observed categories of dichotomous variables in order to follow the same reporting as for categorical variables with more than two levels and also make the frequencies of the observed categories easily accessible to the readers without the need of any additional calculations. |
| 8.    Table 2: Since you have reported n in the first row for each column, I suggest that you leave out "number of events" in the first three rows. It improves the readability and I prefer not to use "events" about the included women. | We thank the Reviewer for this suggestion and have amended Table 2 accordingly. We have also replaced the term “missing” with the term “censoring” when reporting the frequencies of incomplete observations of the three time-to-event outcomes in the table. |
| 9.    For your dichotomous outcome - consider only to report one in the table and the number of missing when relevant | Please see our respond to a similar comment above (7). |
| **Reviewer 3 (statistics)** |  |
| 1) Re intention to treat (ITT), I appreciate authors have argued with great effort and respect, however I am still not convinced. I would like to insist that the analysis is simply not an ITT analysis. Therefore, can't use the name of ITT and also need to discuss this in the limitation. | We agree with the Reviewer on that the analysis is not an intention-to-treat (ITT) analysis in the strict sense. Following the suggestion by the Editor (please see our response to the Editor’s 2^nd^ comment above), we now refer to the full-analysis set, upon which the main analysis of each outcome is based, as a *modified* ITT set (please see the *Abstract* and the *Analysis sets* subsection). This limitation of the study is discussed in the 13^th^ paragraph of the *Discussion* section. |
| 2) Re Weibull regression, although it's fine to use once the assumptions are met, still needs to add one or two sentences to explain why Weibull was chosen over Cox in the main paper as it was not the straightforward first choice in the situation. | We acknowledge that Weibull regression for the main analysis of the three time-to-event variables for duration of labor might not be such a straightforward first choice. We have therefore added a sentence in the 3^rd^ paragraph of the *Statistical analysis* subsection arguing that we might potentially gain power by choosing a parametric survival model. |
| 3) Re 'The primary analysis needs to be adjusted for important/key baseline variables', it is the best practice these days to do the adjustment even if the variables are perfectly balanced as research shows by this way it will narrow down the 95% CI of the estimates (more precise), therefore the adjustment for main analyses is still recommended. | In order to accommodate the Reviewer’s comment regarding adjusted analyses, we have included additional sensitivity analyses in which we adjust for key baseline characteristics. These are included as part of the Supplementary Material *S5 Results* and referred to as post-hoc adjusted sensitivity analyses (please see the 2^nd^ paragraph of the *Results* section and the *Supplementary material* section). |
| **General editorial points**  1.    TITLE: We suggest changing the title to “Evaluation of Intravenous Hyoscine Butylbromide for Slow Progress in Labor (BUSCLAB): A Double-Blind Randomized Placebo-Controlled Trial” | We wish to keep the original title because this is an RCT and we use the term effect in the protocol paper. However, we are willing to reconsider. |
| ABSTRACT  a. Please revise your abstract once more be sure to carefully follow the CONSORT Abstract checklist | We have deleted the sentence: *such as fetal asphyxia, operative delivery, anal sphincter injuries, postpartum urinary retention, and postpartum hemorrhage*  We have added new text regarding the blinding: *Randomization was computer-generated, with allocation concealment by opaque sequentially numbered sealed envelopes* |
| b.    Please only report the primary outcomes of the trial in your abstract. We are in the process of overhauling our workflows and policies (with a new Executive Editor), and we now require that trial abstracts only report secondary outcomes if all secondary outcomes of the trial are included. This is to ensure transparent and unbiased reporting and to avoid placing undue emphasis on specific secondary outcomes. For trials that have many secondary outcomes, such as yours, the abstract should be limited to reporting the primary outcome. | We have deleted this text about secondary outcomes: *However, we did find an increase in the change in maternal heart rate 30 minutes after IMP administration in the hyoscine butylbromide group compared to the placebo group, with a difference in medians of 3.03 beats/min (95% CI [0.35, 5.71] beats/min; p = 0.027), and a decrease in postpartum hemorrhage, with a difference in medians of -50.00 mL (95% CI [-96.45, -3.55] mL; p = 0.035).* |
| c.    Please include a brief summary of the adverse events assessed in the study | Information of the safety set is added to the *Abstract*, *Methods*, and *Result* sections. Please see answer to the Editor above for details. |
| d.    In the last sentence of the Abstract Methods and Findings section, please describe the main limitation(s) of the study's methodology. | New text added: *Women giving birth at the study hospital were older and had higher educational level than nulliparous women in general. More than half of eligible women were not included in the study of various reasonsThe participants may have represented a selected group of women reducing the external validity of the study.* |
| 2.    ABSTRACT:  a.    Please revise your abstract once more be sure to carefully follow the CONSORT Abstract checklist. | Please see answer above (1a-d) |
| 3.    METHODS AND RESULTS:  a.    ll.287-303: This information should be reported in the Results section of the manuscript, rather than the Methods section. | We fully understand that the two paragraphs in question may be regarded as results of the study. However, we humbly argue that they rather form the basis of the statistical analysis. The analyses of the outcomes as described in the subsequent *Statistical analysis* subsection heavily relies on the observed full-analysis set: “Since we observed no imbalance in relevant baseline characteristics between the two treatment groups in the FA set (Table 1), all analyses were crude except for the analyses of the changes in maternal and fetal heart rates 30 minutes after IMP administration, which were adjusted for maternal and fetal heart rates at baseline, respectively (S3 Statistical Analysis Plan, Section 5.2).”  Therefore, we ask, if possible, to keep the paragraphs in the *Analysis sets* subsection to avoid anticipating why the analyses were crude and not adjusted for key baseline characteristics, which was done in a post-hoc adjusted sensitivity analysis instead. |
| 4.    AUTHOR SUMMARY: In the final bullet point of ‘What Do These Findings Mean?’, please describe the main limitations of the study in non-technical language | Deleted text: *The women in this study experienced long labors; overall, the mean duration was 12.4 hours, which is twice as long as the average duration for nulliparous women. The half-life of*  *the drug is short*  *(29 minutes),*  New text: *The effect of Hyoscine butylbromide is short lasting, a single intravenous dose of 20 mg might not have been sufficient to shorten labor in this selected group of nulliparous women.* *Randomized controlled trials assessing the effect of higher or repeated doses of hyoscine butylbromide for women experiencing long labors are needed.* |
| DISCUSSION:  a.    ll. 471-472: For a better understanding, please make it clear that 69 min and 61 min refers to the difference in the first stage of labor compared with placebo (eg, reduced by 69 min…) | New text added in the end of fourth paragraph in the Discussion: *Both doses were shown to shorten the mean duration of the first stage of labor: difference of 69 minutes with a dose of 40 mg compared to placebo vs. 61 minutes with a dose of 20 mg compared to placebo* |
| b.    Causal language - In trials, there is usually a distinction in the language in terms of causal vs associational for primary and secondary trial outcomes, respectively. It would be preferable to use associational language in the discussion( and other sections) when referring to secondary outcomes (e.g., l.520). | We agree on the distinction between causal language for the primary outcome and more associational language for secondary and exploratory outcomes. We only refer to associations, or lack thereof, when reporting the results of secondary outcomes in the *Results* section. In the *Discussion* section, we have changed the wording somewhat by referring to associations instead of effects for both of the secondary time-to-event outcomes (1^st^ paragraph), amount of infused oxytocin (11^th^ paragraph), and mode of delivery (14^th^ paragraph). |
| c.    Please remove the ‘Conclusion’ subheading from the discussion, as the one-paragraph conclusion should be part of the discussion section. | We have now removed this subheading in the *Discussion* section. Furthermore, the two original paragraphs constituting the conclusion have been combined into one. |
| 6.    Figure 4: Are the lines for the hazard rates (vaginal delivery) of hyoscine butylbromide and placebo congruent (only one line is visible)? | Yes, that is correct. The two lines are completely overlapping. |
| 7.    Table 1: In the second row (Maternal age (in years)), please either include the unit in parentheses (“years”) and then remove "years" from each age group in the list below; OR remove the unit from the heading and keep "years" as the unit with each age group. | We have kept the unit of maternal age in parentheses in the heading and thus removed it from each of the maternal age categories in Table 2. |
| 8.    Table 1: Please note that the footnote ‘6’ is not formatted as superscript. | Footnote 6 in Table 2 has now been formatted as superscript. |
| 9.    SUPPLEMENT, S2 Trial Flow Diagram: I cannot locate footnote ‘e’ in the figure description? | The description had fallen out and we have now added: *e Physical examination newborn included an examination of general appearance.* |
| 10.    REFERENCES: Where website addresses are cited, when specifying the date of access please replace the word ‘cited’ with ‘accessed’. |  |
| 11.    Please add the following statement, or similar, to the Methods: "This study is reported  as per the Consolidated Standards of Reporting Trial (CONSORT) guideline (S1 Checklist)." | The suggested text is added as the first sentence of the *Methods* section. |
| 12.    Please remove the statements following the Acknowledgments from the main text (Declaration of interest, Role of the funding source, Data sharing). The data should only be included in the corresponding section in the online submission form. | The text is removed from the main text and will be included in the corresponding section in the online submission form. |
| 13.    Please include this information “Contact email to the study sponsor: [mirnyb@ous-hf.no](mailto:mirnyb@ous-hf.no)” in the Data availability statement in the manuscript submission form | The text is removed from the main text and will be included in the Data availability statement in the manuscript submission form. |
| 14.    If you have not already done so, please upload any figures associated with your paper as individual TIF or EPS files with 300dpi resolution at resubmission; please read our figure guidelines for more information on our requirements: <http://journals.plos.org/plosmedicine/s/figures>. While revising your submission, please upload your figure files to the PACE digital diagnostic tool, <https://pacev2.apexcovantage.com/>. PACE helps ensure that figures meet PLOS requirements. To use PACE, you must first register as a user. Then, login and navigate to the UPLOAD tab, where you will find detailed instructions on how to use the tool. If you encounter any issues or have any questions when using PACE, please email us at [PLOSMedicine@plos.org.](mailto:PLOSMedicine@plos.org.) | All figures have been uploaded to PACE as recommended.  Fig1 is edited and the PACE tool has set the resolution to 500dpi. The size is according to recommended dimension. Please let us know if this is not sufficient. |
| 15.    We ask every co-author listed on the manuscript to fill in a contributing author statement, making sure to declare all competing interests. If any of the co-authors have not filled in the statement, we will remind them to do so when the paper is revised. If all statements are not completed in a timely fashion this could hold up the re-review process. If new competing interests are declared later in the revision process, this may also hold up the submission. Should there be a problem getting one of your co-authors to fill in a statement we will be in contact.  YOU MUST NOT ADD OR REMOVE AUTHORS UNLESS YOU HAVE ALERTED THE EDITOR HANDLING THE MANUSCRIPT TO THE CHANGE AND THEY SPECIFICALLY HAVE AGREED TO IT. You can see our competing interests policy here: <http://journals.plos.org/plosmedicine/s/competing-interests>. |  |
| 16.    To enhance the reproducibility of your results, we recommend that you deposit your laboratory protocols in protocols.io, where a protocol can be assigned its own identifier (DOI) such that it can be cited independently in the future. Additionally, PLOS ONE offers an option to publish peer-reviewed clinical study protocols. Read more information on sharing protocols at <https://plos.org/protocols?utm_medium=editorial-email&utm_source=authorletters&utm_campaign=protocols> | Please see response below (17) |
| 17.    Please ensure that the paper adheres to the PLOS Data Availability Policy (see http://journals.plos.org/plosmedicine/s/data-availability), which requires that all data underlying the study's findings be provided in a repository or as Supporting Information. For data residing with a third party, authors are required to provide instructions with contact information for obtaining the data. PLOS journals do not allow statements supported by "data not shown" or "unpublished results." For such statements, authors must provide supporting data or cite public sources that include it. | Unfortunately, due to ethical reasons, we are not able to make the underlying data publicly available.  Firstly, the BUSCLAB study was approved by The Regional Committee for Medical and Health Research Ethics (REC) in 2018/2019 (2018/2380), and the written consent form was based on the 2018 version from REC. The General Data Protection Regulation (GDPR) was introduced in Norway on July 20, 2018. Hence, the template used for the written consent form was made prior to the GDPR. Therefore, the participants have not consented to data sharing.  Secondly, the primary endpoint in the BUSCLAB study is time from administration of the investigational medicinal product (IMP) to vaginal delivery. Hence, the date and time of birth of the infant are part of the primary endpoint. We therefore cannot publically share data underlying the primary and several of the secondary endpoints without compromising participant privacy.  Thirdly, combination of the variables underlying the presented data can possibly identify participants and thereby compromise participant privacy.  We therefore suggest the following Data Availability Statement:  Data Availability Statement  There are ethical and legal restrictions on sharing these clinical data. The primary outcome was time from administration of the Investigational Medicinal Product to delivery. The data underlying this outcome contains birth date and time, and cannot be shared. Moreover, combinations of the variables collected could possibly identify participants and thereby compromise participant privacy. In addition, the participants have not consented to sharing of collected data with international researchers according to GDPR recommendations.  Contact email to the study sponsor: [mirnyb@ous-hf.no](mailto:mirnyb@ous-hf.no)  Contact email to the study Principal Investigator: trmi1@ous-hf.no |
