# Supplementary material for: The effect of intravenous hyoscine butylbromide on slow progress in labor (BUSCLAB): A double-blind randomized placebo-controlled trial
Source: PLoS Med. 2024 Mar 28;21(3):e1004352. doi: 10.1371/journal.pmed.1004352 (PMC11008832; doi:10.1371/journal.pmed.1004352)
Supplement: Attachment — Submitted filename: Answer to reviewers 190124.docx [file pmed.1004352.s009.docx]

| Comments from reviewers | Response |
| --- | --- |
| As discussed, please report the full safety data for the trial, including the number of specific events and whether the adverse events are considered to be related to treatment (along with details about how the AEs were recorded in the Methods section). The adverse events should be presented in a table unless they are very brief, in which case it is sufficient to include them as a paragraph in the main text (with appropriate statistics). | The safety set is included in the paper and reported in the abstract, results- and discussion section, with an additional table*.*  **Abstract, methods and findings, second to last paragraph:**  *Among 255 randomized women having received one dose of IMP, 169 women (66.3%) reported a mild adverse event: 75.2% in the hyoscine butylbromide group and 57.1% in the placebo group (Pearson’s chi-square test: p = 0.002).*  **Results, last paragraph:**  *We observed 169 women (66.3%) in the safety set with any adverse event across all system organ classes (234 adverse events): 97 women (75.2%) in the hyoscine butylbromide group (146 adverse events) and 72 women (57.1%) in the placebo group (88 adverse events) (Pearson’s chi-square test: p = 0.002). The numbers and percentages of women with adverse events, by system organ class, are presented in Table 3 for both treatment groups.*  **Discussion, second to last paragraph:**  *A substantial number of self-reported and observed mild adverse events were noticed, with a significantly higher occurrence of heart palpitations (grouped as cardiac disorders) and blurry vision (grouped as eye disorders) in the hyoscine butylbromide treatment group. These adverse events align with well-established common side effects of hyoscine butylbromide. However, it is imperative to note that the majority of reported adverse events primarily encompassed urinary retention, a frequently observed condition following prolonged labor involving high frequency of epidural anesthesia and operative vaginal delivery. There were no differences in rates of urinary retention in the hyoscine butylbromide and placebo groups, and such events cannot necessarily be directly attributed to the administration of hyoscine butylbromide.*  **New table**: *Table 3: Numbers and percentages of women with adverse events by system organ class, overall and by treatment group.* |
| DATA AVAILABILITY  Thank you for providing the Data Availability Statement (DAS) statement. In line with PLOS’ policy on data availability, we kindly ask you to share a “minimal data set”. PLOS defines the “minimal data set” to consist of the data set used to reach the conclusions drawn in the manuscript with related metadata and methods, and any additional data required to replicate the reported study findings in their entirety. Authors do not need to submit their entire data set, or the raw data collected during an investigation. If possible, please submit the following data:  The values behind the means, standard deviations and other measures reported;  The values used to build graphs. | Unfortunately, due to ethical reasons, we are not able to make the underlying data publicly available.  Firstly, the BUSCLAB study was approved by The Regional Committee for Medical and Health Research Ethics (REC) in 2018/2019 (2018/2380), and the written consent form was based on the 2018 version from REC. The General Data Protection Regulation (GDPR) was introduced in Norway on July 20, 2018. Hence, the template used for the written consent form was made prior to the GDPR. Therefore, the participants have not consented to data sharing.  Secondly, the primary endpoint in the BUSCLAB study is time from administration of the investigational medicinal product (IMP) to vaginal delivery. Hence, the date and time of birth of the infant are part of the primary endpoint. We therefore cannot publically share data underlying the primary and several of the secondary endpoints without compromising participant privacy.  Thirdly, combination of the variables underlying the presented data can possibly identify participants and thereby compromise participant privacy. |
| ABSTRACT  1) ll.55-56 “Women giving birth at the study hospital were older and had higher educational level than nulliparous women in general.” – This detail may be more appropriate for inclusion in the Results/Discussion section rather than the Abstract. If stated in the Results/Discussion section, please provide reference.  2) l. 57 “...in the study of various reasons.” - Please specify “various reasons”. Also, according to the flow chart, more than 80% of eligible women were excluded. Editorial suggestion: “More than 80% of eligible women were excluded from the study because they did not meet the inclusion criteria.” Please make it clear that this is the main limitation(s) of the study’s methodology; the phrase “The main limitation of the study...” may be useful. | 1) We have removed the sentence *“Women giving birth at the study hospital were older and had higher educational level than nulliparous women in general.”* from the Abstract. The sentence *“In addition, women giving birth at the study hospital were older than nulliparous women in general.”* is added in the last page of the Discussion section and a reference has been added*.*  The part about education level has been removed as we were not able to find an appropriate reference.  2) We will argue that it is not correct that more than 80% of eligible participants were not included. It is correct that more the 80% of women invited and informed about the study were not included, but as women were invited several weeks before their due date, a large proportion of these women were never eligible because their labors were induced, they gave birth prematurely or by cesarean delivery. According to the flow chart, it is correct that approximately 50% of eligible women were not included. We have added the following to the sentence to explain what we mean by “various reasons”: “… because they did not wish to participate or were not included upon admission.” |
| AUTHOR SUMMARY  1) Under ‘What Did the Researchers Do and Find?’, please split the two bullet points into four to make the details provided more accessible. Editorial suggestion:  What Did the Researchers Do and Find?  •    We performed a double-blind randomized placebo-controlled study, including 249 nulliparous women showing first signs of slow labor progress.  •    The participants were randomized to receive either a single intravenous dose of the spasmolytic drug hyoscine butylbromide (Buscopan®) (20 mg) or a single intravenous dose of saline solution (placebo).  •    We found no statistically significant difference in labor duration between the two treatment groups. There was a decrease in postpartum hemorrhage and a slight increase in maternal heart rate in the hyoscine butylbromide group.  •    No maternal serious adverse events were observed, nor did we observe any neonatal adverse events.  2) Under ‘What Do These Findings Mean?’, the first bullet point seems to repeat an observation rather than being an interpretation of the finding. Editorial suggestion: Hyoscine butylbromide was not found to impact duration of labor from treatment administration to delivery for first-time mothers with long labors.  3) The last bullet under ‘What Do These Findings Mean?’ point should only describe the main limitation of the study’s methodology. The sentence starting “Randomized controlled trials assessing the effect of higher or…” may be added as a separate bullet point prior to the limitation. | 1) We have split the two bullet points into four.  2) We have replaced the sentence: *We observed no effect of hyoscine butylbromide on duration of labor from treatment administration to delivery for women with long labors*. With:  *Hyoscine butylbromide was not found to impact duration of labor from treatment administration to delivery for first-time mothers with long labors.*  3) The sentence: *Randomized controlled trials assessing the effect of higher or repeated doses of hyoscine butylbromide for women experiencing long labors are needed* has been added as a separate bullet point prior to the limitation. |
| METHODS AND RESULTS  1) At the beginning of the Results section, please include a few sentences about the study population, i.e., the number screened for eligibility, the number deemed eligible, the number not enrolled, etc. (according to the flowchart).  2) ll. 375-377 “…was markedly higher..”: Did you test for significance? If not, please temper language.  3) l.408: Please use causal language for the description of primary outcomes. In trials, there is usually a distinction in the language in terms of causal vs associational for primary and secondary trial outcomes. It would be beneficial to use associational language in the discussion and other sections for secondary outcomes. | 1) At the beginning of the Result section the following has been added: *Out of 1900 first time mothers invited during pregnancy, 1121 did not meet the inclusion criteria, 138 delivered at another hospital and for 45 women the reason for not participating was unknown. Of the 596 eligible women, 100 did not wish to be included, and 235 were not included upon admission. The remaining 261 were included in the study, 11 were later excluded, because they did not fulfill the inclusion criteria (details in figure 2)*  2) We did not test for significance. Therefore, we have removed the word *markedly*  3 As the sentence in question concerns the two *secondary* time-to-event outcomes, we have used *associational* language by referring to associations instead of effects. To avoid misunderstandings, we have chaged: *As for the primary outcome* to: *Similar to the analysis of the primary outcome* |
| DISCUSSION  1) ll.446-449: Please use causal language for the description of primary outcomes.  2) ll.474-481: Please provide reference for the first and last sentence of the paragraph if referring to the same reference.  3) l.558: Please specify ‘various reasons’.  4) l.564: “In addition, women giving birth at the study hospital were older and had higher educational level than nulliparous women in general.” - Please provide reference. | 1) As the sentence in question concerns *secondary* time-to-event and continuous outcomes, we have used *associational* language by referring to associations and differences instead of effects. In the preceding sentence (i.e., the first sentence of the Discussion section), however, we have used *causal* language, as this concerns the *primary* outcome.  2) The correct reference has been provided after the first sentence  3) *various reasons* has been replaced with: *100 did not wish to participate in the study and 235 were not included upon admission*  4) Reference is added |
| REFERENCES  Please thoroughly revise all references and ensure that journal name abbreviations match those found in the National Center for Biotechnology Information (NCBI) databases (<http://www.ncbi.nlm.nih.gov/nlmcatalog/journals>), and are appropriately formatted and capitalised (e.g., for reference [17] BMC Pregnancy and Childbirth should be BMC Pregnancy Childbirth) | We have tried to revise all references according to your recommendations |
| FIGURES  1) Figure 2: Please remove any CONSORT labeling. The top of the flowchart should show the enrollment/assessment of eligibility box.  2) Figure 3: Please remove the headings ‘Absolute frequency’ and ‘Relative frequency’.  3) Figure 4: Please ensure to present 95% confidence intervals in the figure.  4) Figure 4: Please note that the lower graph of Figure 4 is titled ‘emergency cesarean section delivery’ while in other figures you use the title ‘emergency cesarean section’. Please revise.  5) Figure 3-7: Please in the figure description, add details about the dose of hyoscine butylbromide, the definition of placebo as well as the definition of ‘emergency cesarean section’. | 1) Figure 2: CONSORT labeling on the top is removed. New TIFF file uploaded. 2) The headings in Figure 3 have been removed. 3) The plots of the cause-specific hazard rates of vaginal delivery and emergency cesarean section as functions of time since administration of the investigational medicinal product in Figure 4 were generated by the *stcurve* command in Stata. Unfortunately, this command does not offer an option to include a confidence interval. 4) The title of the lower plot in Figure 4 has been revised to correspond with the remaining figures. 5) Details about the dose of hyoscine butylbromide, the definition of placebo, and the definition of emergency cesarean section have been added to the figure legends of Figures 3–7. |
| SOCIAL MEDIA  To help us extend the reach of your research, please provide any X (formerly known as Twitter) handle(s) that would be appropriate to tag, including your own, your coauthors’, your institution, funder, or lab. Please respond to this email with any handles you wish to be included when we tweet this paper. | Unfortunately, we do not have any X handles suitable for this purpose |
